# Phage-Display Immunoprecipitation Sequencing Reveals Distinct Antibody Signatures Against Bacterial Flagellins Associated With Treatment Response in Crohn's Disease

**DOI:** 10.14309/ctg.0000000000001030

**Published:** 2026-04-02

**Authors:** Haim Leibovitzh, David Krongauz, Yishay Schlesinger, Nathaniel Aviv Cohen, Ayal Hirsch, Yulia Ron, Tamar Thurm, Anastasia Godneva, Adina Weinberger, Eran Segal, Nitsan Maharshak

**Affiliations:** 1 Department of Gastroenterology and Liver Diseases, Tel Aviv Sourasky University Medical Center, Tel Aviv, Israel;; 2 Gray Faculty of Medical and Health Sciences, Tel Aviv University, Tel Aviv, Israel;; 3 Department of Computer Science and Applied Mathematics, Weizmann Institute of Science, Rehovot, Israel;; 4 Department of Molecular Cell Biology, Weizman Institute of Science, Rehovot, Israel.

**Keywords:** Crohn's disease, phage-display immunoprecipitation sequencing, bacterial flagellins, microbiome

## Abstract

**INTRODUCTION:**

Enhanced immune response against bacterial flagellins among patients with Crohn's disease (CD) is associated with aggressive disease course. However, its association with response to biologic treatment is unknown. We aimed to assess whether treatment response among patients with CD is associated with antibody reaction to bacterial flagellins and related microbial alterations.

**METHODS:**

Thirty-nine patients with active CD (Harvey-Bradshaw Index > 4 or fecal calprotectin > 150 μg/g) commencing biologic treatment were included. Serum and stool samples were collected at baseline and during treatment at weeks 14, 22, and 52. Serum samples were analyzed using high-throughput phage-display immunoprecipitation sequencing (PhIP-Seq) and fecal samples by DNA shotgun metagenomic sequencing.

**RESULTS:**

Using PhIP-Seq analysis, only the antiflagellin antibodies' library showed consistently attenuated antibody responses against bacterial flagellins in patients achieving remission (Harvey-Bradshaw Index ≤ 4 and fecal calprotectin ≤ 150) vs nonremission at all time points (*P* < 0.05). Of the 55 antiflagellin antibodies analyzed, 15 showed consistent >1.5-fold overrepresentation in nonremission samples, with high conservation of amino-acid sequences and targeting of *Clostridiales*, *Lachnospiraceae*, or *Roseburia* species. Remission was associated with increased abundance of flagellin-target taxa including *Roseburia intestinalis* and decreased *Ruminococcus_B gnavus* and pathways involved in cellular oxidative stress, while nonremission showed increased *Bacteroides* species and pathways involved in 5-aminoimidazole ribonucleotide and semiessential amino acids biosynthesis (q < 0.05).

**DISCUSSION:**

PhIP-Seq revealed that biologic treatment response in patients with CD associates with consistently decreased antibody responses against specific bacterial flagellins with conserved sequences. These findings identify potential biomarkers and therapeutic targets for improving treatment outcomes.

## Graphical abstract

**Figure:**
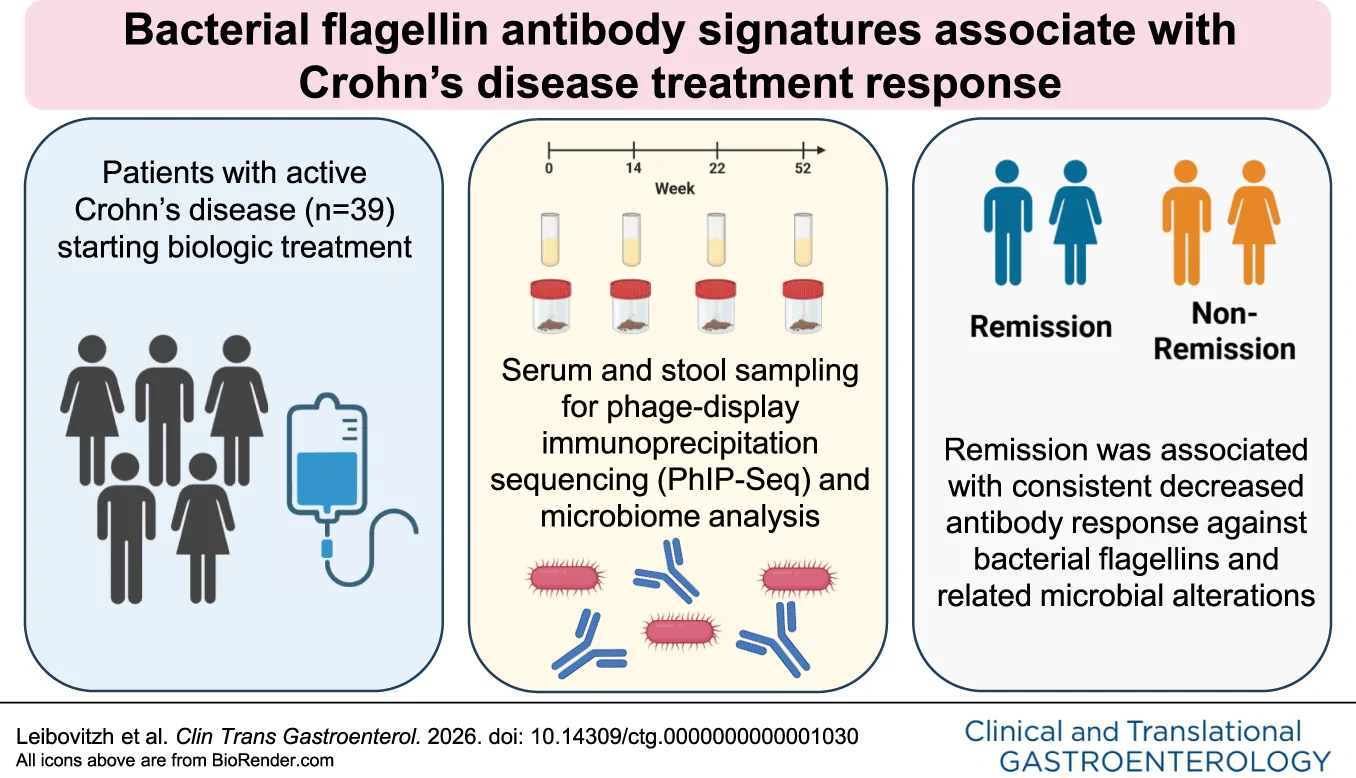

## INTRODUCTION

Crohn's disease (CD) is a chronic relapsing and remitting inflammatory disease of the gastrointestinal tract (1). It is hypothesized that alterations in intestinal permeability, gut microbial composition, and immune response to bacterial antigens are involved in the inflammatory cascade underlying CD pathogenesis (2). To treat the inflammation and to prevent structural bowel damage, CD often necessitates immune-mediated therapy (3). However, despite the variety of advanced therapies available, response rates in CD are still between 30% and 60% (4), and currently, biomarkers predicting treatment response, such as the gut microbiome, are lacking or show limited performance (5).

Bacterial flagellins are protein subunits that make up the flagellar filament, which has several roles including bacterial motility. Recognition of flagellins through toll-like receptor-5 (TLR-5) as pathogen-associated molecular patterns, trigger innate immune responses leading to production of proinflammatory cytokines, which are crucial in the host defense against bacterial infections (6,7). Previous studies have shown that the immune response against bacterial flagellins plays a role in CD pathogenesis (8,9) and has been associated with a more aggressive disease course (10). Specifically, antiflagellin antibody responses in CD were shown to be targeted to bacterial taxa belonging to the order of *Clostridiales* and the family of *Lachnospiraceae*, including genera such as *Roseburia* and *Agathobacter* as well as other pathogenic bacteria including pathogenic species such as *Salmonella* and *Burkholderia* (7,8). However, data on whether there is an association between immune responses to bacterial flagellins and response to biologic treatment is lacking.

In this study, we used serum high-throughput phage-display immunoprecipitation sequencing (PhIP-Seq) and fecal shotgun metagenomic sequencing to assess whether response to biologic treatment in patients with CD is associated with immune reactions against bacterial flagellins and with related microbial composition and function.

## MATERIALS AND METHODS

### Patient population

Patients were prospectively recruited (January 2018 to June 2021) at a single tertiary center in the Inflammatory Bowel Diseases unit of the Tel-Aviv Medical Center, Israel. The Tel Aviv Sourasky ethics committee approved the study (IRB# 0162-19-TLV); informed consent was obtained.

Patients with active CD, defined as either Harvey-Bradshaw Index > 4 or fecal calprotectin (FCP) > 150 μg/g, with no previous exposure to biologic treatment, commencing treatment with either an anti-tumor necrosis factor (anti-TNF), vedolizumab, or ustekinumab, were recruited to the study. Subjects with gastrointestinal stoma or pregnancy were excluded. Baseline demographics and clinical parameters were recorded. Serum and fecal samples were collected at baseline (week 0, before treatment initiation) and at each follow-up visit. To accommodate clinical scheduling, samples were collected within predefined time windows: week 14 (weeks 12–16), week 22 (weeks 19–26), and week 52 (weeks 48–56) after treatment initiation, corresponding to end of induction, early maintenance, and late maintenance phases, respectively. Remission was defined as Harvey-Bradshaw Index ≤ 4 and FCP ≤ 150 μg/g.

### Serum sampling, library preparation, and processing for PhIP-Seq

Serum samples were collected at weeks 0, 14, 22, and 52. Owing to technical issues, only 5 subjects had baseline serum (week 0) samples sequenced, and therefore, these samples were excluded, and only samples from weeks 14, 22, and 52 were analyzed.

PhIP-Seq used 344,000 peptides from 2 libraries: Agilent microbiota antigen library (244,000 peptides) and Twist library (100,000 peptides). More detailed information on the antigen library composition, antigen processing, library cloning, and specific sequencing protocols can be found in the previously published work from our laboratory (8).

The 344,000 peptides comprising the complete antigen library were filtered to identify those significantly bound in at least 20% of the cohort, resulting in 759 epitopes for subsequent analysis. The number of peptides in each of the subgroups was as follows: complete library—759, microbiome—264, immunoglobulin-A—17, probiotic—25, flagellins—55, virulence factor database—155, phages—6, allergens—13, immune epitope database controls—320, and autoimmune—67. PhIP-Seq data were processed by calculating fold change as previously described (11).

### Stool sampling, DNA extraction, and metagenomic sequencing

At each visit, fecal samples were collected, aliquoted, and frozen at −80°C. DNA was extracted using a PowerMag Soil DNA isolation kit and sequenced on Illumina platforms (12,13). Reads were processed with Trimmomatic (14) and mapped using Bowtie2 (15). All samples were subsequently downsampled to a depth of 5M mapped reads.

HUMAnN3.6 identified taxonomic and functional profiles using MetaPhlAn3.0 and UniRef90 database (16–18).

### Statistical analysis

For the PhIP-Seq analysis, differences in antibody responses across the 3 time points between remission and nonremission states were calculated using Mann-Whitney *U* test with Benjamini-Hochberg correction for multiple testing.

For the microbiome analysis, alpha diversity (Shannon index) was analyzed using the Wilcoxon signed-rank sum test. Beta diversity (Bray-Curtis dissimilarity index) was assessed with principal coordinates analysis plots. Permutation analysis of variance was applied using the adonis function on distance matrices with 1,000 permutations. Subject IDs were included as strata to account for the paired pretreatment and posttreatment samples from the same subjects (vegan [v2.5-7] R package). Species and pathway differential abundances were assessed through a multivariable linear mixed-effects model (multivariate analysis by linear models [v1.4.0] R package [19]) adjusted for age, sex, and body mass index. Subject IDs were included in the model as a random effects. The model filtering parameters were set to include only features with minimum prevalence of 30% and minimum abundance higher than 0.01%. Significance was set for false discovery rate (FDR) values < 0.05 (19,20).

The Spearman rank-order correlation test was used (SciPy library) to explore the relationship between serum flagellin peptides (15 selected flagellins peptides with a consistent ratio of >1.5-fold overrepresentation in nonremission versus remission at all time points) and fecal microbial species and metabolic pathways (derived from multivariate analysis by linear models; FDR < 0.05).

## RESULTS

### Cohort description

Thirty-nine biologic naïve patients with CD were included: 48.7% (n = 19) women, median age 33.1 (interquartile range [27.0–46.5]). Disease duration was 9.0 years [4.0–18.5], with predominantly ileal (48.7%) or ileocolonic (41.1%) inflammatory phenotype (48.7%). Baseline FCP was 369.0 μg/g (222.5–724.5). Anti-TNF was initiated in 56.4% of patients, whereas 23.1% initiated vedolizumab and 20.5% ustekinumab (Table 1).

**Table 1.** Cohort baseline demographics and clinical data

Baseline characteristics	Patients with CD (N = 39)
Sex, females (%)	19 (48.7%)
Age, median years (IQR)	33.0 (27.0; 46.5)
BMI, median kg/m^2^ (IQR)	21.9 (19.6; 24.7)
Smoking, n (%)	
Current	2 (5.1%)
Past	5 (12.8%)
Never	32 (82.1%)
Disease duration, median years (IQR)	9.0 (4.0; 18.5)
HBI, median kg/m^2^ (IQR)	5.0 (3.0; 7.7)
Biologic treatment, n (%)	
Anti-TNF	22 (56.4%)
Ustekinumab	8 (20.5%)
Vedolizumab	9 (23.1%)
CD Montreal location, n (%)	
Ileum (L1)	19 (48.7%)
Colon (L2)	4 (10.2%)
Ileocolonic (L3)	16 (41.1%)
CD Montreal behavior, n (%)	
Inflammatory (B1)	19 (48.7%)
Stricturing (B2)	12 (30.7%)
Penetrating (B3)	8 (20.6%)
CD perianal disease, n (%)	8 (20.6%)
CD-related surgery, n (%)	8 (20.6%)
Fecal calprotectin, median (μg/g) (IQR)	369.0 (222.5; 724.5)

Categorical variables are presented as counts and their percentages. Continuous variables are presented as median and IQR.

BMI, body mass index; CD, Crohn's disease; HBI, Harvey-Bradshaw Index; TNF, tumor necrosis factor.

### Response to biologic treatment was associated with consistent decreased antibody response to bacterial flagellins

Serum samples for PhIP-Seq analysis were available for n = 30 (76.9%) patients at week 14, n = 25 (64.1%) at week 22, and n = 14 (35.9%) at week 52. Remission rates at weeks 14, 22, and 52 were 50%, 52%, and 57%, respectively. Among all antigen libraries, only those directed against bacterial flagellins showed consistent decreased antibody response at all time points in remission versus nonremission (q < 0.05) (Figure 1a; Supplementary Figure 1A–C [see the Supplementary Digital Content, http://links.lww.com/CTG/B501).

**Figure 1.**
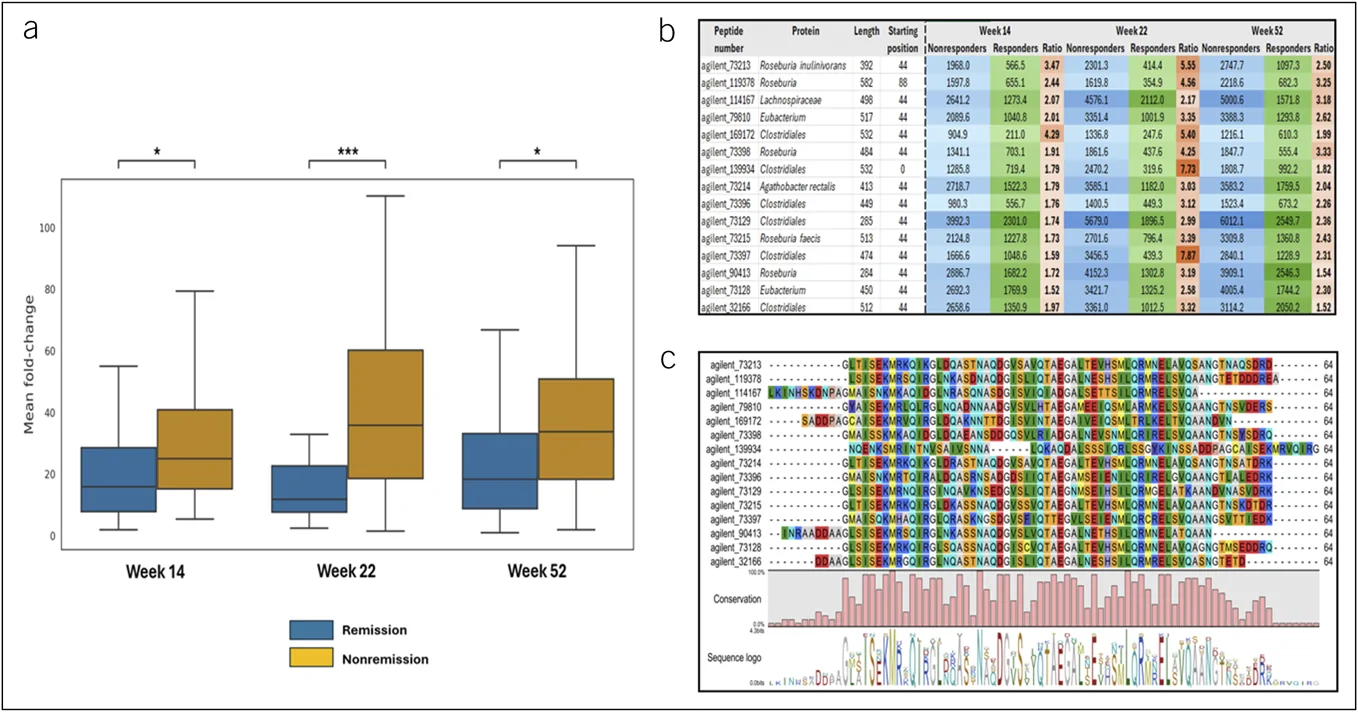
Antibody response against bacterial flagellins is consistently decreased in patients with Crohn's disease achieving remission on biologic treatment and share distinct sequence motifs. (**a**) Box plots showing the mean fold change of each antiflagella oligo at each time point comparing patients achieving remission (blue) versus patients not achieving remission (yellow) after commencing biologic treatment. Adjusted *P* values are calculated using the 2-sided Mann-Whitney *U* test with Benjamini-Hochberg correction. *0.01 < *P* < 0.05, **0.001 < *P* < 0.01, ***0.0001 < *P* < 0.001. (**b**) Information on the 15 antibody-bound peptides from bacterial flagellins that were consistently >1.5-fold overrepresented in nonremission versus remission at weeks 14, 22, and 52. The table shows the library peptide ID, original protein, peptide length, starting position of the peptide, peptide frequencies in patients with Crohn's disease achieving or not-achieving remission, and their ratios. Details on all other flagellin bound peptides are provided in Supplementary Table 1 (see the Supplementary Digital Content, http://links.lww.com/CTG/B502). (**c**) Full-sequence alignments of the 15 flagellin peptides demonstrating consistent > 1.5-fold overrepresentation in nonremission shows shared conserved amino acid sequence motifs. Sequence alignments were generated using Molecular Evolutionary Genetics Analysis (MEGA) software (using the multiple sequence comparison by log-expectation algorithm in default settings, see Structured Transparent Accessible Reporting [STAR] Methods). Visualization was performed using CLC Main Workbench (QIAGEN CLC bioinformatics software suite) version 6.

Fifteen of 55 antiflagellins showed consistent > 1.5-fold overrepresentation among nonremission compared with remission samples. Interestingly, these antibodies showed high conservation of their amino acids sequences, and most were directed against bacterial taxa belonging to either order *Clostridiales*, family *Lachnospiraceae* or *Roseburia* species (Figure 1b,c; Supplementary Table 1 [see the Supplementary Digital Content, http://links.lww.com/CTG/B502]).

Sensitivity analysis using a more relaxed threshold (5%–95% prevalence) yielded consistent results for the flagellin antibody associations (data not shown).

### Response to biologic treatment was associated with specific compositional microbial alterations compared with baseline

Paired fecal samples were available for n = 30 (76.9%) patients at baseline and week 14, n = 25 (64.1%) at week 22, and n = 14 (35.9%) at week 52. Remission rates at weeks 14, 22, and 52 were 50%, 52%, and 57%, respectively.

Patients achieving remission showed a consistent increase in alpha diversity and beta diversity shifts, whereas no change was observed in patients not achieving remission (Figure 2a,b shows the results for week 52; results for weeks 14 and 22 are shown in Supplementary Figure 2A,B and Supplementary Figure 3A,B [see the Supplementary Digital Content, http://links.lww.com/CTG/B501], respectively).

**Figure 2.**
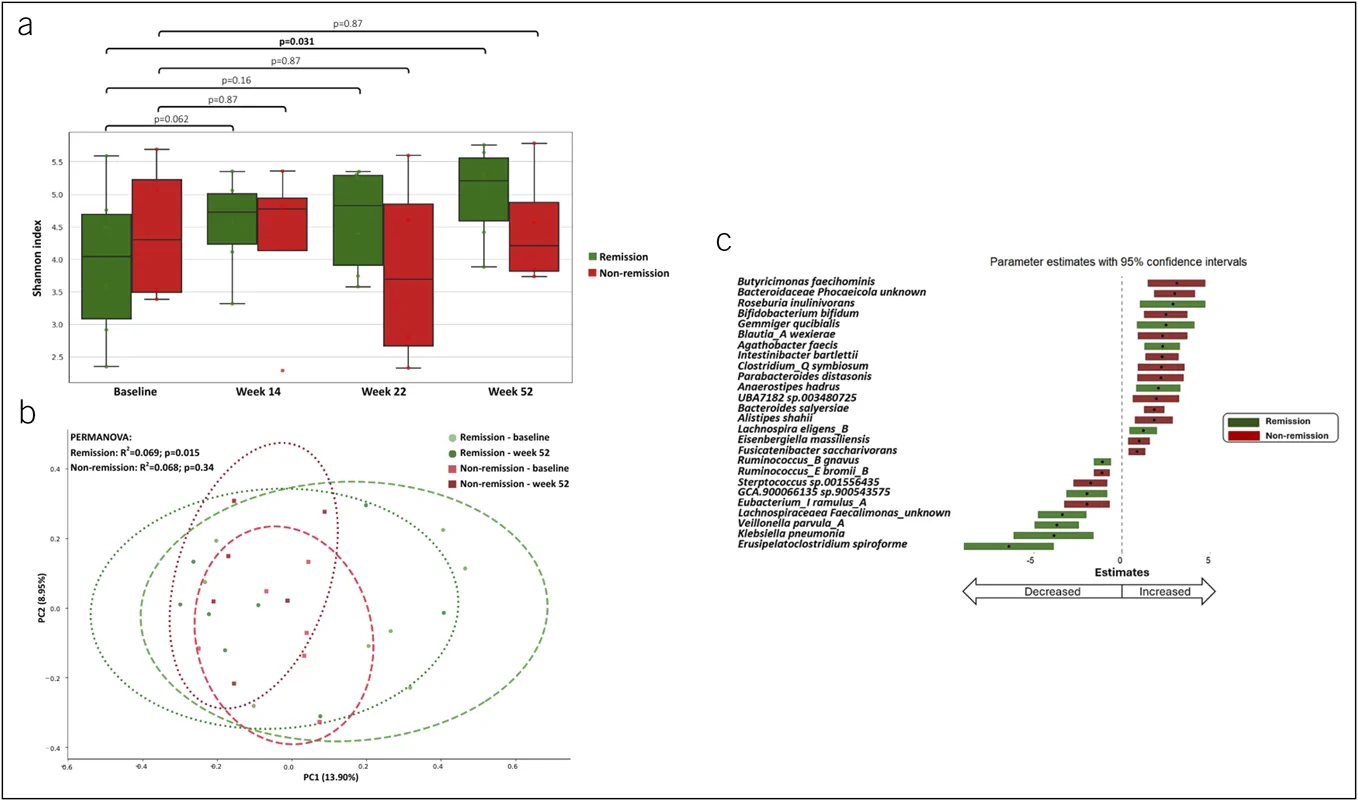
Microbial diversity and individual species abundance alterations in patients achieving remission and not achieving remission on biologic treatment at week 52. (**a**) Alpha diversity expressed by the Shannon index based on remission at week 52 (analysis was performed on the paired samples available at all time points; remission [green], n = 6 vs nonremission [red], n = 4). *P* value is calculated using the Wilcoxon signed-rank sum test. (**b**) Beta diversity assessed with principal coordinates analysis plots at the species level based on the Bray-Curtis dissimilarity index. Comparisons were analyzed within each group on the paired samples—remission (n = 8) at week 52 (green squares) compared baseline (green dots); nonremission (n = 6) at week 52 (red squares) compared with baseline (red dots). Ellipsoids represent a 95% CI. PERMANOVA was applied on distance matrices with 1,000 permutations with patient ID defined as paired strata. Significant shifts from baseline were noted only in patients achieving remission at week 52. (**c**) Coefficient plot showing the effect size and 95% CI (x-axis) for the species found to be significantly differentially abundant (q-values < 0.05) after treatment initiation at week 52 in patients achieving remission (n = 8) (blue) and nonremission (n = 6) (yellow) (y-axis), based on multivariate analysis by linear models adjusted for age, sex, and body mass index. CI, confidence interval; PERMANOVA, permutation analysis of variance.

At the species level, the abundance of 11, 16, and 26 species significantly changed at weeks 14, 22, and 52, respectively. Among the differentially abundant taxa, remission was associated with increased abundance of 2 species belonging to *Roseburia* genera, i.e., *Roseburia intestinalis* (beta-coefficient 2.4, 95% confidence interval [CI] 0.92–3.88, q = 0.021) and *Roseburia inulinivorans* (beta-coefficient 2.88, 95% CI 1.03–4.74, q = 0.036), as well as in the short chain fatty acid producer *Enterocloster sp001517625* (beta-coefficient 2.04, 95% CI 0.92–3.17, q = 0.006) and with the decreased abundance of 2 species belonging to *Ruminococcus* genera, i.e., *Ruminococcus_A sp003011855* (beta-coefficient −1.55, 95% CI −2.19 to −0.91, q = 8.6e-5) and *Ruminococcus_B gnavus* (beta-coefficient −1.1, 95% CI −1.59 to −0.61, q = 3e-4), all of the above belonging to the *Lachnospiraceae* family. Nonremission was associated with the increased abundance of species belonging to the *Bacteroidales* order, including *Bacteroides salyersiae* (beta-coefficient 1.83, 95% CI 1.24–2.41, q = 1.24e-7), *Parabacteroides distasonis* (beta-coefficient 2.19, 95% CI 0.88–3.51, q = 0.016), and *Butyricimonas faecihominis* (beta-coefficient 3.11, 95% CI 1.46–4.74, q = 0.006), and with decreased abundance of *Dorea formicigenerans* (beta-coefficient −1.24, 95% CI −1.98 to −0.49, q = 0.024) (Figure 2c shows the results for week 52; results for weeks 14 and 22 are shown in Supplementary Figures 2C and 3C [see the Supplementary Digital Content, http://links.lww.com/CTG/B501], respectively; see Supplementary Tables 2–4, Supplementary Digital Content, http://links.lww.com/CTG/B503, http://links.lww.com/CTG/B504, http://links.lww.com/CTG/B505). Species with consistent direction of effect in remission and nonremission at all time points (with at least 1 time point with q < 0.05) are shown in Supplementary Figure 4A,B (see the Supplementary Digital Content, http://links.lww.com/CTG/B501).

Interestingly, sensitivity analysis including only flagellated taxa showed no differences between groups (see Supplementary Figure 5, Supplementary Digital Content, http://links.lww.com/CTG/B501).

### Response to biologic treatment is associated with alterations in microbial metabolic pathways involved in cellular oxidative stress and purine biosynthesis

Next, we assessed the microbial functional capacity alterations associated with response to biologic treatment. Remission was associated with changes in 4 pathways, whereas nonremission with changes in 19 pathways (q < 0.05 for all). No significant associations were found at week 22. Remission showed depletion in purine nucleotide degradation (PWY6353; beta-coefficient −0.56, 95% CI −0.88 to −0.23, q = 0.041) and pyruvate fermentation (PWY7111; beta-coefficient −0.38, 95% CI −0.59 to −0.16, q = 0.039), whereas nonremission was associated with depletion in 5-aminoimidazole ribonucleotide biosynthesis (PWY6122; beta-coefficient −0.19, 95% CI −0.31 to −0.06, q = 0.034), pentose phosphate pathway (PWY8178; beta-coefficient −0.23, 95% CI −0.36 to −0.09, q = 0.027), arginine biosynthesis (PWY7400; beta-coefficient −0.28, 95% CI −0.41 to −0.16, q = 8.8e-4), and ornithine biosynthesis (GLUTORN-PWY; beta-coefficient −0.40, 95% CI −0.61 to −0.19, q = 0.012), and with an increase in histidine degradation (HISDEG-PWY; beta-coefficient 1.15, 95% CI 0.75–1.55, q = 6.1e-7) (see Supplementary Tables 5–7, Supplementary Digital Content, http://links.lww.com/CTG/B508). Microbial pathways with consistent direction of effect in remission and nonremission at all time points (with at least 1 time point with q < 0.05) are shown in Supplementary Figure 6A,B (see the Supplementary Digital Content, http://links.lww.com/CTG/B501).

### Antibodies against bacterial flagellins and microbial species and pathways associated with response and nonresponse show a modest correlation pattern

Finally, we investigated the relationship between the 15 antiflagellin antibodies showing consistent nonremission to remission ratio > 1.5-fold overrepresentation, and the microbial species and MetaCyc pathways found to be associated with response to biologic treatment. Although few distinctive correlation patterns at the different time points were observed, none remained significant after correction for multiple testing (FDR < 0.05).

In patients achieving remission, an inverse correlation was found with species whose abundance increased, such as *Prevotella copri*, whereas a direct correlation was found with species and pathways whose abundance decreased, including *Ruminococcus_A sp003011855, Erysipelatoclostridium spiroforme*, unknown species belonging to *Faecalimonas genus*, PWY7111—pyruvate fermentation to isobutanol, PWY7328—superpathway of uridine diphosphate (UDP)-glucose-derived O-antigen building blocks biosynthesis, and PWY6353—purine nucleotides degradation (Figure 3a,b).

**Figure 3.**
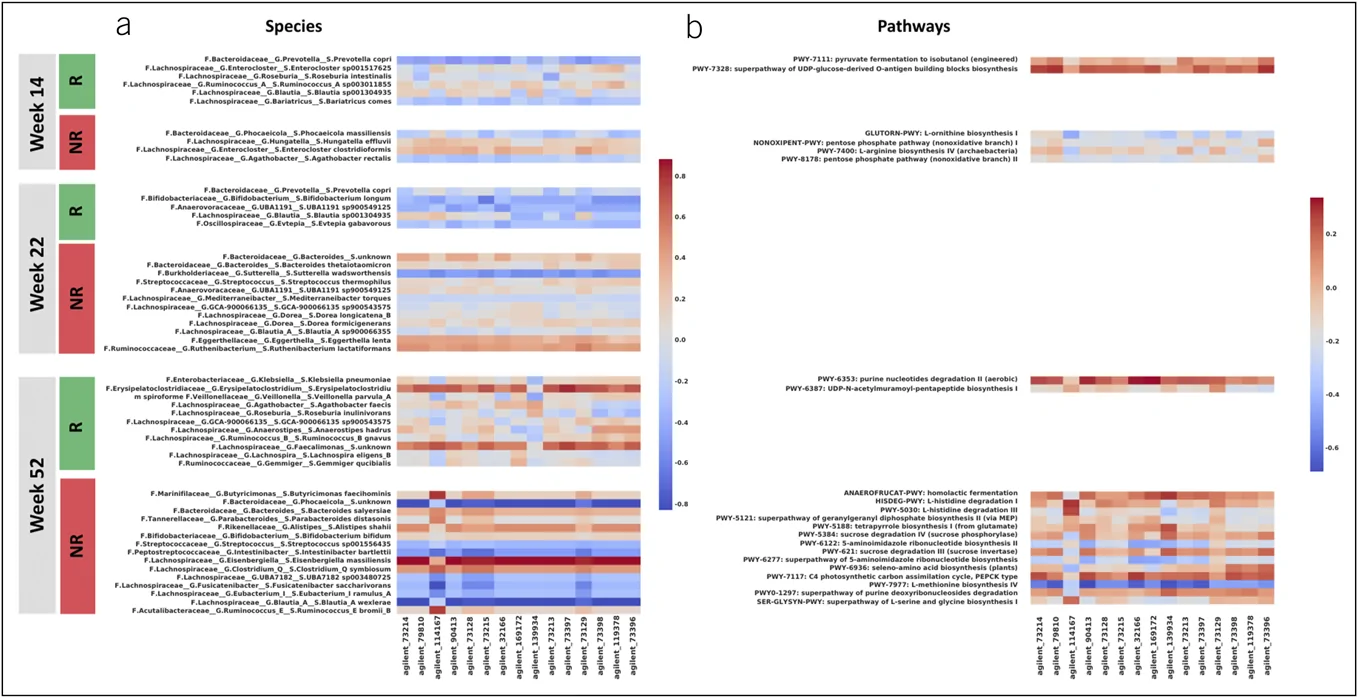
Correlations between serum antibodies against bacterial flagellins and microbial species and pathways associated with remission and nonremission. Heatmap of differentially abundant microbial species (**a**) and MetaCyc pathways (**b**) at weeks 14, 22, and 52 (y-axis) and the 15 antibody-bound peptides from bacterial flagellins that were consistently >1.5-fold overrepresented in nonresponders versus responders (x-axis). As there was no significant association of the microbial MetaCyc pathways with remission status at week 22, correlations were only performed on weeks 14 and 52. Correlations are based on the Spearman rank correlation test. NR, nonremission; R, remission.

The opposite correlation pattern was found in patients not achieving remission, showing a direct correlation with species and pathways that their abundance increased, such as *Hungatella effluvi*, *Eisenbergiella massiliensis*, and HISDEG-PWY: L-histidine degradation, and an inverse correlation with species whose abundance decreased, including *Agathobacter rectalis* (Figure 3a,b).

## DISCUSSION

Despite efforts to facilitate personalized medicine guided by serum and fecal biomarkers to improve outcomes in patients with CD requiring advanced biologic treatments, results are conflicting and not yet incorporated into clinical practice (21,22). In this prospective study, using high-throughput PhIP-Seq, we found that patients with CD who achieved remission on biologic treatment showed attenuated host immune response against bacterial flagellins, remaining consistent at up to 1 year of follow-up. Moreover, we identified a subset of antiflagellin antibodies with consistent overrepresentations in nonresponders vs. responders, showing conserved amino acid structure, suggesting a potential underlying common biologic mechanism. In addition, remission was associated with distinct microbial compositional and functional changes, including taxa related to bacterial flagellins.

Previous studies showed that patients with CD display strong adaptive immune response to human-derived commensal bacterial flagellins (8,9,23). Moreover, serum anti-CBir1, anti-Fla-X, and anti-A4-Fla2 flagellin antibodies are associated with complicated disease course of stricturing and penetrating phenotype (10,24). However, whether host immune response against bacterial flagellins is associated with treatment response among patients with CD commencing biologic treatment is unknown.

Alexander et al showed that patients with CD selectively displayed enhanced serum IgG and peripheral blood effector T-cell responses to human-derived *Lachnospiraceae* flagellins (9). Interestingly, we identified several species belonging to the *Lachnospiraceae* family associated with treatment response. We found that remission was associated with trajectory increased abundance of *Roseburia intestinalis and Roseburia inulinivorans*. *Roseburia intestinalis*, a short chain fatty acid producer, ameliorates inflammation in mouse models. Han et al showed that in a dextran sulfate sodium-induced colitis model, orally administered *Roseburia intestinalis*-derived extracellular vesicles inhibited colonic inflammation and promoted intestinal recovery through downregulating NFκB and STAT3 pathways and increasing *Bifidobacterium* abundance. Shen et al demonstrated that *Roseburia intestinalis* inhibited colonic inflammation in a 2,4,6-trinitrobenzenesulfonic acid-induced colitis mouse model by increasing anti-inflammatory Tregs differentiation through stimulation of thymic stromal lymphopoietin production in intestinal epithelial cells through TLR-5, which is the receptor through which flagellins activate immune and nonimmune cells (6,25). Using samples from patients with CD, they also showed that the expression of thymic stromal lymphopoietin positively correlated with the level of *Roseburia* intestinalis (25), further supporting its anti-inflammatory role.

We also found that after treatment initiation, remission was associated with a consistent decrease in the abundance of *Ruminococcus gnavus* at all time points compared with nonremission. Similarly, Ananthakrishnan et al showed that response to anti-integrin therapy was associated with decreased abundance of *Ruminococcus gnavus* (26). *Ruminococcus gnavus*, a mucin-degrading flagellated bacterium colonizing the intestinal mucosal surface, is enriched in patients with CD and is associated with inflammation and disease flares (27–29). Nooij et al demonstrated *Ruminococcus gnavus* isolates from patients with CD are genomically distinct from healthy controls. Moreover, *Ruminococcus* gnavus motility through flagellum biosynthesis was observed exclusively in newborns and infants up to 1 year of age, suggesting restriction to infant-derived strains (30). Interestingly, Zhao et al identified a dominant epitope in patients with CD, located at the aminoterminus of *Lachnospiraceae* flagellins, recognized by B cells. High levels of serum IgG to that epitope were also found in healthy infants at 1 year of age, which decremented rapidly afterward, suggesting that in contrast to healthy controls, patients with CD may represent failure of a homeostatic response to the gut microbiota beginning in infancy (23). Furthermore, a recent study by Wu et al revealed that in healthy first-degree relatives of patients with CD, increased antibody response toward *Lachnospiraceae* flagellins is associated with future risk of CD (31). Based on our findings of decreased antibody response to bacterial flagellins in patients achieving remission, we may speculate that response to treatment may be associated with restoration of the immune response against bacterial flagellins, although we cannot determine whether this is the cause or the consequence of decreased inflammation.

Several studies showed the association between the gut microbiota and intestinal permeability (32). Lopetuso et al investigated the role of flagellin/TLR-5 in a CD-like ileitis mouse model, showing that elevated TLR-5 expression in ileal epithelium was augmented by gut microbiome presence and that TLR-5 activation in response to bacterial flagellin resulted in increased permeability with altered tight junction protein expression (33). Notably, in a sensitivity analysis including only flagellated taxa, we found no difference in luminal abundance between patients achieving remission vs nonremission. Based on these findings, we may speculate that the difference in host immune response to bacterial flagellins in remission vs nonremission is the result of TLR-5 epithelial expression and alterations in intestinal permeability, rather than microbial composition alone.

When we assessed the association between treatment response and microbial functional capacity, we found greater amount of pathways that were associated with nonremission compared with remission. Patients not achieving remission showed a trajectory decrease in pathways involved in the biosynthesis of arginine and ornithine, semiessential amino acid precursors of nitric oxide and polyamines involved in permeability and gut adaptive responses (34). This association was also demonstrated by Ananthakrishnan et al, further supporting our findings. Decreased arginine has been shown to be associated with active CD (35,36), and mouse studies demonstrated that arginine supplementation may prevent gut mucosal damage and restore intestinal barrier integrity (37,38). Interestingly, 2 species we identified to decrease in patients not achieving remission, i.e., *Agathobacter rectalis* and *Dorea formicigenerans*, contribute to the arginine biosynthesis pathway, potentially driving the decrease in this pathway in nonresponders.

Finally, we investigated for relationships between serum antiflagellin antibodies and the species found to be associated with treatment response; however, no significant correlations were found. This lack of concordance between individuals' serum antibody responses and fecal microbial composition was also noted by Bourgonje et al (8). Although no correlations were statistically significant, few species belonging to the *Lachnospiraceae* family showed a consistent correlative direction with the antiflagellar antibodies. In patients not achieving remission, a direct correlation pattern was found with *Hungatella effluvi* and *Eisenbergiella massiliensis* whose abundance increased, and an inverse correlation pattern with *Agathobacter rectalis* whose abundance decreased post treatment, whereas in patients achieving remission, a direct correlation pattern was found with *Ruminococcus_A sp003011855* whose abundance decreased posttreatment.

Our study has several strengths. First, longitudinal sample collection at different time points allowed consistent signal identification, providing robustness compared with single-timepoint findings. Second, shotgun metagenomic sequencing allowed investigation of compositional and functional microbial flagellin-related species and pathways, providing potential mechanistic insight into their relation to antiflagellar antibodies in responders and nonresponders.

We acknowledge several limitations. First, owing to the relatively small sample size, we could not compute prediction models based on PhIP-Seq and microbiome, focusing on associations alone. Importantly, although few studies have suggested that the gut microbiota composition may be used to predict treatment response (39,40), in a recent study, Prins et al (5) showed marginal predictive capacity of the gut microbiome and fecal metabolites for treatment response compared with clinical features alone and could not confirm previously published microbiome-related predictive signals observed in similar sized cohorts. We believe that our findings provide additional biological insight to the complexity involved in CD pathogenesis and treatment response beyond prediction. Second, the limited sample size and missing data at later time points may have affected our power to detect additional biological signals beyond those we identified and may limit the generalizability of our findings to broader CD populations. Missing data at weeks 14 and 22 were primarily because of logistical challenges related to the COVID-19 pandemic rather than clinical reasons. At week 52, of the 25 missing samples, 7 (28%) were due to treatment termination after lack of response, which is consistent with expected rates of biologic treatment persistence at 1 year (41). Larger, multicenter studies with complete longitudinal sampling are needed to validate our results. Third, owing to technical issues, only 5 subjects had available baseline pretreatment PhIP-Seq data, allowing only cross-sectional analysis at outcome timepoints. This represents a significant limitation because we could not assess how antibody responses change after treatment initiation compared with baseline. Future studies should incorporate longitudinal PhIP-Seq samples to enable insight into antibody response trajectories. Fourth, we cannot claim for causation; associations with host immune response to bacterial flagellins and related species reflect underlying inflammatory status after treatment rather than driving response itself. Fifth, we could not stratify our findings based on the different biologic agents due to limited sample sizes in each treatment group. This represents a limitation as anti-TNF agents, vedolizumab, and ustekinumab have distinct mechanisms of action that may differentially influence immune responses and the gut microbiome. However, a recent review by Chen et al (22) of multiomics biomarkers in inflammatory bowel diseases suggests that microbiome signals associated with treatment response, including increases in short-chain fatty acid-producing bacteria, are consistent across different biologic classes, implying that improvement in inflammatory burden may be a more important driver than specific drug mechanisms. Nevertheless, larger studies stratified by treatment type, including newer biologic and small molecule agents, are needed to determine whether the flagellin antibody signatures we identified are influenced by specific therapeutic mechanisms or reflect a common response pattern associated with successful inflammation control.

In conclusion, we have shown that response to biologic treatment in patients with CD is associated with decreased antibody response to bacterial flagellins and related microbial alterations. These findings suggest that flagellin antibody signatures may warrant further investigation as potential biomarkers for treatment response and may inform future studies exploring flagellin-related microbial interventions in patients with CD.

## CONFLICTS OF INTEREST

**Guarantor of the article:** Haim Leibovitzh, MD. haimlei@tlvmc.gov.il.

**Specific author contributions:** N.M., E.S., H.L., D.K., and Y.S.: conceived and designed the study. N.M.: was responsible for patient recruitment. N.M. and H.L.: managed and monitored data. H.L., D.K., and Y.S. analyzed and reviewed microbial and PhIP-Seq data and interpreted the results. H.L. and N.M.: drafted the report. A.G. and A.W. were responsible for microbial and PhIP-Seq data processing and sequencing. N.M., H.L., N.A.C., A.H., Y.R., and T.T. recruited patients. H.L., D.K., Y.S., N.M., and E.S.: critically reviewed the manuscript. All authors read and approved the final version of the report.

**Financial support:** The project was supported by the Kenneth Rainin Foundation innovator award grant. For more information about the Kenneth Rainin Foundation's work, visit https://krfoundation.org/. Research reported in this publication was supported by the National Heart, Lung, and Blood Institute of the National Institutes of Health under Award Number R01HL160862. The content is solely the responsibility of the authors and does not necessarily represent the official views of the National Institutes of Health; Louis H. Sackin Research Fellow Chair in Computer Science supports a staff scientist in Prof. Segal's Lab.

**Potential competing interests:** N.M. has received grants, speaking and/or consulting fees from Pfizer, Takeda, Janssen, Lilly, Abbvie, Ferring, BiomX, Abbott, BMS, Teva, and Nestle Health Science. H.L., D.K., Y.S., N.A.C., A.H., Y.R., T.T., A.G., A.W., and E.S. declare no competing interests.

**IRB approval statement:** The Tel Aviv Sourasky ethics committee approved the study (IRB# 0162-19-TLV); informed consent was obtained.

**Data accessibility statement:** The data that support the findings of this study are available on request from the corresponding author.

**Study Highlights**

**WHAT IS KNOWN**

✓ Enhanced flagellin immune response associates with aggressive Crohn's disease course.
✓ Relationship between flagellin antibodies and biologic treatment response is unknown.
✓ Current biomarkers for predicting biologic treatment response are lacking.

**WHAT IS NEW HERE**

✓ PhIP-Seq identifies attenuated flagellin antibodies in biologic treatment responders.
✓ Fifteen conserved flagellin antibodies consistently overrepresented in nonresponders.
✓ Remission associates with specific microbiome changes in flagellin-target taxa.
✓ Findings suggest potential biomarkers for predicting biologic treatment response.

## Supplementary Material

## ABBREVIATIONS:

CD: Crohn's disease
CI: confidence interval
FCP: fecal calprotectin
FDR: false discovery rate
PhIP-Seq: phage-display immunoprecipitation sequencing
TLR-5: toll-like receptor-5
TNF: tumor necrosis factor

## References

[1] Baumgart DC Sandborn WJ. Crohn's disease. Lancet 2012;380(9853):1590–605. 10.1016/S0140-6736(12)60026-9 22914295

[2] de Souza HS Fiocchi C. Immunopathogenesis of IBD: Current state of the art. Nat Rev Gastroenterol Hepatol 2016;13(1):13–27. 10.1038/nrgastro.2015.186 26627550

[3] Barberio B Gracie DJ Black CJ . Efficacy of biological therapies and small molecules in induction and maintenance of remission in luminal Crohn's disease: Systematic review and network meta-analysis. Gut 2023;72(2):264–74. 10.1136/gutjnl-2022-328052 35907636

[4] Kayal M Ungaro RC Bader G . Net remission rates with biologic treatment in Crohn's disease: A reappraisal of the clinical trial data. Clin Gastroenterol Hepatol 2023;21(5):1348–50. 10.1016/j.cgh.2022.02.044 35245701

[5] Prins FM Hidding IJ Klaassen MAY . Limited predictive value of the gut microbiome and metabolome for response to biological therapy in inflammatory bowel disease. Gut Microbes 2024;16(1):2391505. 10.1080/19490976.2024.2391505 PMC11340771 39167702

[6] Hajam IA Dar PA Shahnawaz I . Bacterial flagellin—a potent immunomodulatory agent. Exp Mol Med 2017;49(9):e373. 10.1038/emm.2017.172 PMC5628280 28860663

[7] Bourgonje AR Hörstke NV Fehringer M . Systemic antibody responses against gut microbiota flagellins implicate shared and divergent immune reactivity in Crohn's disease and chronic fatigue syndrome. Microbiome 2024;12(1):141. 10.1186/s40168-024-01858-1 PMC11285207 39075559

[8] Bourgonje AR Andreu-Sánchez S Vogl T . Phage-display immunoprecipitation sequencing of the antibody epitope repertoire in inflammatory bowel disease reveals distinct antibody signatures. Immunity 2023;56(6):1393–409.e6. 10.1016/j.immuni.2023.04.017 37164015

[9] Alexander KL Zhao Q Reif M . Human microbiota flagellins drive adaptive immune responses in Crohn's disease. Gastroenterology 2021;161:522–35.e6. 10.1053/j.gastro.2021.03.064 PMC8489510 33844987

[10] Papadakis KA Yang H Ippoliti A . Anti-flagellin (CBir1) phenotypic and genetic Crohn's disease associations. Inflamm Bowel Dis 2007;13(5):524–30. 10.1002/ibd.20106 17260364

[11] Vogl T Klompus S Leviatan S . Population-wide diversity and stability of serum antibody epitope repertoires against human microbiota. Nat Med 2021;27(8):1442–50. 10.1038/s41591-021-01409-3 34282338

[12] Rothschild D Leviatan S Hanemann A . An atlas of robust microbiome associations with phenotypic traits based on large-scale cohorts from two continents. PLoS One 2022;17(3):e0265756. 10.1371/journal.pone.0265756 PMC8947124 35324954

[13] Suez J Korem T Zeevi D . Artificial sweeteners induce glucose intolerance by altering the gut microbiota. Nature 2014;514(7521):181–6. 10.1038/nature13793 25231862

[14] Bolger AM Lohse M Usadel B. Trimmomatic: A flexible trimmer for illumina sequence data. Bioinformatics 2014;30(15):2114–20. 10.1093/bioinformatics/btu170 PMC4103590 24695404

[15] Langmead B Trapnell C Pop M Ultrafast and memory-efficient alignment of short DNA sequences to the human genome. Genome Biol 2009;10(3):R25. 10.1186/gb-2009-10-3-r25 PMC2690996 19261174

[16] Abubucker S Segata N Goll J Metabolic reconstruction for metagenomic data and its application to the human microbiome. PLoS Comput Biol 2012;8(6):e1002358. 10.1371/journal.pcbi.1002358 PMC3374609 22719234

[17] Suzek BE Wang Y Huang H UniRef clusters: A comprehensive and scalable alternative for improving sequence similarity searches. Bioinformatics 2015;31(6):926–32. 10.1093/bioinformatics/btu739 PMC4375400 25398609

[18] Caspi R Billington R Ferrer L The MetaCyc database of metabolic pathways and enzymes and the BioCyc collection of pathway/genome databases. Nucleic Acids Res 2016;44(D1):D471–80. 10.1093/nar/gkv1164 PMC4702838 26527732

[19] Mallick H Rahnavard A McIver LJ Multivariable association discovery in population-scale meta-omics studies. Plos Comput Biol 2021;17(11):e1009442. 10.1371/journal.pcbi.1009442 PMC8714082 34784344

[20] Benjamini Y Hochberg Y. Controlling the false discovery rate: A practical and powerful approach to multiple testing. J R Stat Soc Ser B 1995;57(1):289–300.

[21] Gisbert JP Chaparro M. Predictors of primary response to biologic treatment (anti-TNF, vedolizumab, and ustekinumab) in patients with inflammatory bowel disease: from basic science to clinical practice. J Crohns Colitis 2020;14(5):694–709. 10.1093/ecco-jcc/jjz195 31777929

[22] Chen L Zhang C Niu R, et al. Multi-omics biomarkers for predicting efficacy of biologic and small-molecule therapies in adults with inflammatory bowel disease: A systematic review. United Eur Gastroenterol J. 2025;13(4):517–30. 10.1002/ueg2.12720 PMC12090831 39656426

[23] Zhao Q Duck LW Killian JT Jr Crohn's patients and healthy infants share immunodominant B cell response to commensal flagellin peptide epitopes. Gastroenterology 2024;167(7):1415–28. 10.1053/j.gastro.2024.08.015 PMC11581912 39173722

[24] Targan SR Landers CJ Yang H Antibodies to CBir1 flagellin define a unique response that is associated independently with complicated Crohn's disease. Gastroenterology 2005;128(7):2020–8. 10.1053/j.gastro.2005.03.046 15940634

[25] Shen Z Luo W Tan B, et al. Roseburia intestinalis stimulates TLR5-dependent intestinal immunity against Crohn’s disease. EBioMedicine. 2022;85:104285. 10.1016/j.ebiom.2022.104285 PMC9526137 36182776

[26] Ananthakrishnan AN Luo C Yajnik V Gut microbiome function predicts response to anti-integrin biologic therapy in inflammatory bowel diseases. Cell Host Microbe 2017;21(5):603–10.e3. 10.1016/j.chom.2017.04.010 PMC5705050 28494241

[27] Hall AB Yassour M Sauk J A novel *Ruminococcus gnavus* clade enriched in inflammatory bowel disease patients. Genome Med 2017;9(1):103. 10.1186/s13073-017-0490-5 PMC5704459 29183332

[28] Hassouneh SA Loftus M Yooseph S. Linking inflammatory bowel disease symptoms to changes in the gut microbiome structure and function. Front Microbiol 2021;12:673632. 10.3389/fmicb.2021.673632 PMC8326577 34349736

[29] Moore WC Johnson J Ljijos H, et al. Emendation of bacteroidaceae and butyrivibrio and descriptions of desulfomonas gen. nov. and ten new species in the genera desulfomonas, butyrivibrio, eubacterium, clostridium, and ruminococcus. Int J Syst Bacteriol. 1976;26(2):238–52.

[30] Nooij S Plomp N Sanders IMJG Metagenomic global survey and in-depth genomic analyses of Ruminococcus gnavus reveal differences across host lifestyle and health status. Nat Commun 2025;16(1):1182. 10.1038/s41467-025-56449-x PMC11782615 39885121

[31] Wu RY Xue M Zhao Q, et al. Serum IgG response to a conserved domain of commensal flagellins predicts future risk of Crohn’s disease in first-degree relatives. Clin Gastroenterol Hepatol. 2026. doi:10.1016/j.cgh.2025.12.006. 41534761.

[32] Leibovitzh H Lee SH Xue M Altered gut microbiome composition and function are associated with gut barrier dysfunction in healthy relatives of patients with Crohn's disease. Gastroenterology 2022;163(5):1364–76.e10. 10.1053/j.gastro.2022.07.004 35850197

[33] Lopetuso LR Jia R Wang X-M Epithelial-specific toll-like receptor (TLR)5 activation mediates barrier dysfunction in experimental ileitis. Inflamm Bowel Dis 2017;23(3):392–403. 10.1097/MIB.0000000000001035 28146004

[34] Cynober L. Can arginine and ornithine support gut functions? Gut 1994;35(1 Suppl l):S42–5. 10.1136/gut.35.1_suppl.s42 PMC1378146 8125389

[35] Krzystek-Korpacka M Fleszar MG Bednarz-Misa I Transcriptional and metabolomic analysis of L-arginine/nitric oxide pathway in inflammatory bowel disease and its association with local inflammatory and angiogenic response: Preliminary findings. Int J Mol Sci 2020;21(5):1641. 10.3390/ijms21051641 PMC7084352 32121248

[36] Li J-Y Guo Y-C Zhou H-F Arginine metabolism regulates the pathogenesis of inflammatory bowel disease. Nutr Rev 2023;81(5):578–86. 10.1093/nutrit/nuac070 PMC10086623 36040377

[37] Sukhotnik I Helou H Mogilner J Oral arginine improves intestinal recovery following ischemia-reperfusion injury in rat. Pediatr Surg Int 2005;21(3):191–6. 10.1007/s00383-004-1318-0 15602671

[38] Costa KA Soares ADN Wanner SP L-arginine supplementation prevents increases in intestinal permeability and bacterial translocation in male Swiss mice subjected to physical exercise under environmental heat stress. J Nutr 2014;144(2):218–23. 10.3945/jn.113.183186 24259555

[39] Ventin-Holmberg R Eberl A Saqib S Bacterial and fungal profiles as markers of infliximab drug response in inflammatory bowel disease. J Crohns Colitis 2021;15(6):1019–31. 10.1093/ecco-jcc/jjaa252 33300552

[40] Aden K Rehman A Waschina S Metabolic functions of gut microbes associate with efficacy of tumor necrosis factor antagonists in patients with inflammatory bowel diseases. Gastroenterology 2019;157(5):1279–92.e11. 10.1053/j.gastro.2019.07.025 31326413

[41] Mahlich J May M Feig C Persistence with biologic therapy and associated costs of patients with inflammatory bowel disease: A German retrospective claims data analysis. Crohn's Colitis 360 2021;3(2):otab011. 10.1093/crocol/otab011 PMC9802337 36778945

