# Supplementary material for: Phage-Display Immunoprecipitation Sequencing Reveals Distinct Antibody Signatures Against Bacterial Flagellins Associated With Treatment Response in Crohn's Disease
Source: Clin Transl Gastroenterol. 2026 Apr 2;17(6):e01030. doi: 10.14309/ctg.0000000000001030 (PMC13641668; doi:10.14309/ctg.0000000000001030)
Supplement: Supplementary file 1 [file ct9-17-e01030-s001.pdf]

Supplementary Table 1

Peptide details				Fold change								
				Week 14			Week 22			Week 52		
Peptide number	Protein	Starting position	Length	Non-responders	Responders	Ratio	Non-responders	Responders	Ratio	Non-responders	Responders	Ratio
agilent_167179	*Clostridiales* Flagellin #	220	293	1687.89	193.93	8.70	388.77	1025.94	0.38	1724.33	444.23	3.88
agilent_71860	*Clostridiales Flagellin #*	440	512	1931.60	290.04	6.66	203.38	846.18	0.24	1414.83	473.09	2.99
agilent_164670	*Agathobacter rectalis* Flagellin #	349	413	1409.34	235.94	5.97	187.26	684.93	0.27	1273.35	351.11	3.63
agilent_127282	*Roseburia* Flagellin #	0	462	1058.58	195.90	5.40	1098.11	314.34	3.49	635.15	464.88	1.37
**agilent_169172**	***Clostridiales* Flagellin #**	**44**	**532**	**904.94**	**211.03**	**4.29**	**1336.84**	**247.63**	**5.40**	**1216.12**	**610.30**	**1.99**
**agilent_73213**	***Roseburia inulinivorans* Flagellin #**	**44**	**392**	**1967.98**	**566.48**	**3.47**	**2301.32**	**414.40**	**5.55**	**2747.70**	**1097.35**	**2.50**
agilent_110313	*Bacteria* Flagellin #	521	585	667.30	199.35	3.35	312.93	804.17	0.39	251.12	533.14	0.47
**agilent_119378**	***Roseburia* Flagellin #**	**88**	**582**	**1597.83**	**655.10**	**2.44**	**1619.84**	**354.91**	**4.56**	**2218.64**	**682.29**	**3.25**
agilent_140424	*Enterobacteriaceae* Flagellin #	484	550	538.79	235.41	2.29	150.47	838.03	0.18	364.48	100.32	3.63
agilent_91287	*Eubacterium* Flagellin #	44	531	1828.34	843.92	2.17	2339.46	885.00	2.64	1874.77	1333.97	1.41
agilent_131883	*Clostridiales* Flagellin #	0	285	10435.08	5008.89	2.08	12515.19	5181.39	2.42	10113.57	7194.29	1.41
**agilent_114167**	***Lachnospiraceae* Flagellin #**	**44**	**498**	**2641.23**	**1273.35**	**2.07**	**4576.11**	**2112.05**	**2.17**	**5000.56**	**1571.79**	**3.18**
**agilent_79810**	***Eubacterium* Flagellin #**	**44**	**517**	**2089.63**	**1040.79**	**2.01**	**3351.36**	**1001.86**	**3.35**	**3388.30**	**1293.84**	**2.62**
**agilent_32166**	***Clostridiales* Flagellin #**	**44**	**512**	**2658.59**	**1350.86**	**1.97**	**3361.00**	**1012.51**	**3.32**	**3114.17**	**2050.17**	**1.52**
**agilent_73398**	***Roseburia* Flagellin #**	**44**	**484**	**1341.13**	**703.11**	**1.91**	**1861.58**	**437.58**	**4.25**	**1847.74**	**555.36**	**3.33**
**agilent_139934**	***Clostridiales* Flagellin #**	**0**	**532**	**1285.75**	**719.36**	**1.79**	**2470.22**	**319.61**	**7.73**	**1808.74**	**992.22**	**1.82**
**agilent_73214**	***Agathobacter rectalis* Flagellin #**	**44**	**413**	**2718.70**	**1522.29**	**1.79**	**3585.07**	**1182.02**	**3.03**	**3583.19**	**1759.53**	**2.04**
**agilent_73396**	***Clostridiales* Flagellin #**	**44**	**449**	**980.34**	**556.72**	**1.76**	**1400.49**	**449.26**	**3.12**	**1523.40**	**673.24**	**2.26**
**agilent_73129**	***Clostridiales* Flagellin #**	**44**	**285**	**3992.29**	**2301.03**	**1.74**	**5679.04**	**1896.45**	**2.99**	**6012.09**	**2549.73**	**2.36**
**agilent_73215**	***Roseburia faecis* Flagellin #**	**44**	**513**	**2124.76**	**1227.80**	**1.73**	**2701.62**	**796.42**	**3.39**	**3309.76**	**1360.82**	**2.43**
agilent_43110	*Roseburia* Flagellin #	0	284	4316.79	2498.32	1.73	2428.92	1936.47	1.25	3654.52	2387.53	1.53
**agilent_90413**	***Roseburia* Flagellin #**	**44**	**284**	**2886.74**	**1682.23**	**1.72**	**4152.26**	**1302.78**	**3.19**	**3909.13**	**2546.34**	**1.54**
agilent_126254	*Clostridium sp. AM49-4BH Flagellin #*	0	483	1036.22	642.61	1.61	1250.61	298.87	4.18	1216.01	871.23	1.40
**agilent_73397**	***Clostridiales* Flagellin #**	**44**	**474**	**1666.59**	**1048.64**	**1.59**	**3456.46**	**439.34**	**7.87**	**2840.07**	**1228.88**	**2.31**
agilent_156863	*Enterobacteriaceae* Flagellin #	528	595	544.23	346.35	1.57	203.74	746.38	0.27	197.37	171.90	1.15
agilent_206701	*Roseburia* Flagellin #	44	582	8226.25	5400.30	1.52	9347.27	5377.65	1.74	8905.14	7238.68	1.23
**agilent_73128**	***Eubacterium* Flagellin #**	**44**	**450**	**2692.33**	**1769.92**	**1.52**	**3421.66**	**1325.19**	**2.58**	**4005.43**	**1744.19**	**2.30**
agilent_129819	*Eubacterium sp. CAG:38 Flagellin #*	0	517	3254.99	2170.32	1.50	6598.49	2035.35	3.24	5252.57	3447.88	1.52
agilent_50246	*Clostridiales* Flagellin #	44	491	5370.57	3590.71	1.50	6225.56	2658.08	2.34	5759.18	3908.62	1.47
agilent_131888	*Roseburia inulinivorans* Flagellin #	0	466	4268.80	2854.72	1.50	4335.20	2485.81	1.74	4099.68	2906.98	1.41
agilent_131884	*Roseburia inulinivorans* Flagellin #	0	392	4228.09	2838.40	1.49	4453.48	2434.01	1.83	4409.63	3282.86	1.34
agilent_56259	*Clostridiales* Flagellin #	0	512	5188.05	3506.31	1.48	5277.86	3292.56	1.60	5128.46	3807.56	1.35
agilent_131885	*Eubacterium* Flagellin #	0	450	8111.19	5502.17	1.47	9450.94	4635.60	2.04	8442.87	6358.11	1.33
agilent_128883	*Eubacterium* Flagellin #	0	307	8838.29	6025.13	1.47	10397.74	5551.50	1.87	9414.26	7492.59	1.26
agilent_73393	*Butyrivibrio Flagellin #*	44	452	1438.89	993.21	1.45	4026.97	646.47	6.23	2292.03	525.26	4.36
agilent_131887	*Agathobacter rectalis* Flagellin #	0	413	7935.09	5482.70	1.45	8282.06	4718.64	1.76	8562.52	6686.17	1.28
agilent_73399	*Lachnospiraceae Flagellin #*	44	493	2426.76	1723.77	1.41	3911.49	1133.20	3.45	3513.57	1650.95	2.13
agilent_166348	*Clostridiales* Flagellin #	0	412	3511.60	2542.54	1.38	1446.04	1824.56	0.79	2590.40	1985.71	1.30
agilent_72410	*Unclassified Flagellin #*	44	374	1452.54	1078.55	1.35	2058.34	700.95	2.94	1819.07	890.87	2.04
agilent_73216	*Roseburia inulinivorans* Flagellin #	44	466	4192.14	3118.72	1.34	5825.83	2192.40	2.66	5960.54	2782.35	2.14
agilent_50247	*Clostridiales* Flagellin #	44	412	3390.71	2587.78	1.31	3408.01	2328.29	1.46	3536.37	3359.88	1.05
agilent_72399	*Clostridiales* Flagellin #	44	476	1538.19	1187.75	1.30	2073.21	827.23	2.51	1785.63	947.99	1.88
agilent_72400	*Clostridiales* Flagellin #	44	461	1586.82	1230.57	1.29	1860.64	850.77	2.19	1906.25	1056.93	1.80
agilent_131886	*Roseburia faecis Flagellin #*	0	513	8662.69	6721.72	1.29	11025.62	5711.23	1.93	10268.51	7891.54	1.30
agilent_126605	*Clostridiales* Flagellin #	0	474	2048.10	1591.69	1.29	4487.41	809.86	5.54	2751.90	1833.80	1.50
agilent_73395	*Roseburia* Flagellin #	44	493	1089.90	895.66	1.22	1847.98	460.70	4.01	1524.24	333.85	4.57
agilent_214044	*Clostridiales* Flagellin #	44	487	3290.22	2736.85	1.20	7173.28	2213.74	3.24	5042.13	3581.30	1.41
agilent_130111	*Butyrivibrio Flagellin #*	0	452	3187.22	2746.14	1.16	6558.53	1942.78	3.38	4257.29	2970.03	1.43
agilent_129840	*Roseburia* Flagellin #	0	493	8609.02	7607.87	1.13	14637.50	6844.11	2.14	11575.01	10237.69	1.13
agilent_129842	*Clostridiales* Flagellin #	0	476	1515.29	1400.69	1.08	3740.47	940.32	3.98	2005.53	1957.78	1.02
agilent_126599	*Lachnospiraceae Flagellin #*	0	493	5146.70	4893.37	1.05	8767.73	4588.93	1.91	7323.92	6303.57	1.16
agilent_177201	*Roseburia* Flagellin #	88	574	3774.81	3633.25	1.04	6280.98	3184.84	1.97	6109.42	3706.40	1.65
agilent_129839	*Clostridiales* Flagellin #	0	449	2821.50	2886.84	0.98	6554.82	1964.87	3.34	3870.20	3225.62	1.20
agilent_129843	*Unclassified Flagellin #*	0	374	1815.08	1904.08	0.95	3611.05	1476.12	2.45	2618.11	1874.33	1.40
agilent_126598	*Clostridiales* Flagellin #	0	461	2499.06	2869.59	0.87	5298.05	2098.93	2.52	3376.66	2762.72	1.22
