# Supplementary material for: Phage-Display Immunoprecipitation Sequencing Reveals Distinct Antibody Signatures Against Bacterial Flagellins Associated With Treatment Response in Crohn's Disease
Source: Clin Transl Gastroenterol. 2026 Apr 2;17(6):e01030. doi: 10.14309/ctg.0000000000001030 (PMC13641668; doi:10.14309/ctg.0000000000001030)
Supplement: Supplementary file 2 [file ct9-17-e01030-s002.pdf]

Supplementary Table 2

Remission week 14							
**Species**	**ZP (%)**	**Coefficient**	**Std.error**	**Lower 95% CI**	**Higher 95% CI**	**p-Value**	**q-Value**
Blautia.sp001304935	63.3	-1.636	0.275	-2.176	-1.096	2.84E-09	2.10E-07
Ruminococcus_A.sp003011855	60.0	-1.549	0.328	-2.192	-0.907	2.31E-06	8.56E-05
Prevotella.copri	66.7	1.488	0.323	0.856	2.121	3.99E-06	9.85E-05
Enterocloster.sp001517625	63.3	2.045	0.574	0.921	3.169	3.63E-04	0.0067
Roseburia.intestinalis	56.7	2.403	0.753	0.927	3.878	0.0014	0.0209
Bariatricus.comes	66.7	-1.136	0.375	-1.871	-0.401	0.0024	0.0258
UMGS1071.sp900542375	56.7	-1.127	0.370	-1.852	-0.402	0.0023	0.0258
Streptococcus.salivarius	30.0	-1.611	0.596	-2.778	-0.443	0.0068	0.0633
Oscillibacter.welbionis	53.3	1.841	0.695	0.478	3.203	0.0081	0.0668
Blautia_A.obeum	66.7	-0.607	0.233	-1.065	-0.150	0.0092	0.0684
Streptococcus.sp001556435	46.7	-1.510	0.619	-2.722	-0.298	0.0146	0.0985
Parabacteroides.merdae	53.3	0.966	0.421	0.141	1.791	0.0217	0.1337
Blautia_A.sp000433815	60.0	-0.874	0.390	-1.639	-0.109	0.0251	0.1401
f_Oscillospiraceae.g_Oscillibacter.s_unknown	66.7	1.640	0.739	0.191	3.088	0.0265	0.1401
Bacteroides.fragilis	63.3	2.075	0.991	0.133	4.017	0.0362	0.1641
Phascolarctobacterium.faecium	53.3	0.605	0.288	0.040	1.170	0.0357	0.1641
Dorea.formicigenerans	66.7	-0.575	0.277	-1.117	-0.033	0.0377	0.1641
Parabacteroides.distasonis	46.7	1.213	0.624	-0.010	2.437	0.0520	0.2024
Blautia_A.sp900066355	60.0	-0.912	0.465	-1.823	-0.001	0.0497	0.2024
Bacteroides.uniformis	26.7	0.883	0.492	-0.080	1.847	0.0723	0.2676
Bacteroides.thetaiotaomicron	40.0	0.892	0.523	-0.132	1.916	0.0879	0.3087
Enterocloster.bolteae	13.3	-0.723	0.436	-1.576	0.131	0.0972	0.3087
Dialister.invisus	63.3	-1.122	0.683	-2.460	0.217	0.1004	0.3087
Eggerthella.lenta	46.7	0.844	0.518	-0.171	1.859	0.1033	0.3087
Sutterella.wadsworthensis	60.0	1.463	0.901	-0.302	3.228	0.1043	0.3087
Blautia_A.sp900066165	53.3	-0.862	0.538	-1.917	0.193	0.1091	0.3106
CAG.81.sp900066535	63.3	0.800	0.508	-0.195	1.796	0.1149	0.3148
Alistipes.shahii	63.3	0.384	0.256	-0.118	0.887	0.1340	0.3523
Ruminococcus_B.gnavus	16.7	-0.914	0.620	-2.130	0.302	0.1405	0.3523
Agathobaculum.sp900291975	60.0	-0.645	0.440	-1.508	0.218	0.1428	0.3523
Bilophila.wadsworthia	50.0	0.725	0.532	-0.318	1.768	0.1728	0.4126
Escherichia.flexneri	10.0	-0.722	0.542	-1.784	0.340	0.1828	0.4228
Streptococcus.vestibularis	63.3	-0.918	0.704	-2.298	0.462	0.1922	0.4310
Bacteroides.ovatus	53.3	1.054	0.826	-0.564	2.673	0.2015	0.4387
Alistipes.finegoldii	63.3	-0.286	0.247	-0.770	0.198	0.2465	0.5089
Anaerostipes.hadrus	63.3	0.575	0.497	-0.399	1.549	0.2476	0.5089
Enterocloster.lavalensis	63.3	-0.547	0.509	-1.543	0.450	0.2825	0.5615
Enterocloster.aldenensis	30.0	-0.436	0.411	-1.242	0.369	0.2883	0.5615
Klebsiella.pneumoniae	66.7	-1.254	1.211	-3.628	1.121	0.3008	0.5626
Agathobaculum.butyriciproducens	60.0	-0.563	0.548	-1.636	0.511	0.3041	0.5626
f_Bacteroidaceae.g_Phocaeicola.s_unknown	46.7	0.397	0.413	-0.413	1.206	0.3368	0.6079
Anaerostipes.caccae	60.0	0.665	0.773	-0.850	2.181	0.3895	0.6703
Mediterraneibacter.lactaris	66.7	0.629	0.747	-0.835	2.093	0.3995	0.6703
Streptococcus.thermophilus	30.0	-0.594	0.711	-1.986	0.799	0.4034	0.6703
Alistipes.putredinis	63.3	0.317	0.393	-0.453	1.087	0.4202	0.6703
Blautia_A.wexlerae	60.0	0.435	0.546	-0.635	1.505	0.4257	0.6703
Roseburia.inulinivorans	66.7	0.631	0.788	-0.914	2.176	0.4237	0.6703
Fusicatenibacter.saccharivorans	50.0	-0.377	0.500	-1.357	0.603	0.4510	0.6954
Enterocloster.clostridioformis	36.7	0.459	0.710	-0.933	1.851	0.5181	0.7824
Lactococcus.lactis	60.0	-0.572	0.927	-2.389	1.244	0.5369	0.7947
Phocaeicola.vulgatus	16.7	0.369	0.679	-0.961	1.700	0.5865	0.8455
Clostridium_Q.symbiosum	40.0	-0.322	0.604	-1.506	0.862	0.5942	0.8455
Alistipes.onderdonkii	53.3	-0.392	0.854	-2.066	1.282	0.6464	0.9023
Anaerobutyricum.hallii	53.3	-0.260	0.589	-1.415	0.894	0.6585	0.9023
Agathobacter.rectalis	63.3	0.264	0.622	-0.955	1.483	0.6710	0.9029
Bifidobacterium.longum	40.0	-0.122	0.555	-1.210	0.966	0.8258	0.9483
Bacteroides.cellulosilyticus	66.7	0.228	1.094	-1.916	2.373	0.8346	0.9483
Acetatifactor.sp900066565	66.7	0.141	0.936	-1.694	1.976	0.8803	0.9483
Agathobacter.faecis	63.3	0.219	0.991	-1.722	2.161	0.8248	0.9483
f_Lachnospiraceae.g_Faecalimonas.s_unknown	63.3	-0.143	1.063	-2.226	1.940	0.8929	0.9483
Hungatella.effluvii	53.3	0.213	0.650	-1.061	1.487	0.7433	0.9483

Mediterraneibacter.torques	60.0	0.082	0.544	-0.983	1.148	0.8796	0.9483
CAG.41.sp900066215	60.0	0.094	0.674	-1.228	1.416	0.8893	0.9483
Flavonifractor.plautii	10.0	-0.122	0.304	-0.717	0.474	0.6889	0.9103
Faecalibacterium.prausnitzii_D	63.3	0.245	0.816	-1.354	1.844	0.7642	0.9483
Ruthenibacterium.lactatiformans	56.7	0.124	0.940	-1.718	1.966	0.8949	0.9483
Intestinibacter.bartlettii	36.7	-0.122	0.567	-1.234	0.991	0.8303	0.9483
Erysipelatoclostridium.ramosum	43.3	-0.088	0.597	-1.258	1.082	0.8825	0.9483
Veillonella.parvula_A	50.0	-0.159	0.834	-1.793	1.476	0.8492	0.9483
Anaerotruncus.colihominis	63.3	-0.100	0.924	-1.911	1.711	0.9135	0.9483
Faecalimonas.umbilicata	66.7	-0.045	0.516	-1.055	0.966	0.9311	0.9483
Longicatena.innocuum	26.7	0.042	0.482	-0.903	0.986	0.9311	0.9483
Blautia_A.massiliensis	66.7	0.057	0.885	-1.678	1.793	0.9483	0.9483
Enterocloster.sp900541315	46.7	0.037	0.510	-0.962	1.036	0.9423	0.9483

Non-remission week 14

Species	ZP (%)	Coefficient	Std.error	Lower 95% CI	Higher 95% CI	p-Value	q-Value
Phocaeicola.massiliensis	63.3	1.141	0.291	0.570	1.713	8.98E-05	0.0112
Enterocloster.clostridioformis	40.0	-0.466	0.129	-0.719	-0.214	2.88E-04	0.0180
Hungatella.effluvii	46.7	1.865	0.550	0.786	2.943	7.02E-04	0.0292
Agathobacter.rectalis	43.3	-1.475	0.462	-2.381	-0.570	0.0014	0.0438
Longicatena.innocuum	30.0	0.914	0.298	0.329	1.498	0.0022	0.0546
Anaerobutyricum.hallii_A	56.7	-1.185	0.409	-1.987	-0.384	0.0038	0.0782
Ruminococcus_H.sp003531055	63.3	-1.384	0.489	-2.343	-0.426	0.0046	0.0826
Blautia_A.wexlerae	66.7	-1.646	0.645	-2.910	-0.382	0.0107	0.1673
Parabacteroides.distasonis	36.7	1.361	0.549	0.285	2.436	0.0132	0.1703
Blautia_A.sp000285855	66.7	-1.583	0.645	-2.847	-0.319	0.0141	0.1703
Duodenibacillus.sp900538905	66.7	-0.192	0.079	-0.346	-0.037	0.0150	0.1703
Eggerthella.lenta	56.7	1.518	0.632	0.279	2.757	0.0164	0.1703
Odoribacter.splanchnicus	46.7	0.873	0.375	0.137	1.608	0.0200	0.1822
CAG.103.sp000432375	56.7	-1.202	0.518	-2.218	-0.186	0.0204	0.1822
Clostridium_Q.symbiosum	43.3	0.997	0.435	0.144	1.849	0.0219	0.1824
Faecalibacterium.prausnitzii_G	56.7	-1.220	0.548	-2.294	-0.146	0.0260	0.2031
f_Lachnospiraceae.g_Blautia_A.s_unknown	66.7	-0.960	0.442	-1.827	-0.094	0.0299	0.2073
Clostridium_A.leptum	63.3	-1.488	0.683	-2.826	-0.150	0.0292	0.2073
Lawsonibacter.sp900066825	53.3	-0.706	0.344	-1.381	-0.031	0.0402	0.2647
Bilophila.wadsworthia	56.7	1.145	0.572	0.023	2.266	0.0455	0.2670
Akkermansia.muciniphila_B	63.3	-1.107	0.552	-2.188	-0.025	0.0449	0.2670
Acetatifactor.sp900066365	63.3	-1.111	0.565	-2.220	-0.003	0.0494	0.2670
Escherichia.flexneri	10.0	-1.077	0.546	-2.148	-0.006	0.0488	0.2670
f_Oscillospiraceae.g_Oscillibacter.s_unknown	63.3	1.434	0.735	-0.008	2.875	0.0513	0.2670
Bacteroides.cellulosilyticus	40.0	1.342	0.722	-0.073	2.758	0.0631	0.2816
Parabacteroides.merdae	46.7	0.868	0.460	-0.033	1.770	0.0590	0.2804
Roseburia.hominis	40.0	-0.742	0.391	-1.509	0.024	0.0577	0.2804
CAG.41.sp900066215	40.0	-1.044	0.556	-2.134	0.046	0.0606	0.2804
Oscillibacter.welbionis	43.3	1.049	0.586	-0.099	2.196	0.0733	0.3161
Ruminococcus_D.bicirculans	63.3	-1.158	0.672	-2.476	0.160	0.0851	0.3547
Alistipes.finegoldii	56.7	1.001	0.611	-0.197	2.198	0.1015	0.4010
Hungatella.sp005845265	66.7	1.202	0.737	-0.241	2.646	0.1026	0.4010
Butyricimonas.faecihominis	63.3	1.160	0.718	-0.248	2.568	0.1063	0.4026
Alistipes_A.ihumii	60.0	-0.633	0.413	-1.443	0.176	0.1253	0.4605
Alistipes.onderdonkii	26.7	-0.694	0.466	-1.608	0.220	0.1367	0.4617
Roseburia.inulinivorans	53.3	-0.817	0.555	-1.905	0.271	0.1412	0.4617
Evtepia.gabavorous	63.3	-0.975	0.658	-2.265	0.315	0.1384	0.4617
Bacteroides.caccae	66.7	0.586	0.402	-0.202	1.374	0.1447	0.4617
UMGS1071.sp900542375	50.0	-0.569	0.391	-1.336	0.198	0.1459	0.4617
CAG.81.sp900066535	63.3	0.817	0.580	-0.319	1.953	0.1588	0.4617
CAG.83.sp000431575	66.7	1.458	1.026	-0.553	3.469	0.1554	0.4617
Oscillibacter.sp001916835	60.0	-0.564	0.393	-1.335	0.206	0.1512	0.4617
Streptococcus.sp001556435	63.3	-1.035	0.733	-2.473	0.402	0.1579	0.4617
f_Bacteroidaceae.g_Bacteroides.s_unknown	63.3	0.842	0.616	-0.366	2.049	0.1719	0.4750
Alistipes_A.indistinctus	60.0	-0.419	0.320	-1.047	0.209	0.1912	0.4779
Acetatifactor.sp900066565	56.7	-0.894	0.659	-2.187	0.398	0.1748	0.4750
CAG.81.sp900066785	63.3	0.657	0.480	-0.284	1.597	0.1713	0.4750
Anaerotruncus.colihominis	60.0	0.813	0.622	-0.405	2.032	0.1908	0.4779

Intestinibacter.bartlettii	53.3	0.921	0.699	-0.449	2.291	0.1877	0.4779
Streptococcus.salivarius	56.7	-0.984	0.753	-2.460	0.491	0.1911	0.4779
Enterocloster.aldenensis	40.0	0.439	0.340	-0.226	1.105	0.1959	0.4802
Eisenbergiella.sp900066775	56.7	-0.508	0.401	-1.293	0.278	0.2053	0.4935
Anaerobutyricum.hallii	53.3	0.601	0.485	-0.349	1.552	0.2148	0.5029
Bariatricus.comes	60.0	-0.680	0.561	-1.780	0.419	0.2253	0.5029
UMGS1375.sp900066615	66.7	-0.544	0.446	-1.417	0.329	0.2222	0.5029
Ruminococcus_E.bromii_B	56.7	-0.982	0.796	-2.543	0.580	0.2178	0.5029
f_Lachnospiraceae.g_UBA7160.s_unknown	63.3	-0.500	0.417	-1.316	0.317	0.2302	0.5049
Blautia_A.sp900066165	30.0	-0.445	0.380	-1.190	0.300	0.2420	0.5138
Enterocloster.bolteae	30.0	-0.437	0.376	-1.175	0.301	0.2455	0.5138
f_Lachnospiraceae.g_Faecalimonas.s_unknown	63.3	1.030	0.889	-0.712	2.772	0.2466	0.5138
Lachnospira.sp000437735	60.0	-0.898	0.784	-2.434	0.638	0.2519	0.5160
Akkermansia.muciniphila	56.7	0.863	0.763	-0.633	2.358	0.2581	0.5160
Barnesiella.intestinihominis	66.7	0.787	0.699	-0.583	2.158	0.2601	0.5160
Alistipes.putredinis	53.3	0.740	0.695	-0.622	2.102	0.2867	0.5368
Anaerotignum.sp000436415	66.7	-0.549	0.512	-1.553	0.455	0.2836	0.5368
Dorea.sp000509125	66.7	0.541	0.499	-0.437	1.519	0.2782	0.5368
Prevotella.copri	66.7	0.994	0.938	-0.845	2.832	0.2895	0.5368
Romboutsia.timonensis	60.0	0.422	0.400	-0.363	1.206	0.2920	0.5368
f_Ruminococcaceae.g_Faecalibacterium.s_unknown	63.3	-0.466	0.452	-1.352	0.420	0.3024	0.5477
Bacteroides.stercoris	56.7	0.702	0.692	-0.654	2.058	0.3103	0.5541
CAG.45.sp900066395	56.7	-0.984	0.990	-2.925	0.957	0.3205	0.5642
Erysipelatoclostridium.ramosum	56.7	0.739	0.779	-0.787	2.265	0.3427	0.5950
Fusicatenibacter.saccharivorans	33.3	-0.269	0.295	-0.847	0.310	0.3627	0.6210
Faecalimonas.umbilicata	40.0	-0.490	0.545	-1.559	0.578	0.3684	0.6223
Enterocloster.sp900541315	63.3	-0.414	0.482	-1.358	0.530	0.3902	0.6503
f_Bacteroidaceae.g_Phocaeicola.s_unknown	53.3	-0.227	0.273	-0.763	0.309	0.4065	0.6599
GCA.900066135.sp900543575	66.7	0.537	0.646	-0.729	1.803	0.4055	0.6599
f_Lachnospiraceae.g_Mediterraneibacter.s_unknown	66.7	0.762	0.933	-1.068	2.591	0.4145	0.6642
Faecalibacterium.prausnitzii_C	60.0	0.576	0.726	-0.848	2.000	0.4279	0.6770
Bifidobacterium.longum	46.7	-0.336	0.436	-1.190	0.518	0.4406	0.6883
Hungatella_A.sp003478355	66.7	0.500	0.660	-0.793	1.793	0.4485	0.6883
Faecalibacterium.prausnitzii_A	63.3	-0.558	0.751	-2.030	0.913	0.4570	0.6883
Ruthenibacterium.lactatiformans	33.3	0.524	0.698	-0.844	1.893	0.4527	0.6883
Faecalibacterium.sp900539945	63.3	0.567	0.788	-0.978	2.112	0.4720	0.7024
Bacteroides.thetaiotaomicron	33.3	0.263	0.372	-0.466	0.992	0.4792	0.7047
Phocaeicola.vulgatus	16.7	0.249	0.380	-0.495	0.993	0.5118	0.7379
Anaerostipes.hadrus	23.3	-0.256	0.392	-1.024	0.512	0.5136	0.7379
Bacteroides.ovatus	30.0	-0.256	0.414	-1.067	0.555	0.5365	0.7596
Bacteroides.uniformis	26.7	0.212	0.358	-0.489	0.913	0.5530	0.7596
UMGS1670.sp900548595	66.7	-0.297	0.492	-1.262	0.668	0.5463	0.7596
Lachnospira.eligens_B	60.0	0.408	0.726	-1.015	1.831	0.5742	0.7801
Faecalibacterium.sp003449675	66.7	-0.394	0.658	-1.683	0.895	0.5490	0.7596
Blautia_A.obeum	43.3	0.309	0.570	-0.808	1.425	0.5881	0.7867
Mediterraneibacter.lactaris	56.7	0.342	0.638	-0.908	1.593	0.5916	0.7867
Coprococcus_A.catus	63.3	0.294	0.566	-0.814	1.403	0.6028	0.7931
Erysipelatoclostridium.sp000752095	56.7	-0.235	0.482	-1.180	0.710	0.6256	0.8068
Alistipes.obesi	56.7	0.299	0.645	-0.966	1.563	0.6435	0.8068
Alistipes.shahii	60.0	-0.253	0.636	-1.500	0.994	0.6907	0.8262
Dorea.longicatena	43.3	0.214	0.475	-0.717	1.145	0.6525	0.8076
Enterocloster.citroniae	50.0	0.160	0.407	-0.638	0.958	0.6940	0.8262
KLE1615.sp900066985	63.3	0.318	0.682	-1.018	1.655	0.6406	0.8068
Roseburia.intestinalis	46.7	0.272	0.669	-1.039	1.583	0.6844	0.8262
UBA11774.sp003507655	66.7	-0.340	0.731	-1.773	1.094	0.6426	0.8068
Lawsonibacter.asaccharolyticus	43.3	0.191	0.461	-0.713	1.095	0.6785	0.8262
Gemmiger.qucibialis	53.3	0.204	0.444	-0.665	1.074	0.6454	0.8068
Negativibacillus.massiliensis	56.7	0.210	0.552	-0.872	1.291	0.7038	0.8300
Bacteroides.fragilis	46.7	0.171	0.499	-0.808	1.149	0.7323	0.8555
Bacteroides.xylanisolvens	60.0	-0.171	0.590	-1.327	0.985	0.7719	0.8845
Blautia_A.massiliensis	40.0	0.144	0.489	-0.815	1.102	0.7688	0.8845
Agathobaculum.butyriciproducens	43.3	-0.073	0.318	-0.696	0.550	0.8173	0.9204
ER4.sp000765235	66.7	0.151	0.538	-0.903	1.206	0.7784	0.8845
Ruminococcus_B.gnavus	26.7	-0.104	0.523	-1.128	0.920	0.8421	0.9233
Flavonifractor.plautii	13.3	-0.070	0.323	-0.704	0.564	0.8283	0.9233

Parasutterella.excrementihominis	53.3	0.162	0.798	-1.402	1.725	0.8394	0.9233
Dorea.formicigenerans	46.7	-0.076	0.413	-0.886	0.734	0.8548	0.9291
Clostridium_Q.sp003024715	36.7	-0.030	0.266	-0.552	0.492	0.9096	0.9680
Faecalibacterium.prausnitzii_D	56.7	-0.104	0.844	-1.758	1.551	0.9023	0.9680
Allisonella.histaminiformans	63.3	-0.040	0.366	-0.757	0.678	0.9138	0.9680
Agathobacter.faecis	43.3	-0.050	0.623	-1.272	1.171	0.9355	0.9745
Dorea.sp900066555	60.0	0.048	0.566	-1.061	1.157	0.9321	0.9745
Mediterraneibacter.torques	26.7	0.019	0.366	-0.698	0.735	0.9595	0.9912
Gemmiger.formicilis	60.0	0.020	0.743	-1.436	1.476	0.9785	0.9977
Phascolarctobacterium.faecium	63.3	0.019	0.941	-1.825	1.862	0.9842	0.9977
Blautia_A.sp000433815	60.0	0.007	0.700	-1.365	1.380	0.9915	0.9977
Blautia_A.sp900066355	60.0	0.002	0.609	-1.192	1.195	0.9977	0.9977
