# Supplementary material for: Phage-Display Immunoprecipitation Sequencing Reveals Distinct Antibody Signatures Against Bacterial Flagellins Associated With Treatment Response in Crohn's Disease
Source: Clin Transl Gastroenterol. 2026 Apr 2;17(6):e01030. doi: 10.14309/ctg.0000000000001030 (PMC13641668; doi:10.14309/ctg.0000000000001030)
Supplement: Supplementary file 3 [file ct9-17-e01030-s003.pdf]

Supplementary Table 3

Remission week 22

Species	ZP (%)	Coefficient	Std.error	Lower 95% CI	Higher 95% CI	p-Value	q-Value
Evtepia.gabavorous	69.2	-2.778	0.557	-3.870	-1.685	6.23E-07	5.55E-05
Bifidobacterium.longum	42.3	-2.102	0.548	-3.176	-1.027	1.26E-04	0.0029
Prevotella.copri	53.8	1.584	0.414	0.772	2.396	1.31E-04	0.0029
Blautia.sp001304935	53.8	-1.990	0.508	-2.986	-0.995	8.92E-05	0.0029
UBA1191.sp900549125	65.4	-1.217	0.402	-2.005	-0.430	0.0024	0.0435
Dorea.longicatena	65.4	2.289	0.867	0.591	3.988	0.0083	0.1225
Hungatella.effluvii	46.2	-1.242	0.527	-2.274	-0.209	0.0184	0.1933
UMGS1071.sp900542375	61.5	-1.241	0.543	-2.305	-0.177	0.0223	0.1933
Blautia_A.wexlerae	61.5	2.121	0.963	0.234	4.009	0.0276	0.1933
Clostridium_Q.sp003024715	69.2	-0.899	0.400	-1.683	-0.116	0.0245	0.1933
Dorea.formicigenerans	69.2	0.740	0.337	0.080	1.400	0.0281	0.1933
Oscillibacter.welbionis	38.5	1.120	0.499	0.143	2.097	0.0247	0.1933
Romboutsia.timonensis	65.4	1.786	0.814	0.190	3.381	0.0282	0.1933
Roseburia.intestinalis	53.8	1.203	0.570	0.085	2.320	0.0349	0.2078
Streptococcus.salivarius	38.5	-1.327	0.629	-2.561	-0.093	0.0350	0.2078
Odoribacter.splanchnicus	61.5	1.569	0.808	-0.015	3.153	0.0522	0.2629
Enterocloster.lavalensis	57.7	1.297	0.671	-0.018	2.613	0.0533	0.2629
Lachnospira.sp000437735	65.4	2.451	1.271	-0.041	4.943	0.0539	0.2629
Phocaeicola.vulgatus	30.8	1.232	0.645	-0.032	2.496	0.0561	0.2629
Blautia_A.obeum	61.5	-1.186	0.669	-2.498	0.125	0.0761	0.3388
Agathobacter.sp900546625	61.5	1.003	0.582	-0.139	2.145	0.0851	0.3442
Intestinibacter.bartlettii	30.8	-0.851	0.493	-1.818	0.115	0.0841	0.3442
Anaerostipes.hadrus	57.7	1.136	0.671	-0.179	2.452	0.0904	0.3498
Streptococcus.sp001556435	38.5	-0.894	0.536	-1.945	0.156	0.0952	0.3532
Mediterraneibacter.torques	65.4	1.621	1.005	-0.350	3.591	0.1069	0.3807
Enterocloster.clostridioformis	42.3	1.318	0.861	-0.370	3.006	0.1259	0.4310
Bifidobacterium.pseudocatenulatum	57.7	-1.122	0.761	-2.614	0.369	0.1403	0.4625
Agathobacter.rectalis	46.2	0.451	0.317	-0.170	1.071	0.1547	0.4917
Ruminococcus_D.bicirculans	69.2	1.149	0.835	-0.487	2.785	0.1686	0.5174
Anaerobutyricum.hallii	61.5	0.886	0.672	-0.431	2.203	0.1871	0.5551
Lachnospira.eligens_B	69.2	0.967	0.770	-0.543	2.477	0.2096	0.5725
Dialister.invisus	69.2	-1.343	1.066	-3.433	0.747	0.2078	0.5725
Ruthenibacterium.lactatiformans	42.3	-0.652	0.522	-1.675	0.372	0.2123	0.5725
Bacteroides.ovatus	50.0	1.298	1.101	-0.860	3.456	0.2383	0.5891
Dorea.faecis	69.2	-0.577	0.488	-1.534	0.380	0.2370	0.5891
Anaeromassilibacillus.sp002159845	65.4	-0.762	0.637	-2.010	0.485	0.2310	0.5891
Streptococcus.vestibularis	61.5	-0.851	0.741	-2.303	0.601	0.2506	0.6006
Bilophila.wadsworthia	53.8	0.725	0.669	-0.587	2.036	0.2787	0.6006
Acetatifactor.sp900066565	61.5	0.740	0.666	-0.566	2.046	0.2670	0.6006
Faecalimonas.nexilis	61.5	-1.083	1.004	-3.051	0.885	0.2808	0.6006
Enterococcus_B.faecium	65.4	0.607	0.555	-0.480	1.694	0.2734	0.6006
Faecalibacterium.prausnitzii_G	69.2	1.127	1.069	-0.967	3.222	0.2914	0.6006
Parabacteroides.distasonis	38.5	0.800	0.775	-0.719	2.318	0.3021	0.6006
Ruminococcus_B.gnavus	19.2	-0.537	0.521	-1.558	0.484	0.3024	0.6006
f_Oscillospiraceae.g_Oscillibacter.s_unknown	61.5	0.717	0.713	-0.679	2.114	0.3141	0.6006
Escherichia.flexneri	15.4	-0.586	0.579	-1.722	0.550	0.3118	0.6006
f_Lachnospiraceae.g_Faecalimonas.s_unknown	65.4	-0.848	0.848	-2.511	0.814	0.3172	0.6006
Fusicatenibacter.saccharivorans	53.8	-0.484	0.559	-1.579	0.611	0.3861	0.6892
Enterocloster.sp001517625	65.4	1.169	1.373	-1.522	3.861	0.3946	0.6892
Bacteroides.fragilis	57.7	0.914	1.095	-1.232	3.060	0.4038	0.6892
Erysipelatoclostridium.spiroforme	69.2	-0.645	0.783	-2.180	0.890	0.4102	0.6892
Enterocloster.sp900541315	69.2	-0.506	0.644	-1.769	0.757	0.4325	0.6892
Streptococcus.thermophilus	38.5	-0.579	0.735	-2.019	0.862	0.4310	0.6892
Lawsonibacter.sp900066825	61.5	0.619	0.797	-0.943	2.182	0.4373	0.6892
f_Bacteroidaceae.g_Phocaeicola.s_unknown	50.0	0.369	0.499	-0.609	1.346	0.4600	0.6892
Blautia_A.sp900066165	42.3	-0.429	0.580	-1.567	0.709	0.4600	0.6892
Enterocloster.bolteae	19.2	-0.425	0.566	-1.535	0.685	0.4530	0.6892
Enterocloster.citroniae	46.2	0.403	0.535	-0.646	1.451	0.4516	0.6892
Agathobaculum.sp900291975	53.8	-0.243	0.322	-0.873	0.388	0.4507	0.6892
Bacteroides.caccae	61.5	0.413	0.582	-0.728	1.553	0.4785	0.6892
Faecalibacterium.prausnitzii_D	57.7	0.451	0.645	-0.814	1.715	0.4848	0.6892

Blautia_A.sp000433815	53.8	0.587	0.878	-1.133	2.307	0.5033	0.6892
CAG.81.sp900066785	69.2	0.585	0.871	-1.122	2.292	0.5019	0.6892
Faecalimonas.umbilicata	61.5	0.557	0.819	-1.049	2.162	0.4969	0.6892
Hungatella.sp005845265	65.4	0.548	0.817	-1.054	2.150	0.5025	0.6892
Blautia_A.massiliensis	57.7	0.478	0.729	-0.950	1.906	0.5121	0.6905
Ruminococcus_E.bromii_B	69.2	0.404	0.663	-0.896	1.703	0.5424	0.7205
KLE1615.sp900066985	69.2	-0.715	1.214	-3.094	1.664	0.5556	0.7260
Longicatena.innocuum	26.9	-0.376	0.661	-1.673	0.920	0.5694	0.7260
CAG.41.sp900066215	50.0	0.380	0.679	-0.951	1.710	0.5760	0.7260
Alistipes.putredinis	65.4	-0.305	0.549	-1.381	0.772	0.5792	0.7260
Clostridium_Q.symbiosum	30.8	-0.347	0.711	-1.741	1.046	0.6251	0.7727
Lawsonibacter.asaccharolyticus	53.8	-0.164	0.379	-0.907	0.579	0.6652	0.8110
Roseburia.hominis	65.4	0.514	1.343	-2.118	3.146	0.7019	0.8410
Faecalibacterium.prausnitzii_C	57.7	-0.227	0.608	-1.419	0.965	0.7087	0.8410
Bacteroides.uniformis	38.5	-0.284	0.835	-1.921	1.352	0.7333	0.8588
Enterocloster.aldenensis	26.9	0.129	0.470	-0.792	1.049	0.7839	0.9002
Bacteroides.thetaiotaomicron	53.8	-0.106	0.406	-0.902	0.690	0.7947	0.9002
Acutalibacter.sp900543555	69.2	0.251	0.996	-1.701	2.202	0.8012	0.9002
Flavonifractor.plautii	7.7	0.076	0.315	-0.542	0.695	0.8091	0.9002
Alistipes.onderdonkii	53.8	0.217	0.958	-1.660	2.094	0.8208	0.9018
Parabacteroides.merdae	57.7	-0.133	0.632	-1.372	1.106	0.8331	0.9042
Agathobaculum.butyriciproducens	69.2	0.134	0.707	-1.252	1.520	0.8498	0.9112
Anaerostipes.caccae	53.8	-0.084	0.672	-1.400	1.233	0.9008	0.9544
Eggerthella.lenta	38.5	0.037	0.394	-0.734	0.809	0.9248	0.9581
Dorea.sp000509125	65.4	-0.060	0.706	-1.443	1.323	0.9321	0.9581
Veillonella.parvula_A	65.4	0.081	1.019	-1.916	2.078	0.9365	0.9581
Anaerotruncus.colihominis	61.5	0.031	0.671	-1.284	1.346	0.9630	0.9734
Erysipelatoclostridium.ramosum	42.3	0.020	0.611	-1.178	1.219	0.9734	0.9734

Non-remission week 22

Species	ZP (%)	Coefficient	Std.error	Lower 95% CI	Higher 95% CI	p-Value	q-Value
Sutterella.wadsworthensis	62.5	2.868	0.418	2.048	3.687	6.83E-12	1.10E-09
f_Bacteroidaceae.g_Bacteroides.s_unknown	41.7	1.368	0.323	0.736	2.000	2.22E-05	0.0018
Bacteroides.thetaiotaomicron	33.3	1.619	0.465	0.708	2.531	4.97E-04	0.0212
Ruthenibacterium.lactatiformans	33.3	-1.983	0.572	-3.104	-0.862	5.26E-04	0.0212
Dorea.formicigenerans	25.0	-1.237	0.378	-1.979	-0.496	0.0011	0.0247
GCA.900066135.sp900543575	62.5	2.308	0.705	0.927	3.690	0.0011	0.0247
Streptococcus.thermophilus	54.2	-3.036	0.921	-4.841	-1.231	0.0010	0.0247
Mediterraneibacter.torques	25.0	-2.019	0.628	-3.249	-0.788	0.0013	0.0257
UMGS1071.sp900542375	45.8	-1.305	0.409	-2.107	-0.502	0.0014	0.0257
Eggerthella.lenta	58.3	-1.745	0.567	-2.857	-0.634	0.0021	0.0336
Blautia_A.sp900066355	29.2	-1.524	0.509	-2.521	-0.526	0.0028	0.0344
Dorea.longicatena	41.7	-1.527	0.510	-2.527	-0.526	0.0028	0.0344
UBA1191.sp900549125	50.0	-1.607	0.530	-2.646	-0.568	0.0024	0.0344
f_Lachnospiraceae.g_UBA7160.s_unknown	54.2	-1.225	0.445	-2.097	-0.354	0.0059	0.0667
CAG.103.sp900317855	58.3	-2.428	0.887	-4.167	-0.689	0.0062	0.0667
Anaerobutyricum.hallii	33.3	-1.462	0.568	-2.575	-0.349	0.0100	0.0962
f_Lachnospiraceae.g_Blautia_A.s_unknown	54.2	1.435	0.561	0.335	2.535	0.0106	0.0962
Mediterraneibacter.lactaris	54.2	-1.340	0.526	-2.371	-0.310	0.0108	0.0962
Clostridium_A.leptum	66.7	-3.761	1.502	-6.705	-0.816	0.0123	0.1042
Dorea.sp900066555	54.2	-1.319	0.532	-2.361	-0.277	0.0131	0.1054
CAG.170.sp900545925	58.3	-2.180	0.893	-3.931	-0.430	0.0146	0.1122
Blautia_A.sp900066145	54.2	-0.776	0.323	-1.410	-0.142	0.0164	0.1198
Lachnospira.rogosae	54.2	1.451	0.647	0.182	2.719	0.0250	0.1752
Ruminococcus_H.sp003531055	54.2	-1.569	0.710	-2.961	-0.176	0.0272	0.1828
Eubacterium_I.ramulus_A	62.5	-1.268	0.580	-2.404	-0.131	0.0288	0.1855
Clostridium_Q.symbiosum	62.5	-1.269	0.591	-2.428	-0.110	0.0319	0.1872
Dorea.sp000433215	58.3	-1.250	0.590	-2.407	-0.094	0.0340	0.1872
Hungatella.effluvii	66.7	-1.466	0.692	-2.822	-0.110	0.0341	0.1872
Longicatena.innocuum	50.0	-1.570	0.738	-3.016	-0.123	0.0334	0.1872
Eubacterium_I.ramulus	58.3	-0.836	0.396	-1.613	-0.059	0.0349	0.1872
CAG.217.sp000436335	66.7	-1.914	0.922	-3.722	-0.107	0.0379	0.1968
Blautia_A.wexlerae	62.5	-2.024	0.998	-3.980	-0.068	0.0425	0.2139
Bacteroides.uniformis	8.3	1.021	0.506	0.028	2.014	0.0438	0.2139
Blautia_A.sp900066505	62.5	-1.532	0.775	-3.052	-0.013	0.0480	0.2155

Enterocloster.sp900541315	25.0	-0.820	0.418	-1.639	-0.002	0.0495	0.2155
Faecalibacterium.prausnitzii_D	33.3	1.221	0.620	0.005	2.436	0.0490	0.2155
Streptococcus.sp001556435	62.5	-1.527	0.769	-3.034	-0.020	0.0470	0.2155
Bacteroides.cellulosilyticus	41.7	1.974	1.016	-0.018	3.965	0.0521	0.2207
Anaerotignum.sp000436415	50.0	-1.451	0.768	-2.956	0.055	0.0589	0.2433
CAG.279.sp000437795	66.7	0.745	0.401	-0.041	1.531	0.0634	0.2550
Faecalibacterium.prausnitzii_A	66.7	-1.577	0.874	-3.290	0.136	0.0712	0.2790
Prevotella.copri	66.7	2.111	1.177	-0.195	4.417	0.0728	0.2790
Bacteroides.salyersiae	62.5	-0.902	0.516	-1.914	0.109	0.0803	0.3006
Butyricimonas.sp002161485	66.7	1.576	0.918	-0.224	3.377	0.0861	0.3149
Bariatricus.comes	25.0	-0.702	0.419	-1.522	0.119	0.0937	0.3220
Blautia_A.obeum	25.0	-0.853	0.509	-1.851	0.145	0.0940	0.3220
CAG.110.sp003525905	50.0	-0.969	0.574	-2.095	0.156	0.0914	0.3220
Lawsonibacter.sp900066825	66.7	-0.804	0.493	-1.770	0.162	0.1029	0.3407
SFFH01.sp900542395	66.7	-1.371	0.845	-3.026	0.285	0.1046	0.3407
SFFH01.sp900542445	62.5	-1.137	0.703	-2.514	0.241	0.1058	0.3407
Faecalibacterium.prausnitzii_J	66.7	-1.213	0.794	-2.769	0.344	0.1267	0.3857
Romboutsia.timonensis	62.5	0.768	0.495	-0.203	1.739	0.1209	0.3816
Parabacteroides.distasonis	41.7	0.963	0.632	-0.276	2.202	0.1277	0.3857
Roseburia.hominis	37.5	-0.808	0.536	-1.859	0.243	0.1318	0.3857
Agathobacter.sp900546625	66.7	-0.809	0.542	-1.872	0.253	0.1355	0.3857
Mediterraneibacter.faecis	66.7	-1.267	0.847	-2.927	0.393	0.1347	0.3857
Bacteroides.ovatus	45.8	0.649	0.436	-0.205	1.503	0.1365	0.3857
Alistipes.onderdonkii	50.0	-1.189	0.807	-2.772	0.393	0.1407	0.3906
Agathobaculum.butyriciproducens	37.5	-0.664	0.471	-1.586	0.259	0.1585	0.4326
Faecalibacterium.sp900539945	62.5	1.410	1.053	-0.654	3.474	0.1806	0.4847
Fusicatenibacter.saccharivorans	25.0	-0.478	0.363	-1.189	0.234	0.1881	0.4909
Ruminococcus_E.bromii_B	58.3	1.113	0.848	-0.548	2.775	0.1890	0.4909
CAG.170.sp000432135	45.8	-0.804	0.637	-2.052	0.444	0.2067	0.5282
Bifidobacterium.longum	25.0	0.563	0.483	-0.384	1.511	0.2439	0.6136
Eubacterium_G.ventriosum	62.5	-1.335	1.172	-3.631	0.962	0.2547	0.6308
UBA9502.sp003480315	66.7	-0.616	0.554	-1.701	0.469	0.2658	0.6484
Clostridium_Q.sp003024715	25.0	-0.562	0.515	-1.570	0.447	0.2751	0.6548
Bacteroides.stercoris	33.3	-0.381	0.352	-1.072	0.309	0.2791	0.6548
Erysipelatoclostridium.ramosum	58.3	-1.114	1.032	-3.137	0.909	0.2806	0.6548
Gemmiger.qucibialis	33.3	-0.620	0.590	-1.776	0.536	0.2931	0.6732
Agathobacter.faecis	33.3	0.678	0.659	-0.613	1.969	0.3034	0.6732
UMGS1375.sp900066615	58.3	-0.888	0.860	-2.574	0.798	0.3018	0.6732
Erysipelatoclostridium.sp000752095	66.7	-0.704	0.690	-2.057	0.648	0.3074	0.6732
Ruminococcus_A.sp003011855	58.3	-0.690	0.679	-2.021	0.641	0.3094	0.6732
Blautia_A.sp900120195	58.3	-0.563	0.563	-1.666	0.539	0.3167	0.6776
f_Ruminococcaceae.g_Faecalibacterium.s_unknown	45.8	-0.931	0.936	-2.765	0.903	0.3199	0.6776
CAG.81.sp900066785	66.7	0.672	0.686	-0.673	2.016	0.3275	0.6848
Akkermansia.muciniphila	45.8	-0.757	0.791	-2.308	0.794	0.3387	0.6992
Butyricimonas.faecihominis	45.8	0.475	0.517	-0.538	1.488	0.3579	0.7107
Enterocloster.bolteae	41.7	-0.675	0.726	-2.099	0.749	0.3526	0.7097
ER4.sp000765235	62.5	-0.752	0.806	-2.332	0.827	0.3503	0.7097
Flavonifractor.plautii	25.0	-0.476	0.522	-1.500	0.547	0.3620	0.7107
UBA11774.sp003507655	45.8	-0.605	0.686	-1.950	0.739	0.3776	0.7325
Parasutterella.excrementihominis	66.7	-0.409	0.469	-1.329	0.510	0.3833	0.7346
CAG.81.sp900066535	37.5	-0.405	0.479	-1.344	0.534	0.3982	0.7387
f_Lachnospiraceae.g_unknown.s_unknown	66.7	-0.580	0.688	-1.928	0.768	0.3992	0.7387
Anaerotruncus.colihominis	62.5	-0.560	0.662	-1.859	0.738	0.3976	0.7387
Bilophila.wadsworthia	58.3	-0.525	0.650	-1.799	0.749	0.4193	0.7586
Eubacterium_F.sp003491505	50.0	-0.765	0.947	-2.621	1.090	0.4188	0.7586
UMGS966.sp900547185	41.7	-0.373	0.475	-1.305	0.558	0.4322	0.7731
Lachnospira.eligens_B	41.7	-0.632	0.835	-2.269	1.005	0.4493	0.7878
Alistipes.obesi	50.0	0.545	0.739	-0.905	1.994	0.4614	0.7878
Faecalibacterium.sp900539885	62.5	-0.282	0.380	-1.027	0.463	0.4578	0.7878
Ruminococcus_D.bicirculans	54.2	0.603	0.823	-1.011	2.217	0.4639	0.7878
Alistipes.finegoldii	41.7	-0.522	0.717	-1.928	0.884	0.4670	0.7878
TF01.11.sp003529475	58.3	-0.573	0.792	-2.125	0.980	0.4698	0.7878
Ruthenibacterium.sp900546885	62.5	-0.605	0.850	-2.272	1.061	0.4766	0.7910
ER4.sp900317525	66.7	-0.753	1.079	-2.869	1.362	0.4852	0.7971
Alistipes.shahii	45.8	-0.428	0.685	-1.769	0.914	0.5323	0.8302

Agathobacter.rectalis	33.3	-0.370	0.598	-1.543	0.803	0.5361	0.8302
Blautia_A.sp000285855	45.8	-0.289	0.458	-1.187	0.609	0.5288	0.8302
CAG.45.sp900066395	50.0	0.721	1.157	-1.547	2.989	0.5330	0.8302
Gemmiger.formicilis	50.0	-0.314	0.498	-1.290	0.662	0.5286	0.8302
Enterocloster.sp000431375	54.2	-0.393	0.641	-1.649	0.863	0.5398	0.8302
Ruminococcus_D.sp900539095	66.7	-0.397	0.650	-1.670	0.877	0.5415	0.8302
Faecalibacterium.prausnitzii_C	50.0	0.463	0.781	-1.068	1.995	0.5531	0.8400
Bacteroides.xylanisolvens	58.3	-0.250	0.451	-1.134	0.634	0.5800	0.8463
Odoribacter.splanchnicus	37.5	-0.254	0.500	-1.234	0.726	0.6112	0.8463
Alistipes_A.indistinctus	54.2	-0.364	0.711	-1.757	1.030	0.6089	0.8463
Alistipes.putredinis	33.3	0.311	0.597	-0.859	1.481	0.6025	0.8463
Anaerostipes.hadrus	25.0	-0.245	0.466	-1.159	0.668	0.5988	0.8463
CAG.81.sp900066055	58.3	-0.289	0.536	-1.340	0.762	0.5902	0.8463
Eisenbergiella.sp900066775	37.5	0.294	0.557	-0.798	1.385	0.5980	0.8463
Enterocloster.clostridioformis	45.8	0.274	0.540	-0.784	1.331	0.6122	0.8463
Roseburia.intestinalis	41.7	0.433	0.815	-1.165	2.031	0.5951	0.8463
Faecalibacterium.prausnitzii_G	41.7	0.275	0.517	-0.739	1.288	0.5950	0.8463
Blautia_A.sp900066205	58.3	0.307	0.610	-0.888	1.501	0.6150	0.8463
Gemmiger.sp900539695	62.5	0.272	0.555	-0.815	1.360	0.6232	0.8503
CAG.115.sp003531585	66.7	-0.334	0.701	-1.708	1.041	0.6342	0.8558
Streptococcus.salivarius	58.3	-0.448	0.952	-2.314	1.418	0.6379	0.8558
Bacteroides.fragilis	66.7	0.381	0.901	-1.385	2.146	0.6724	0.8633
Phocaeicola.vulgatus	8.3	0.137	0.345	-0.539	0.814	0.6907	0.8633
Alistipes_A.ihumii	50.0	-0.267	0.651	-1.543	1.009	0.6819	0.8633
Acetatifactor.sp900066365	54.2	0.297	0.748	-1.168	1.763	0.6907	0.8633
Anaerobutyricum.hallii_A	25.0	-0.176	0.428	-1.014	0.663	0.6816	0.8633
Enterocloster.aldenensis	54.2	0.286	0.705	-1.097	1.668	0.6855	0.8633
Roseburia.inulinivorans	41.7	0.276	0.695	-1.087	1.639	0.6917	0.8633
Ruminococcus_B.gnavus	29.2	-0.339	0.790	-1.888	1.210	0.6679	0.8633
Escherichia.flexneri	16.7	-0.239	0.573	-1.363	0.885	0.6770	0.8633
CAG.83.sp000431575	62.5	-0.259	0.684	-1.599	1.081	0.7047	0.8728
Barnesiella.intestinihominis	50.0	0.179	0.580	-0.958	1.317	0.7573	0.8923
Hungatella_A.sp003478355	66.7	-0.237	0.735	-1.678	1.204	0.7472	0.8923
UBA7182.sp003480725	54.2	-0.202	0.626	-1.430	1.026	0.7470	0.8923
UBA7182.sp003481535	62.5	-0.173	0.544	-1.240	0.894	0.7507	0.8923
UBA9502.sp003478505	62.5	-0.226	0.651	-1.501	1.050	0.7288	0.8923
f_Oscillospiraceae.g_Oscillibacter.s_unknown	54.2	-0.196	0.622	-1.416	1.024	0.7528	0.8923
f_Lachnospiraceae.g_CAG-81.s_unknown	62.5	-0.167	0.571	-1.285	0.951	0.7697	0.8923
f_Bacteroidaceae.g_Phocaeicola.s_unknown	33.3	0.109	0.380	-0.636	0.854	0.7741	0.8923
Parabacteroides.merdae	45.8	0.153	0.542	-0.908	1.215	0.7774	0.8923
Bifidobacterium.bifidum	62.5	0.170	0.631	-1.067	1.406	0.7879	0.8923
Phocaeicola.massiliensis	62.5	-0.207	0.764	-1.705	1.290	0.7861	0.8923
f_Lachnospiraceae.g_Faecalimonas.s_unknown	66.7	-0.334	1.269	-2.821	2.154	0.7925	0.8923
Oscillibacter.welbionis	41.7	0.161	0.592	-0.999	1.322	0.7851	0.8923
Faecalibacterium.prausnitzii	66.7	-0.172	0.713	-1.569	1.225	0.8094	0.9050
Acetatifactor.sp900066565	29.2	0.139	0.651	-1.136	1.415	0.8305	0.9195
Oscillibacter.sp001916835	50.0	0.146	0.697	-1.220	1.512	0.8339	0.9195
Blautia_A.sp900066165	29.2	0.066	0.371	-0.662	0.794	0.8592	0.9351
Lachnospira.sp000437735	66.7	0.137	0.795	-1.421	1.695	0.8632	0.9351
CAG.41.sp900066215	41.7	-0.075	0.444	-0.945	0.795	0.8654	0.9351
Acidaminococcus.intestini	50.0	-0.088	0.639	-1.340	1.164	0.8907	0.9560
Bifidobacterium.adolescentis	66.7	0.104	0.956	-1.770	1.977	0.9136	0.9627
Blautia_A.sp900066335	58.3	0.059	0.697	-1.307	1.424	0.9329	0.9627
Evtepia.sp900546255	58.3	-0.065	0.669	-1.377	1.247	0.9222	0.9627
Lawsonibacter.asaccharolyticus	37.5	-0.041	0.447	-0.918	0.835	0.9264	0.9627
Intestinibacter.bartlettii	41.7	0.043	0.477	-0.891	0.977	0.9279	0.9627
CAG.81.sp000435795	37.5	-0.046	0.604	-1.229	1.137	0.9397	0.9627
CAG.103.sp000432375	54.2	0.047	0.658	-1.243	1.337	0.9431	0.9627
KLE1615.sp900066985	50.0	0.046	0.669	-1.265	1.358	0.9448	0.9627
Blautia_A.massiliensis	29.2	-0.009	0.425	-0.843	0.824	0.9825	0.9859
f_Lachnospiraceae.g_KLE1615.s_unknown	58.3	0.011	0.630	-1.224	1.247	0.9859	0.9859
f_Lachnospiraceae.g_Mediterraneibacter.s_unknown	37.5	0.016	0.499	-0.963	0.994	0.9752	0.9859
