# Supplementary material for: Phage-Display Immunoprecipitation Sequencing Reveals Distinct Antibody Signatures Against Bacterial Flagellins Associated With Treatment Response in Crohn's Disease
Source: Clin Transl Gastroenterol. 2026 Apr 2;17(6):e01030. doi: 10.14309/ctg.0000000000001030 (PMC13641668; doi:10.14309/ctg.0000000000001030)
Supplement: Supplementary file 4 [file ct9-17-e01030-s004.pdf]

Supplementary Table 4

Remission week 52

Species	ZP (%)	Coefficient	Std.error	Lower 95% CI	Higher 95% CI	p-Value	q-Value
Veillonella.parvula_A	56.3	-3.683	0.640	-4.938	-2.428	8.85E-09	1.29E-06
Erysipelatoclostridium.spiroforme	62.5	-6.412	1.297	-8.953	-3.871	7.60E-07	5.55E-05
f_Lachnospiraceae.g_Faecalimonas.s_unknown	62.5	-3.384	0.700	-4.756	-2.012	1.33E-06	6.49E-05
Agathobacter.faecis	50.0	2.289	0.518	1.274	3.304	9.81E-06	3.05E-04
Ruminococcus_B.gnavus	25.0	-1.101	0.250	-1.590	-0.611	1.04E-05	3.05E-04
GCA.900066135.sp900543575	43.8	-1.983	0.596	-3.151	-0.816	8.68E-04	0.0181
Klebsiella.pneumoniae	68.8	-3.870	1.152	-6.128	-1.613	7.78E-04	0.0181
Anaerostipes.hadrus	31.3	2.054	0.642	0.796	3.312	0.0014	0.0251
Roseburia.inulinivorans	50.0	2.886	0.949	1.026	4.745	0.0023	0.0365
Lachnospira.eligens_B	50.0	1.203	0.399	0.421	1.985	0.0026	0.0365
Gemmiger.qucibialis	43.8	2.491	0.832	0.861	4.121	0.0027	0.0365
Coprobacter.fastidiosus	62.5	3.249	1.185	0.928	5.571	0.0061	0.0592
Anaerostipes.caccae	50.0	-1.777	0.647	-3.044	-0.509	0.0060	0.0592
Hungatella.effluvii	68.8	-3.288	1.186	-5.613	-0.963	0.0056	0.0592
f_Lachnospiraceae.g_unknown.s_unknown	56.3	-2.064	0.751	-3.536	-0.592	0.0060	0.0592
Streptococcus.sp001556435	43.8	-1.901	0.705	-3.282	-0.519	0.0070	0.0639
f_Lachnospiraceae.g_Blautia_A.s_unknown	62.5	4.377	1.681	1.083	7.671	0.0092	0.0790
Faecalibacterium.prausnitzii_C	31.3	1.908	0.738	0.461	3.355	0.0097	0.0791
CAG.41.sp900066215	37.5	1.364	0.537	0.311	2.417	0.0111	0.0813
Streptococcus.salivarius	25.0	-1.446	0.569	-2.561	-0.330	0.0111	0.0813
Erysipelatoclostridium.ramosum	50.0	-2.414	0.965	-4.305	-0.523	0.0124	0.0859
Escherichia.flexneri	12.5	-1.677	0.690	-3.029	-0.325	0.0151	0.1000
Enterocloster.aldenensis	31.3	-0.756	0.322	-1.387	-0.124	0.0190	0.1158
f_Lachnospiraceae.g_Mediterraneibacter.s_unknown	43.8	-0.826	0.352	-1.516	-0.136	0.0189	0.1158
Ruminococcus_A.sp003011855	50.0	-1.574	0.678	-2.903	-0.245	0.0202	0.1182
Bacteroides.thetaiotaomicron	50.0	3.466	1.513	0.500	6.431	0.0220	0.1235
Ruthenibacterium.sp900546885	68.8	-1.997	0.939	-3.837	-0.157	0.0334	0.1805
Roseburia.hominis	43.8	-1.291	0.616	-2.499	-0.082	0.0363	0.1892
Parabacteroides.distasonis	50.0	1.904	0.937	0.068	3.740	0.0421	0.2119
Dorea.sp900066555	62.5	1.982	0.997	0.028	3.936	0.0468	0.2276
Alistipes.onderdonkii	56.3	1.386	0.719	-0.024	2.796	0.0540	0.2322
Oscillibacter.welbionis	37.5	1.224	0.634	-0.017	2.466	0.0533	0.2322
Faecalibacterium.sp900539945	50.0	2.126	1.104	-0.038	4.290	0.0542	0.2322
Erysipelatoclostridium.sp000752095	68.8	2.541	1.309	-0.025	5.107	0.0523	0.2322
Parabacteroides.merdae	56.3	1.519	0.794	-0.037	3.074	0.0557	0.2322
Phocaeicola.vulgatus	37.5	1.390	0.747	-0.075	2.855	0.0629	0.2353
Blautia_A.hydrogenotrophica	62.5	-1.429	0.764	-2.927	0.068	0.0614	0.2353
Fusicatenibacter.saccharivorans	25.0	-0.882	0.472	-1.806	0.043	0.0616	0.2353
Lawsonibacter.asaccharolyticus	43.8	1.381	0.736	-0.062	2.824	0.0607	0.2353
CAG.81.sp900066535	43.8	1.087	0.591	-0.071	2.245	0.0658	0.2384
Ruminococcus_D.bicirculans	62.5	2.722	1.495	-0.208	5.651	0.0686	0.2384
UMGS966.sp900547185	43.8	1.095	0.601	-0.082	2.272	0.0683	0.2384
Lachnospira.sp000437735	56.3	2.218	1.230	-0.192	4.628	0.0713	0.2421
Lawsonibacter.sp900066645	68.8	1.932	1.096	-0.217	4.080	0.0780	0.2568
Gemmiger.sp900540775	68.8	2.807	1.601	-0.330	5.945	0.0795	0.2568
Ruminococcus_D.sp900539095	68.8	2.133	1.222	-0.262	4.529	0.0809	0.2568
Bacteroides.ovatus	50.0	1.263	0.730	-0.168	2.695	0.0837	0.2599
Alistipes.putredinis	50.0	1.662	0.967	-0.233	3.556	0.0856	0.2604
Eisenbergiella.sp900066775	43.8	1.721	1.023	-0.284	3.727	0.0925	0.2756
Enterocloster.bolteae	25.0	-1.354	0.839	-2.998	0.290	0.1065	0.3110
Acetatifactor.sp003447295	68.8	1.282	0.809	-0.305	2.868	0.1133	0.3193
Lawsonibacter.sp900066825	50.0	1.163	0.736	-0.278	2.605	0.1137	0.3193
Gordonibacter.urolithinfaciens	62.5	1.307	0.871	-0.400	3.014	0.1335	0.3622
Acetatifactor.sp900066565	50.0	1.185	0.791	-0.365	2.736	0.1340	0.3622
Eubacterium_F.sp003491505	56.3	1.641	1.128	-0.570	3.852	0.1457	0.3831
Bilophila.wadsworthia	43.8	0.681	0.469	-0.239	1.601	0.1470	0.3831
Enterocloster.clostridioformis	50.0	0.682	0.475	-0.249	1.613	0.1512	0.3872
Faecalibacterium.prausnitzii_J	68.8	-0.657	0.470	-1.578	0.264	0.1623	0.4054
Alistipes.finegoldii	62.5	1.382	0.993	-0.564	3.328	0.1638	0.4054
Holdemania.massiliensis	62.5	-1.117	0.837	-2.756	0.523	0.1819	0.4427
Longicatena.innocuum	25.0	-1.019	0.770	-2.529	0.490	0.1856	0.4443
Bifidobacterium.adolescentis	68.8	3.323	2.551	-1.677	8.323	0.1927	0.4484
Faecalibacterium.prausnitzii	68.8	1.178	0.906	-0.597	2.952	0.1935	0.4484
Negativibacillus.massiliensis	68.8	-0.911	0.719	-2.320	0.498	0.2051	0.4679
f_Bacteroidaceae.g_Phocaeicola.s_unknown	50.0	0.667	0.538	-0.387	1.722	0.2149	0.4827
f_Bacteroidaceae.g_Bacteroides.s_unknown	68.8	1.081	0.943	-0.768	2.929	0.2518	0.5487
Sutterella.wadsworthensis	68.8	1.166	1.013	-0.819	3.151	0.2498	0.5487

Anaerobutyricum.hallii	37.5	0.769	0.685	-0.573	2.111	0.2616	0.5560
Streptococcus.parasanguinis_B	68.8	-1.541	1.376	-4.237	1.156	0.2628	0.5560
Phocaeicola.massiliensis	68.8	-1.035	0.962	-2.920	0.850	0.2818	0.5878
Agathobaculum.butyriciproducens	68.8	0.962	0.926	-0.853	2.776	0.2989	0.6003
Streptococcus.vestibularis	56.3	-0.836	0.805	-2.415	0.742	0.2992	0.6003
Eubacterium_R.sp000436835	62.5	0.910	0.883	-0.820	2.640	0.3023	0.6003
Agathobacter.rectalis	31.3	0.799	0.785	-0.738	2.337	0.3083	0.6003
Blautia_A.massiliensis	37.5	0.693	0.680	-0.641	2.027	0.3084	0.6003
Blautia_A.sp000436615	68.8	0.529	0.532	-0.513	1.570	0.3199	0.6146
Ruminiclostridium_E.siraeum	68.8	1.117	1.140	-1.117	3.351	0.3272	0.6204
f_Acutalibacteraceae.g_UMGS1623.s_unknown	68.8	-0.823	0.856	-2.500	0.855	0.3367	0.6302
Bariatricus.comes	62.5	-0.466	0.490	-1.425	0.494	0.3413	0.6308
Enterocloster.sp000431375	68.8	0.613	0.658	-0.676	1.902	0.3516	0.6403
Eubacterium_G.ventriosum	68.8	0.625	0.676	-0.700	1.951	0.3552	0.6403
f_Oscillospiraceae.g_Oscillibacter.s_unknown	43.8	0.479	0.530	-0.559	1.518	0.3655	0.6468
Mediterraneibacter.torques	31.3	0.609	0.678	-0.720	1.938	0.3690	0.6468
Blautia_A.sp000285855	68.8	-0.638	0.717	-2.042	0.766	0.3731	0.6468
Dorea.faecis	62.5	-0.882	0.998	-2.838	1.073	0.3766	0.6468
Enterocloster.sp900541315	50.0	-0.604	0.698	-1.972	0.764	0.3870	0.6495
Lactococcus.lactis	62.5	0.726	0.841	-0.922	2.373	0.3880	0.6495
Barnesiella.intestinihominis	56.3	0.328	0.382	-0.422	1.077	0.3915	0.6495
Ruminococcus_H.sp003531055	68.8	-1.366	1.627	-4.554	1.823	0.4013	0.6579
CAG.110.sp003525905	68.8	0.724	0.871	-0.982	2.431	0.4056	0.6579
Faecalibacterium.prausnitzii_G	50.0	0.551	0.671	-0.764	1.866	0.4117	0.6597
Clostridium_Q.symbiosum	56.3	-1.526	1.885	-5.220	2.168	0.4181	0.6597
Intestinibacter.bartlettii	18.8	-0.334	0.414	-1.146	0.478	0.4202	0.6597
Blautia_A.sp900066165	37.5	0.449	0.574	-0.677	1.574	0.4344	0.6747
Anaeromassilibacillus.sp002159845	62.5	0.766	1.000	-1.193	2.725	0.4437	0.6819
Bacteroides.cellulosilyticus	62.5	0.620	0.860	-1.066	2.305	0.4713	0.6897
Bacteroides.uniformis	25.0	-0.491	0.692	-1.846	0.865	0.4781	0.6897
Butyricimonas.faecihominis	68.8	0.649	0.900	-1.116	2.413	0.4710	0.6897
Blautia_A.wexlerae	56.3	-0.500	0.686	-1.845	0.845	0.4662	0.6897
Evtepia.gabavorous	68.8	0.714	0.984	-1.213	2.642	0.4676	0.6897
f_Dialisteraceae.g_Dialister.s_unknown	62.5	0.692	0.975	-1.220	2.603	0.4782	0.6897
Streptococcus.thermophilus	25.0	-0.631	0.898	-2.391	1.128	0.4818	0.6897
Dorea.formicigenerans	37.5	0.401	0.593	-0.763	1.564	0.4996	0.7013
f_Ruminococcaceae.g_Faecalibacterium.s_unknown	62.5	0.784	1.152	-1.474	3.041	0.4962	0.7013
Eubacterium_I.ramulus	68.8	0.659	0.995	-1.291	2.608	0.5078	0.7061
Odoribacter.splanchnicus	62.5	0.452	0.705	-0.931	1.834	0.5218	0.7187
Bacteroides.stercoris	43.8	-0.518	0.880	-2.243	1.206	0.5559	0.7585
Bifidobacterium.longum	31.3	0.354	0.619	-0.859	1.566	0.5677	0.7649
Intestinimonas.butyriciproducens	62.5	0.404	0.713	-0.993	1.801	0.5711	0.7649
Roseburia.intestinalis	62.5	0.429	0.782	-1.104	1.963	0.5831	0.7739
Acutalibacter.sp000435395	68.8	0.698	1.305	-1.861	3.256	0.5930	0.7799
UBA1191.sp900549125	43.8	-0.242	0.462	-1.147	0.664	0.6005	0.7828
Dorea.longicatena	37.5	0.271	0.530	-0.769	1.310	0.6100	0.7839
Faecalibacterium.sp900539885	68.8	-0.363	0.719	-1.772	1.047	0.6139	0.7839
Dialister.invisus	56.3	0.502	1.011	-1.480	2.483	0.6196	0.7839
Dorea.sp000433535	68.8	-0.698	1.418	-3.477	2.082	0.6228	0.7839
Agathobacter.sp900546625	68.8	-0.426	0.890	-2.170	1.317	0.6318	0.7884
Faecalibacterium.prausnitzii_D	31.3	0.314	0.667	-0.993	1.621	0.6379	0.7893
Blautia_A.sp000433815	68.8	0.559	1.229	-1.851	2.968	0.6494	0.7967
UMGS1071.sp900542375	43.8	-0.231	0.537	-1.284	0.822	0.6672	0.8051
ER4.sp900317525	68.8	0.513	1.188	-1.816	2.843	0.6657	0.8051
Clostridium_Q.sp003024715	62.5	0.280	0.706	-1.103	1.664	0.6912	0.8272
Mediterraneibacter.lactaris	62.5	-0.457	1.177	-2.764	1.849	0.6976	0.8280
Faecalimonas.nexilis	62.5	-0.380	1.014	-2.367	1.607	0.7078	0.8306
Gemmiger.sp900539695	68.8	0.261	0.704	-1.118	1.639	0.7111	0.8306
KLE1615.sp900066985	43.8	-0.305	0.948	-2.164	1.553	0.7476	0.8595
Flavonifractor.plautii	12.5	0.188	0.582	-0.953	1.329	0.7469	0.8595
CAG.81.sp000435795	62.5	-0.268	0.884	-2.000	1.465	0.7619	0.8690
Blautia_A.sp900066355	56.3	0.149	0.529	-0.889	1.186	0.7790	0.8816
Agathobaculum.sp900291975	56.3	0.212	0.778	-1.313	1.737	0.7849	0.8816
Longicatena.caecimuris	62.5	-0.253	0.965	-2.145	1.638	0.7929	0.8837
Romboutsia.timonensis	56.3	0.157	0.773	-1.359	1.673	0.8393	0.9283
Alistipes_A.ihumii	62.5	-0.199	1.181	-2.514	2.117	0.8666	0.9306
Acetatifactor.sp900066365	56.3	-0.160	0.917	-1.957	1.638	0.8618	0.9306
Blautia_A.obeum	31.3	0.133	0.715	-1.268	1.534	0.8526	0.9306
Blautia_A.sp900066205	68.8	-0.082	0.491	-1.045	0.880	0.8669	0.9306
Escherichia.sp000208585	62.5	-0.130	0.860	-1.816	1.555	0.8794	0.9372
Bifidobacterium.pseudocatenulatum	56.3	0.176	1.476	-2.717	3.068	0.9052	0.9508

Evtepia.sp900546255	68.8	0.095	0.758	-1.390	1.581	0.8999	0.9508
Eggerthella.lenta	25.0	0.056	0.566	-1.054	1.166	0.9210	0.9604
Bacteroides.caccae	68.8	0.057	0.810	-1.531	1.645	0.9439	0.9774
Bacteroides.fragilis	56.3	0.034	1.263	-2.441	2.510	0.9783	0.9945
Alistipes.obesi	68.8	23.193	22605.061	-44282.727	44329.113	0.9992	0.9992
Alistipes.sp006542685	68.8	-3.156	355.626	-700.182	693.871	0.9929	0.9992
Gemmiger.formicilis	50.0	-0.013	0.537	-1.066	1.041	0.9809	0.9945
Ruthenibacterium.lactatiformans	31.3	-0.022	0.787	-1.564	1.520	0.9773	0.9945

Non-remission week 52

Species	ZP (%)	Coefficient	Std.error	Lower 95% CI	Higher 95% CI	p-Value	q-Value
Bacteroides.salyersiae	25.0	1.831	0.297	1.249	2.413	7.05E-10	1.24E-07
f_Bacteroidaceae.g_Phocaeicola.s_unknown	33.3	2.985	0.591	1.826	4.143	4.45E-07	3.91E-05
Ruminococcus_E.bromii_B	58.3	-1.126	0.230	-1.577	-0.676	9.60E-07	5.63E-05
Intestinibacter.bartlettii	58.3	2.271	0.481	1.328	3.215	2.37E-06	1.04E-04
Bifidobacterium.bifidum	58.3	2.492	0.625	1.267	3.717	6.68E-05	0.0024
Butyricimonas.faecihominis	41.7	3.104	0.835	1.468	4.741	2.01E-04	0.0059
Streptococcus.sp001556435	58.3	-1.786	0.493	-2.752	-0.820	2.90E-04	0.0073
Fusicatenibacter.saccharivorans	16.7	0.858	0.246	0.376	1.339	4.81E-04	0.0106
Alistipes.shahii	33.3	1.814	0.547	0.742	2.886	9.10E-04	0.0164
Parabacteroides.distasonis	25.0	2.200	0.670	0.887	3.512	0.0010	0.0164
Clostridium_Q.symbiosum	41.7	2.233	0.678	0.903	3.562	0.0010	0.0164
Blautia_A.wexlerae	41.7	2.307	0.722	0.892	3.721	0.0014	0.0204
Eisenbergiella.massiliensis	66.7	0.963	0.315	0.345	1.580	0.0022	0.0303
Eubacterium_I.ramulus_A	58.3	-1.985	0.658	-3.276	-0.695	0.0026	0.0323
UBA7182.sp003480725	50.0	1.937	0.673	0.617	3.256	0.0040	0.0471
Enterocloster.aldenensis	25.0	1.679	0.595	0.512	2.846	0.0048	0.0529
Blautia_A.sp900066355	25.0	-2.543	0.955	-4.414	-0.672	0.0077	0.0800
CAG.81.sp000435795	33.3	-2.130	0.841	-3.778	-0.482	0.0113	0.0974
Roseburia.intestinalis	41.7	1.055	0.415	0.242	1.869	0.0110	0.0974
Clostridium_A.leptum	50.0	-2.228	0.878	-3.948	-0.508	0.0111	0.0974
Agathobacter.faecis	25.0	-1.526	0.605	-2.712	-0.341	0.0116	0.0974
Ruthenibacterium.lactatiformans	8.3	-0.484	0.194	-0.864	-0.105	0.0123	0.0985
Paraprevotella.xylaniphila	50.0	0.774	0.326	0.134	1.414	0.0177	0.1304
Anaerotruncus.colihominis	25.0	1.361	0.574	0.236	2.487	0.0178	0.1304
Bacteroides.cellulosilyticus	33.3	1.863	0.807	0.282	3.444	0.0209	0.1417
Bacteroides.thetaiotaomicron	25.0	0.965	0.420	0.142	1.788	0.0215	0.1417
Odoribacter.splanchnicus	16.7	0.694	0.302	0.101	1.287	0.0217	0.1417
Dorea.sp900066555	66.7	-1.504	0.664	-2.804	-0.203	0.0235	0.1466
Ruminococcus_C.sp000980705	66.7	-5.046	2.238	-9.433	-0.659	0.0242	0.1466
Agathobacter.rectalis	50.0	-1.976	0.882	-3.705	-0.247	0.0251	0.1472
Akkermansia.muciniphila_B	66.7	-4.193	1.891	-7.900	-0.487	0.0266	0.1509
Mediterraneibacter.faecis	66.7	-2.357	1.077	-4.467	-0.247	0.0286	0.1525
Acidaminococcus.intestini	50.0	0.395	0.180	0.042	0.749	0.0283	0.1525
Bacteroides.caecimuris	50.0	3.846	1.770	0.377	7.315	0.0298	0.1529
Alistipes.onderdonkii	16.7	-2.423	1.127	-4.633	-0.213	0.0316	0.1529
Lachnospira.rogosae	66.7	2.334	1.084	0.209	4.458	0.0314	0.1529
Phocaeicola.vulgatus	0.0	1.266	0.593	0.104	2.427	0.0327	0.1529
Bacteroides.xylanisolvens	33.3	1.039	0.496	0.068	2.010	0.0360	0.1529
Enterocloster.citroniae	66.7	2.959	1.409	0.198	5.721	0.0357	0.1529
CAG.217.sp000436335	66.7	-1.673	0.794	-3.230	-0.117	0.0351	0.1529
Klebsiella.pneumoniae	58.3	2.579	1.230	0.167	4.991	0.0361	0.1529
UMGS1071.sp900542375	50.0	-1.353	0.647	-2.621	-0.085	0.0365	0.1529
Butyricimonas.virosa	66.7	2.809	1.428	0.010	5.607	0.0492	0.2013
f_Bacteroidaceae.g_Bacteroides.s_unknown	41.7	0.889	0.455	-0.003	1.780	0.0507	0.2028
Enterocloster.sp900541315	41.7	-1.251	0.650	-2.526	0.024	0.0544	0.2128
CAG.1427.sp000435675	66.7	-2.812	1.505	-5.761	0.137	0.0616	0.2308
UMGS1375.sp900066615	66.7	-2.063	1.101	-4.221	0.095	0.0610	0.2308
Clostridium_Q.sp003024715	33.3	-1.441	0.777	-2.965	0.083	0.0638	0.2338
Ruminococcus_C.callidus	66.7	2.957	1.612	-0.202	6.117	0.0666	0.2353
Blautia_A.sp900120195	50.0	-0.804	0.440	-1.667	0.058	0.0674	0.2353
Enterocloster.sp001517625	41.7	-2.031	1.114	-4.213	0.152	0.0682	0.2353
Alistipes_A.ihumii	33.3	1.080	0.611	-0.118	2.278	0.0772	0.2518
Eubacterium_F.sp003491505	66.7	-2.844	1.601	-5.982	0.294	0.0757	0.2518
f_Anaerovoracaceae.g_unknown.s_unknown	58.3	-1.765	0.998	-3.721	0.190	0.0768	0.2518
Escherichia.flexneri	25.0	-1.930	1.113	-4.111	0.251	0.0829	0.2653
CAG.194.sp000432915	66.7	1.525	0.888	-0.215	3.265	0.0858	0.2697
Dorea.sp000433535	50.0	0.783	0.463	-0.124	1.690	0.0905	0.2794
Bacteroides.eggerthii	58.3	-2.605	1.567	-5.676	0.466	0.0964	0.2874
Erysipelatoclostridium.ramosum	41.7	-1.404	0.844	-3.058	0.251	0.0963	0.2874
Fusobacterium_A.ulcerans	58.3	1.791	1.082	-0.330	3.912	0.0980	0.2874

Roseburia.inulinivorans	50.0	-0.924	0.570	-2.041	0.192	0.1047	0.3021
Bacteroides.stercoris	16.7	1.687	1.068	-0.406	3.781	0.1142	0.3243
f_Oscillospiraceae.g_CAG.103.s_unknown	58.3	-2.064	1.324	-4.660	0.532	0.1191	0.3275
Streptococcus.vestibularis	41.7	-1.153	0.739	-2.601	0.296	0.1189	0.3275
UMGS856.sp900546265	66.7	1.633	1.101	-0.526	3.792	0.1382	0.3742
CAG.180.sp000432435	66.7	1.895	1.298	-0.648	4.438	0.1442	0.3845
Enterocloster.clostridioformis	25.0	-1.272	0.890	-3.017	0.473	0.1532	0.4025
f_Oscillospiraceae.g_Oscillibacter.s_unknown	50.0	1.163	0.828	-0.461	2.787	0.1603	0.4150
Mediterraneibacter_A.butyricigenes	66.7	1.836	1.324	-0.760	4.431	0.1657	0.4226
Lachnospira.sp000437735	66.7	-1.954	1.420	-4.738	0.830	0.1689	0.4247
Lawsonibacter.asaccharolyticus	25.0	0.718	0.539	-0.338	1.774	0.1826	0.4510
Bacteroides.fragilis	66.7	-1.672	1.267	-4.155	0.812	0.1870	0.4510
Anaerotignum.sp000436415	66.7	-0.445	0.337	-1.106	0.216	0.1869	0.4510
Bacteroides.clarus	66.7	-1.251	1.003	-3.218	0.715	0.2124	0.5025
Hungatella.sp005845265	58.3	0.954	0.774	-0.563	2.470	0.2177	0.5025
Bacteroides.caccae	16.7	0.336	0.274	-0.202	0.874	0.2208	0.5025
CAG.45.sp900066395	58.3	-0.836	0.684	-2.176	0.504	0.2216	0.5025
Ruminococcus_H.sp003531055	58.3	-0.732	0.601	-1.910	0.445	0.2227	0.5025
Bilophila.wadsworthia	41.7	-0.705	0.584	-1.850	0.439	0.2271	0.5060
Enterocloster.sp000155435	66.7	-1.051	0.877	-2.769	0.667	0.2306	0.5073
Blautia_A.sp000285855	41.7	0.607	0.521	-0.414	1.628	0.2440	0.5238
Blautia_A.sp900066335	50.0	1.362	1.198	-0.987	3.710	0.2558	0.5257
Dorea.formicigenerans	25.0	-0.534	0.455	-1.426	0.357	0.2401	0.5217
Faecalimonas.umbilicata	41.7	1.293	1.130	-0.922	3.507	0.2526	0.5257
f_Lachnospiraceae.g_UBA7160.s_unknown	58.3	-0.513	0.451	-1.397	0.372	0.2560	0.5257
Streptococcus.thermophilus	33.3	-1.288	1.136	-3.515	0.939	0.2569	0.5257
Blautia_A.obeum	16.7	-0.658	0.591	-1.816	0.499	0.2650	0.5309
Phocaeicola.massiliensis	50.0	0.584	0.528	-0.451	1.619	0.2691	0.5309
Coprococcus_A.catus	58.3	-0.923	0.839	-2.566	0.721	0.2712	0.5309
Ruminococcus_E.sp003526955	66.7	-2.192	1.993	-6.099	1.715	0.2715	0.5309
Dorea.longicatena	50.0	-0.756	0.693	-2.114	0.602	0.2751	0.5321
Bifidobacterium.longum	41.7	0.291	0.277	-0.252	0.834	0.2932	0.5324
Alistipes.putredinis	16.7	0.769	0.720	-0.643	2.180	0.2857	0.5324
Dorea.sp000433215	41.7	-0.695	0.657	-1.983	0.592	0.2900	0.5324
Phascolarctobacterium.faecium	58.3	1.142	1.065	-0.945	3.230	0.2834	0.5324
Parasutterella.excrementihominis	66.7	1.610	1.526	-1.381	4.602	0.2915	0.5324
Bacteroides.ovatus	33.3	1.293	1.241	-1.139	3.726	0.2973	0.5324
Faecalibacterium.prausnitzii_D	58.3	1.538	1.477	-1.356	4.433	0.2976	0.5324
CAG.103.sp900317855	50.0	-0.973	0.944	-2.823	0.877	0.3025	0.5324
Oscillibacter.welbionis	33.3	0.727	0.704	-0.652	2.106	0.3017	0.5324
CAG.103.sp000432375	41.7	1.062	1.080	-1.055	3.180	0.3254	0.5670
f_Burkholderiaceae.g_Sutterella.s_unknown	66.7	1.312	1.349	-1.331	3.956	0.3306	0.5705
Dorea.sp000509125	66.7	-1.980	2.127	-6.148	2.188	0.3518	0.5904
Mediterraneibacter.torques	25.0	-0.493	0.534	-1.539	0.553	0.3554	0.5904
f_Lachnospiraceae.g_Mediterraneibacter.s_unknown	41.7	1.009	1.098	-1.142	3.161	0.3579	0.5904
Roseburia.hominis	41.7	-0.409	0.431	-1.253	0.435	0.3422	0.5847
Agathobaculum.sp900291975	66.7	-0.960	1.046	-3.011	1.091	0.3589	0.5904
Akkermansia.muciniphila	66.7	-0.878	0.982	-2.803	1.047	0.3713	0.6051
Gemmiger.formicilis	66.7	0.729	0.824	-0.885	2.343	0.3759	0.6070
Prevotella.copri	58.3	1.121	1.278	-1.384	3.627	0.3804	0.6086
Paraprevotella.clara	58.3	1.046	1.241	-1.387	3.480	0.3993	0.6164
Alistipes.finegoldii	25.0	0.556	0.645	-0.708	1.820	0.3888	0.6117
CAG.81.sp900066535	33.3	0.630	0.743	-0.827	2.087	0.3969	0.6164
Gemmiger.qucibialis	25.0	0.511	0.594	-0.653	1.675	0.3893	0.6117
Alistipes_A.indistinctus	33.3	-0.684	0.818	-2.286	0.918	0.4027	0.6164
Anaerobutyricum.hallii	50.0	-0.522	0.633	-1.762	0.717	0.4089	0.6204
Blautia.coccoides	58.3	-0.880	1.094	-3.024	1.263	0.4210	0.6333
Ruminococcus_B.gnavus	16.7	0.460	0.586	-0.688	1.608	0.4324	0.6449
Eisenbergiella.tayi	58.3	0.551	0.775	-0.968	2.069	0.4772	0.7057
Bariatricus.comes	41.7	-0.432	0.650	-1.706	0.841	0.5058	0.7305
Hungatella.effluvii	58.3	-0.446	0.674	-1.766	0.874	0.5076	0.7305
Bacteroides.faecis	58.3	0.535	0.819	-1.071	2.141	0.5135	0.7305
UBA1191.sp900549125	58.3	-0.476	0.730	-1.908	0.955	0.5142	0.7305
CAG.83.sp000431575	41.7	0.447	0.688	-0.901	1.795	0.5158	0.7305
Anaeromassilibacillus.sp002159845	58.3	-0.459	0.712	-1.855	0.936	0.5188	0.7305
Collinsella.intestinalis	66.7	-0.546	0.867	-2.246	1.154	0.5292	0.7310
UBA11774.sp003507655	41.7	0.573	0.916	-1.223	2.369	0.5316	0.7310
Ruminiclostridium_E.siraeum	66.7	0.764	1.216	-1.620	3.148	0.5301	0.7310
Acetatifactor.sp900066565	25.0	0.519	0.868	-1.183	2.221	0.5502	0.7449
Blautia_A.sp900066145	41.7	-0.179	0.296	-0.760	0.402	0.5456	0.7444
Eggerthella.lenta	50.0	0.291	0.540	-0.767	1.350	0.5897	0.7923

Eisenbergiella.sp900066775	33.3	0.224	0.431	-0.620	1.069	0.6026	0.7997
Blautia_A.sp900066165	25.0	0.476	0.918	-1.323	2.274	0.6043	0.7997
Barnesiella.intestinihominis	50.0	-0.628	1.262	-3.102	1.846	0.6188	0.8128
Streptococcus.salivarius	50.0	-0.369	0.756	-1.851	1.112	0.6251	0.8150
Enterocloster.sp000431375	58.3	0.409	0.932	-1.417	2.235	0.6605	0.8548
Gordonibacter.urolithinfaciens	58.3	-0.296	0.754	-1.774	1.182	0.6944	0.8824
Agathobaculum.butyriciproducens	41.7	-0.171	0.438	-1.029	0.688	0.6969	0.8824
UBA1191.sp900543485	66.7	0.419	1.029	-1.598	2.436	0.6839	0.8785
Harryflintia.acetispora	58.3	0.260	0.690	-1.093	1.613	0.7062	0.8878
Dialister.invisus	66.7	-0.384	1.067	-2.475	1.708	0.7192	0.8914
f_Dialisteraceae.g_Dialister.s_unknown	66.7	-0.410	1.115	-2.596	1.776	0.7131	0.8902
Parabacteroides.merdae	33.3	-0.259	0.766	-1.760	1.241	0.7350	0.9032
Acetatifactor.sp900066365	66.7	0.390	1.180	-1.923	2.703	0.7411	0.9032
Longicatena.innocuum	16.7	0.159	0.488	-0.797	1.115	0.7441	0.9032
SFFH01.sp900542445	58.3	0.259	0.868	-1.442	1.960	0.7652	0.9224
Flavonifractor.plautii	8.3	-0.173	0.594	-1.338	0.991	0.7708	0.9225
Alistipes.obesi	50.0	0.355	1.279	-2.150	2.861	0.7810	0.9225
Eubacterium_G.ventriosum	58.3	0.385	1.367	-2.294	3.064	0.7782	0.9225
Blautia_A.massiliensis	16.7	-0.196	0.729	-1.624	1.233	0.7882	0.9249
Blautia_A.sp003477525	66.7	0.329	1.280	-2.180	2.837	0.7974	0.9281
Senegalimassilia.anaerobia	66.7	-0.246	1.063	-2.329	1.838	0.8173	0.9281
Enterocloster.lavalensis	66.7	0.215	0.913	-1.574	2.003	0.8141	0.9281
Lachnospira.eligens_B	66.7	-0.550	2.358	-5.170	4.071	0.8156	0.9281
Lactococcus.lactis	66.7	-0.215	0.903	-1.985	1.555	0.8116	0.9281
Faecalibacterium.prausnitzii_G	66.7	-0.419	1.886	-4.116	3.277	0.8240	0.9296
CAG.170.sp000432135	66.7	-0.485	2.407	-5.203	4.233	0.8404	0.9421
KLE1615.sp900066985	58.3	-0.216	1.139	-2.449	2.017	0.8495	0.9463
Anaerostipes.hadrus	16.7	-0.104	0.671	-1.420	1.211	0.8763	0.9632
CAG.81.sp900066785	58.3	0.109	0.691	-1.245	1.463	0.8748	0.9632
Enterocloster.bolteae	16.7	0.094	0.645	-1.169	1.358	0.8837	0.9632
Bacteroides.uniformis	16.7	0.080	0.602	-1.101	1.260	0.8946	0.9632
Anaerobutyricum.hallii_A	25.0	-0.055	0.451	-0.940	0.829	0.9026	0.9632
Muricomes.sp000509105	66.7	-0.137	1.121	-2.334	2.060	0.9028	0.9632
Oscillibacter.sp900542115	66.7	0.123	1.006	-1.850	2.095	0.9030	0.9632
f_Lachnospiraceae.g_Blautia_A.s_unknown	50.0	0.104	0.985	-1.827	2.036	0.9156	0.9707
Blautia_A.sp000433815	50.0	-0.086	0.875	-1.800	1.629	0.9220	0.9716
Adlercreutzia.equolifaciens	66.7	-0.066	1.031	-2.086	1.955	0.9492	0.9844
Ruminococcus_A.sp000437095	66.7	0.077	1.169	-2.215	2.369	0.9474	0.9844
Romboutsia.timonensis	66.7	-0.061	0.992	-2.006	1.884	0.9509	0.9844
Blautia_A.hydrogenotrophica	58.3	0.027	0.759	-1.460	1.515	0.9714	0.9882
CAG.110.sp003525905	58.3	-0.037	0.953	-1.905	1.831	0.9689	0.9882
ER4.sp900317525	66.7	-0.106	2.021	-4.067	3.856	0.9583	0.9864
f.Lachnospirales_g.Lachnospiraceae_KLE1615.s_unknown	66.7	-0.019	1.091	-2.157	2.119	0.9863	0.9976
Parabacteroides.johnsonii	58.3	0.004	0.705	-1.378	1.385	0.9960	0.9977
ER4.sp000765235	66.7	0.004	1.476	-2.889	2.898	0.9977	0.9977
