# Supplementary material for: Phage-Display Immunoprecipitation Sequencing Reveals Distinct Antibody Signatures Against Bacterial Flagellins Associated With Treatment Response in Crohn's Disease
Source: Clin Transl Gastroenterol. 2026 Apr 2;17(6):e01030. doi: 10.14309/ctg.0000000000001030 (PMC13641668; doi:10.14309/ctg.0000000000001030)
Supplement: Supplementary file 5 [file ct9-17-e01030-s005.pdf]

Supplementary Table 5

Remission week 14

Pathway	ZP (%)	Coefficient	Std.error	Lower 95% CI	Higher 95% CI	p-Value	q-Value
PWY.7328..superpathway.of.UDP.glucose.derived.O.antigen.building.blocks.biosynthesis	0.0	-0.346	0.092	-0.526	-0.166	1.62E-04	0.0187
PWY.7111..pyruvate.fermentation.to.isobutanol..engineered.	0.0	-0.378	0.111	-0.596	-0.160	6.89E-04	0.0396
PWY.6121..5.aminoimidazole.ribonucleotide.biosynthesis.I	0.0	-0.146	0.046	-0.237	-0.055	0.0017	0.0660
P441.PWY..superpathway.of.N.acetylneuraminate.degradation	6.7	-0.574	0.207	-0.980	-0.168	0.0056	0.1294
PWY.6936..seleno.amino.acid.biosynthesis..plants.	3.3	-0.456	0.164	-0.777	-0.134	0.0055	0.1294
PWY0.1477..ethanolamine.utilization	3.3	-0.886	0.328	-1.528	-0.243	0.0069	0.1320
DAPLYSINESYN.PWY..L.lysine.biosynthesis.I	13.3	-0.537	0.223	-0.974	-0.100	0.0161	0.1322
FERMENTATION.PWY..mixed.acid.fermentation	10.0	-0.517	0.220	-0.949	-0.085	0.0190	0.1322
HISDEG.PWY..L.histidine.degradation.I	0.0	0.415	0.180	0.063	0.767	0.0208	0.1322
HSERMETANA.PWY..L.methionine.biosynthesis.III	0.0	-0.253	0.106	-0.461	-0.045	0.0173	0.1322
ILEUSYN.PWY..L.isoleucine.biosynthesis.I..from.threonine.	0.0	-0.139	0.060	-0.256	-0.021	0.0205	0.1322
PWY.2942..L.lysine.biosynthesis.III	0.0	-0.115	0.051	-0.215	-0.015	0.0236	0.1322
PWY.6353..purine.nucleotides.degradation.II..aerobic.	3.3	-0.486	0.208	-0.894	-0.077	0.0198	0.1322
PWY.6608..guanosine.nucleotides.degradation.III	0.0	-0.506	0.221	-0.939	-0.072	0.0222	0.1322
PWY.724..superpathway.of.L.lysine..L.threonine.and.L.methionine.biosynthesis.II	0.0	-0.122	0.053	-0.226	-0.019	0.0207	0.1322
PWY.7357..thiamine.phosphate.formation.from.pyrithiamine.and.oxythiamine..yeast.	0.0	-0.164	0.068	-0.297	-0.030	0.0163	0.1322
PWY.8178..pentose.phosphate.pathway..non.oxidative.branch..II	0.0	-0.216	0.092	-0.396	-0.035	0.0192	0.1322
PWY.I9..L.cysteine.biosynthesis.VI..from.L.methionine.	0.0	-0.654	0.285	-1.213	-0.095	0.0218	0.1322
PWY66.409..superpathway.of.purine.nucleotide.salvage	13.3	-0.633	0.280	-1.182	-0.084	0.0239	0.1322
SALVADEHYPOX.PWY..adenosine.nucleotides.degradation.II	3.3	-0.671	0.298	-1.255	-0.087	0.0242	0.1322
NONOXIPENT.PWY..pentose.phosphate.pathway..non.oxidative.branch..I	0.0	-0.268	0.120	-0.503	-0.033	0.0253	0.1322
FASYN.ELONG.PWY..fatty.acid.elongation....saturated	6.7	-0.369	0.172	-0.706	-0.033	0.0315	0.1430
PWY.5345..superpathway.of.L.methionine.biosynthesis..by.sulfhydrylation.	23.3	-0.681	0.320	-1.309	-0.053	0.0336	0.1430
PWY.6628..superpathway.of.L.phenylalanine.biosynthesis	3.3	-0.402	0.189	-0.773	-0.032	0.0334	0.1430
PWY.6630..superpathway.of.L.tyrosine.biosynthesis	3.3	-0.516	0.236	-0.979	-0.053	0.0290	0.1430
PWY.7400..L.arginine.biosynthesis.IV..archaebacteria.	0.0	-0.253	0.119	-0.485	-0.021	0.0327	0.1430
ARGSYN.PWY..L.arginine.biosynthesis.I..via.L.ornithine.	0.0	-0.241	0.115	-0.466	-0.016	0.0357	0.1449
PWY.6282..palmitoleate.biosynthesis.I..from..5Z..dodec.5.enoate.	6.7	-0.410	0.197	-0.796	-0.024	0.0375	0.1449
PWY.7761..NAD.salvage.pathway.II..PNC.IV.cycle.	0.0	-0.463	0.223	-0.901	-0.026	0.0380	0.1449
PWY.7664..oleate.biosynthesis.IV..anaerobic.	6.7	-0.390	0.189	-0.760	-0.020	0.0391	0.1449
ARGININE.SYN4.PWY..L.ornithine.biosynthesis.II	0.0	0.337	0.164	0.014	0.659	0.0406	0.1458
P41.PWY..pyruvate.fermentation.to.acetate.and..S..lactate.I	0.0	-0.365	0.180	-0.718	-0.013	0.0423	0.1474
BRANCHED.CHAIN.AA.SYN.PWY..superpathway.of.branched.chain.amino.acid.biosynthesis	0.0	-0.124	0.062	-0.246	-0.001	0.0474	0.1474
PWY.5659..GDP.mannose.biosynthesis	0.0	0.204	0.102	0.003	0.404	0.0463	0.1474
PWY.5989..stearate.biosynthesis.II..bacteria.and.plants.	6.7	-0.359	0.180	-0.712	-0.005	0.0466	0.1474
PWY.7117..C4.photosynthetic.carbon.assimilation.cycle..PEPCK.type	10.0	-0.511	0.254	-1.008	-0.014	0.0439	0.1474
SULFATE.CYS.PWY..superpathway.of.sulfate.assimilation.and.cysteine.biosynthesis	20.0	-0.575	0.308	-1.179	0.030	0.0623	0.1885
PWY.3001..superpathway.of.L.isoleucine.biosynthesis.I	0.0	-0.183	0.099	-0.378	0.012	0.0653	0.1925
PWY0.862...5Z..dodecenoate.biosynthesis.I	6.7	-0.378	0.211	-0.792	0.037	0.0740	0.2127
PWY.7228..superpathway.of.guanosine.nucleotides.de.novo.biosynthesis.I	0.0	0.176	0.100	-0.020	0.372	0.0782	0.2193
GLYCOLYSIS..glycolysis.I..from.glucose.6.phosphate.	0.0	-0.146	0.084	-0.311	0.018	0.0815	0.2230
PWY.6125..superpathway.of.guanosine.nucleotides.de.novo.biosynthesis.II	0.0	0.206	0.120	-0.029	0.442	0.0860	0.2299
PWY.2941..L.lysine.biosynthesis.II	0.0	0.176	0.109	-0.036	0.389	0.1041	0.2483
PWY.4041...gamma..glutamyl.cycle	3.3	-0.357	0.218	-0.783	0.070	0.1012	0.2483
PWY.6731..starch.degradation.III	0.0	0.348	0.213	-0.069	0.765	0.1017	0.2483
PWY.7220..adenosine.deoxyribonucleotides.de.novo.biosynthesis.II	0.0	0.262	0.158	-0.047	0.571	0.0969	0.2476
PWY.7222..guanosine.deoxyribonucleotides.de.novo.biosynthesis.II	0.0	0.262	0.158	-0.047	0.571	0.0969	0.2476
PWY.7282..4.amino.2.methyl.5.diphosphomethylpyrimidine.biosynthesis.II	0.0	0.186	0.115	-0.039	0.411	0.1058	0.2483
ARG.POLYAMINE.SYN..superpathway.of.arginine.and.polyamine.biosynthesis	3.3	-0.384	0.242	-0.859	0.091	0.1127	0.2593
COA.PWY..coenzyme.A.biosynthesis.I..prokaryotic.	0.0	-0.087	0.057	-0.198	0.025	0.1274	0.2764
PWY.5030..L.histidine.degradation.III	16.7	0.489	0.315	-0.129	1.108	0.1210	0.2676
PWY.5971..palmitate.biosynthesis..type.II.fatty.acid.synthase.	33.3	-0.622	0.413	-1.431	0.188	0.1322	0.2814
PWY.6126..superpathway.of.adenosine.nucleotides.de.novo.biosynthesis.II	0.0	0.168	0.108	-0.044	0.379	0.1210	0.2676
ANAEROFRUCAT.PWY..homolactic.fermentation	0.0	-0.112	0.076	-0.261	0.036	0.1381	0.2835
PWY.6703..preQ0.biosynthesis	0.0	0.171	0.115	-0.054	0.396	0.1373	0.2835
PWY.6519..8.amino.7.oxononanoate.biosynthesis.I	6.7	-0.233	0.159	-0.546	0.079	0.1434	0.2894
PWY.5973..cis.vaccenate.biosynthesis	0.0	0.146	0.105	-0.060	0.352	0.1649	0.3179
PWY.6305..superpathway.of.putrescine.biosynthesis	3.3	-0.258	0.187	-0.624	0.108	0.1672	0.3179
PWY.7234..inosine.5..phosphate.biosynthesis.III	6.7	-0.313	0.226	-0.755	0.129	0.1657	0.3179
PWY.7323..superpathway.of.GDP.mannose.derived.O.antigen.building.blocks.biosynthesis	3.3	0.207	0.151	-0.088	0.502	0.1686	0.3179
THRESYN.PWY..superpathway.of.L.threonine.biosynthesis	0.0	-0.074	0.056	-0.183	0.035	0.1850	0.3432
PWY.6317..D.galactose.degradation.I..Leloir.pathway.	0.0	-0.163	0.124	-0.406	0.080	0.1894	0.3458
GLUCONEO.PWY..gluconeogenesis.I	3.3	0.197	0.154	-0.106	0.499	0.2023	0.3579
PWY.7229..superpathway.of.adenosine.nucleotides.de.novo.biosynthesis.I	0.0	0.111	0.087	-0.060	0.282	0.2020	0.3579
PWY.5941..glycogen.degradation.II	0.0	-0.162	0.130	-0.416	0.093	0.2132	0.3714
THISYNARA.PWY..superpathway.of.thiamine.diphosphate.biosynthesis.III..eukaryotes.	0.0	-0.097	0.079	-0.251	0.057	0.2177	0.3736
GLUCOSE1PMETAB.PWY..glucose.and.glucose.1.phosphate.degradation	16.7	-0.323	0.274	-0.860	0.214	0.2389	0.4041
RIBOSYN2.PWY..flavin.biosynthesis.I..bacteria.and.plants.	3.3	0.158	0.140	-0.116	0.433	0.2584	0.4307
PWY.7663..gondoate.biosynthesis..anaerobic.	0.0	0.093	0.085	-0.073	0.259	0.2699	0.4371
PWY.841..superpathway.of.purine.nucleotides.de.novo.biosynthesis.I	0.0	0.066	0.059	-0.050	0.182	0.2644	0.4344
BIOTIN.BIOSYNTHESIS.PWY..biotin.biosynthesis.I	6.7	-0.177	0.165	-0.500	0.146	0.2816	0.4498
POLYISOPRENSYN.PWY..polyisoprenoid.biosynthesis..E..coli.	3.3	-0.151	0.151	-0.447	0.144	0.3161	0.4912
PWY.4984..urea.cycle	16.7	0.303	0.302	-0.289	0.896	0.3156	0.4912
PWY.5484..glycolysis.II..from.fructose.6.phosphate.	0.0	-0.078	0.079	-0.234	0.077	0.3236	0.4961
PWY.6124..inosine.5..phosphate.biosynthesis.II	0.0	0.078	0.080	-0.079	0.236	0.3288	0.4976
PWY.5686..UMP.biosynthesis.I	0.0	-0.050	0.053	-0.154	0.055	0.3506	0.5108
PWY.6895..superpathway.of.thiamine.diphosphate.biosynthesis.II	10.0	-0.217	0.228	-0.663	0.230	0.3413	0.5097
SER.GLYSYN.PWY..superpathway.of.L.serine.and.glycine.biosynthesis.I	0.0	-0.063	0.068	-0.196	0.070	0.3509	0.5108

PWY.6123..inosine.5..phosphate.biosynthesis.I	0.0	0.057	0.068	-0.077	0.190	0.4061	0.5837
CITRULBIO.PWY..L.citrulline.biosynthesis	3.3	0.105	0.131	-0.151	0.361	0.4213	0.5902
PWY.6168..flavin.biosynthesis.III..fungi.	13.3	-0.159	0.199	-0.549	0.231	0.4248	0.5902
PWY.7208..superpathway.of.pyrimidine.nucleobases.salvage	0.0	0.078	0.098	-0.114	0.271	0.4260	0.5902
PWY.7790..UMP.biosynthesis.II	0.0	-0.041	0.053	-0.145	0.063	0.4394	0.5944
PWY.7791..UMP.biosynthesis.III	0.0	-0.041	0.053	-0.145	0.063	0.4394	0.5944
PWY.7197..pyrimidine.deoxyribonucleotide.phosphorylation	0.0	-0.085	0.121	-0.322	0.152	0.4821	0.6442
PWY.8187..L.arginine.degradation.XIII..reductive.Stickland.reaction.	3.3	-0.115	0.166	-0.439	0.209	0.4873	0.6442
PENTOSE.P.PWY..pentose.phosphate.pathway	0.0	-0.055	0.086	-0.222	0.113	0.5222	0.6825
GLYCOCAT.PWY..glycogen.degradation.I	0.0	-0.131	0.215	-0.552	0.290	0.5416	0.6999
PWY.7221..guanosine.ribonucleotides.de.novo.biosynthesis	0.0	-0.026	0.045	-0.114	0.061	0.5569	0.7115
PWY0.1586..peptidoglycan.maturation..meso.diaminopimelate.containing.	0.0	-0.051	0.090	-0.229	0.126	0.5704	0.7208
NAGLIPASYN.PWY..lipid.IVA.biosynthesis..E..coli.	0.0	0.118	0.218	-0.310	0.546	0.5902	0.7298
PWY.6897..thiamine.diphosphate.salvage.II	0.0	0.036	0.069	-0.099	0.171	0.5971	0.7305
PWY.8073..lipid.IVA.biosynthesis..P..putida.	0.0	0.118	0.218	-0.310	0.546	0.5902	0.7298
PWY.5384..sucrose.degradation.IV..sucrose.phosphorylase.	0.0	-0.081	0.167	-0.409	0.247	0.6267	0.7556
PHOSLIPSYN.PWY..superpathway.of.phospholipid.biosynthesis.I..bacteria.	0.0	-0.053	0.112	-0.272	0.166	0.6373	0.7556
PWY.6270..isoprene.biosynthesis.I	3.3	-0.057	0.118	-0.288	0.175	0.6315	0.7556
GLYCOLYSIS.E.D..superpathway.of.glycolysis.and.the.Entner.Doudoroff.pathway	3.3	-0.089	0.195	-0.471	0.294	0.6490	0.7616
PWY.7560..methylerythritol.phosphate.pathway.II	3.3	-0.053	0.122	-0.292	0.185	0.6605	0.7672
PWY0.162..superpathway.of.pyrimidine.ribonucleotides.de.novo.biosynthesis	3.3	0.060	0.159	-0.252	0.372	0.7063	0.8122
RHAMCAT.PWY..L.rhamnose.degradation.I	0.0	0.039	0.107	-0.171	0.249	0.7176	0.8171
GLCMANNANAUT.PWY..superpathway.of.N.acetylglucosamine..N.acetylmannosamine.and.N.acetylneu	0.0	-0.055	0.155	-0.359	0.250	0.7247	0.8171
PWY.7392..taxadiene.biosynthesis..engineered.	3.3	-0.077	0.260	-0.586	0.432	0.7667	0.8560
COBALSYN.PWY..superpathway.of.adenosylcobalamin.salvage.from.cobinamide.I	0.0	-0.027	0.170	-0.360	0.307	0.8754	0.9587
PWY.621..sucrose.degradation.III..sucrose.invertase.	0.0	0.023	0.147	-0.265	0.312	0.8734	0.9587
PWY.8004..Entner.Doudoroff.pathway.I	3.3	-0.018	0.180	-0.371	0.336	0.9224	0.9841
PWY0.1261..anhydromuropeptides.recycling.I	0.0	0.019	0.202	-0.376	0.414	0.9261	0.9841
PYRIDNUCSYN.PWY..NAD.de.novo.biosynthesis.I..from.aspartate.	0.0	-0.005	0.052	-0.107	0.098	0.9298	0.9841
ASPASN.PWY..superpathway.of.L.aspartate.and.L.asparagine.biosynthesis	0.0	-0.005	0.070	-0.142	0.132	0.9413	0.9841
PWY.6901..superpathway.of.glucose.and.xylose.degradation	3.3	-0.009	0.123	-0.251	0.232	0.9391	0.9841
PWY.3841..folate.transformations.II..plants.	0.0	0.003	0.057	-0.108	0.114	0.9541	0.9844
PWY.6902..chitin.degradation.II..Vibrio.	0.0	0.009	0.231	-0.443	0.462	0.9672	0.9844
PWY.7345..superpathway.of.anaerobic.sucrose.degradation	3.3	-0.007	0.167	-0.333	0.320	0.9673	0.9844
GLUCUROCAT.PWY..superpathway.of..beta..D.glucuronosides.degradation	3.3	0.003	0.175	-0.340	0.346	0.9857	0.9857
PWY.5121..superpathway.of.geranylgeranyl.diphosphate.biosynthesis.II..via.MEP.	3.3	0.004	0.185	-0.358	0.366	0.9840	0.9857

Non-remission week 14

Pathway	ZP (%)	Coefficient	Std.error	Lower 95% CI	Higher 95% CI	p-Value	q-Value
PWY.7400..L.arginine.biosynthesis.IV..archaebacteria.	0.0	-0.285	0.063	-0.409	-0.162	5.95E-06	0.001
GLUTORN.PWY..L.ornithine.biosynthesis.I	0.0	-0.405	0.108	-0.616	-0.194	1.69E-04	0.013
NONOXIPENT.PWY..pentose.phosphate.pathway..non.oxidative.branch..I	0.0	-0.311	0.092	-0.491	-0.130	0.001	0.027
PWY.8178..pentose.phosphate.pathway..non.oxidative.branch..II	0.0	-0.229	0.068	-0.362	-0.096	0.001	0.027
ARGSYN.PWY..L.arginine.biosynthesis.I..via.L.ornithine.	0.0	-0.321	0.106	-0.528	-0.113	0.002	0.053
ARGSYNBSUB.PWY..L.arginine.biosynthesis.II..acetyl.cycle.	0.0	-0.381	0.130	-0.636	-0.126	0.003	0.063
PWY.5188..tetrapyrrole.biosynthesis.I..from.glutamate.	0.0	-0.561	0.180	-0.915	-0.208	0.002	0.053
PWY.6317..D.galactose.degradation.I..Leloir.pathway.	0.0	-0.292	0.101	-0.490	-0.094	0.004	0.063
GLYCOGENSYNTH.PWY..glycogen.biosynthesis.I..from.ADP.D.Glucose.	0.0	-0.253	0.090	-0.429	-0.077	0.005	0.072
PWY.6527..stachyose.degradation	0.0	-0.365	0.133	-0.626	-0.104	0.006	0.082
PWY.5941..glycogen.degradation.II	0.0	-0.192	0.071	-0.331	-0.053	0.007	0.085
PWY.6823..molybdopterin.biosynthesis	0.0	-0.335	0.126	-0.582	-0.088	0.008	0.090
P41.PWY..pyruvate.fermentation.to.acetate.and..S..lactate.I	0.0	-0.337	0.133	-0.597	-0.077	0.011	0.109
PWY.7238..sucrose.biosynthesis.II	0.0	-0.169	0.066	-0.299	-0.039	0.011	0.109
PWY0.1296..purine.ribonucleosides.degradation	0.0	-0.244	0.099	-0.437	-0.051	0.013	0.123
PWY.5100..pyruvate.fermentation.to.acetate.and.lactate.II	0.0	-0.304	0.125	-0.548	-0.059	0.015	0.131
COMPLETE.ARO.PWY..superpathway.of.aromatic.amino.acid.biosynthesis	0.0	-0.113	0.047	-0.206	-0.021	0.016	0.135
PWY.6608..guanosine.nucleotides.degradation.III	0.0	-0.533	0.230	-0.985	-0.081	0.021	0.155
UDPNAGSYN.PWY..UDP.N.acetyl.D.glucosamine.biosynthesis.I	0.0	-0.400	0.173	-0.738	-0.061	0.021	0.155
CALVIN.PWY..Calvin.Benson.Bassham.cycle	0.0	-0.147	0.066	-0.276	-0.018	0.026	0.184
PWY.6936..seleno.amino.acid.biosynthesis..plants.	0.0	-0.283	0.131	-0.540	-0.025	0.031	0.212
ARO.PWY..chorismate.biosynthesis.I	0.0	-0.132	0.063	-0.254	-0.009	0.036	0.228
PWY.5384..sucrose.degradation.IV..sucrose.phosphorylase.	0.0	-0.297	0.147	-0.584	-0.010	0.043	0.228
PWY.6122..5.aminoimidazole.ribonucleotide.biosynthesis.II	0.0	-0.104	0.051	-0.205	-0.003	0.043	0.228
PWY.6163..chorismate.biosynthesis.from.3.dehydroquinate	0.0	-0.121	0.059	-0.236	-0.005	0.041	0.228
PWY.6277..superpathway.of.5.aminoimidazole.ribonucleotide.biosynthesis	0.0	-0.104	0.051	-0.205	-0.003	0.043	0.228
PWY.6731..starch.degradation.III	0.0	-0.253	0.122	-0.493	-0.014	0.038	0.228
SALVADEHYPOX.PWY..adenosine.nucleotides.degradation.II	0.0	-0.626	0.314	-1.241	-0.011	0.046	0.237
PWY.6353..purine.nucleotides.degradation.II..aerobic.	3.3	-0.399	0.204	-0.799	0.000	0.050	0.249
ILEUSYN.PWY..L.isoleucine.biosynthesis.I..from.threonine.	0.0	-0.123	0.064	-0.247	0.002	0.053	0.254
PWY.6121..5.aminoimidazole.ribonucleotide.biosynthesis.I	0.0	-0.111	0.059	-0.227	0.006	0.063	0.293
RHAMCAT.PWY..L.rhamnose.degradation.I	0.0	0.244	0.138	-0.026	0.514	0.077	0.345
COLANSYN.PWY..colanic.acid.building.blocks.biosynthesis	0.0	-0.137	0.086	-0.305	0.031	0.109	0.391
HISDEG.PWY..L.histidine.degradation.I	0.0	0.295	0.179	-0.057	0.646	0.100	0.391
HSERMETANA.PWY..L.methionine.biosynthesis.III	0.0	-0.247	0.156	-0.553	0.058	0.113	0.391
OANTIGEN.PWY..O.antigen.building.blocks.biosynthesis..E..coli.	0.0	-0.182	0.112	-0.402	0.038	0.106	0.391
PWY.3001..superpathway.of.L.isoleucine.biosynthesis.I	0.0	-0.079	0.050	-0.177	0.019	0.113	0.391
PWY.5097..L.lysine.biosynthesis.VI	0.0	-0.115	0.067	-0.246	0.016	0.086	0.365
PWY.724..superpathway.of.L.lysine..L.threonine.and.L.methionine.biosynthesis.II	0.0	-0.087	0.054	-0.192	0.018	0.105	0.391
PWY4FS.7..phosphatidylglycerol.biosynthesis.I..plastidic.	0.0	-0.297	0.188	-0.665	0.070	0.113	0.391
PWY4FS.8..phosphatidylglycerol.biosynthesis.II..non.plastidic.	0.0	-0.297	0.188	-0.665	0.070	0.113	0.391
VALSYN.PWY..L.valine.biosynthesis	0.0	-0.094	0.054	-0.201	0.012	0.082	0.361
PWY.5030..L.histidine.degradation.III	0.0	0.237	0.152	-0.061	0.535	0.119	0.395

PWY.5154..L.arginine.biosynthesis.III..via.N.acetyl.L.citrulline.	0.0	-0.135	0.087	-0.306	0.035	0.120	0.395
PWY.6386..UDP.N.acetylmuramoyl.pentapeptide.biosynthesis.II..lysine.containing.	0.0	-0.106	0.068	-0.240	0.028	0.122	0.395
X1CMET2.PWY..folate.transformations.III..E..coli.	0.0	-0.060	0.061	-0.179	0.059	0.321	0.596
ANAEROFRUCAT.PWY..homolactic.fermentation	0.0	-0.066	0.076	-0.214	0.083	0.388	0.629
ANAGLYCOLYSIS.PWY..glycolysis.III..from.glucose.	0.0	-0.039	0.054	-0.145	0.068	0.474	0.690
ARGININE.SYN4.PWY..L.ornithine.biosynthesis.II	0.0	0.253	0.180	-0.100	0.606	0.160	0.457
ASPASN.PWY..superpathway.of.L.aspartate.and.L.asparagine.biosynthesis	0.0	0.063	0.091	-0.116	0.242	0.489	0.690
BIOTIN.BIOSYNTHESIS.PWY..biotin.biosynthesis.I	0.0	-0.104	0.164	-0.426	0.217	0.526	0.712
BRANCHED.CHAIN.AA.SYN.PWY..superpathway.of.branched.chain.amino.acid.biosynthesis	0.0	-0.110	0.074	-0.256	0.035	0.137	0.424
CITRULBIO.PWY..L.citrulline.biosynthesis	0.0	0.013	0.138	-0.257	0.284	0.924	0.956
COA.PWY.1..superpathway.of.coenzyme.A.biosynthesis.III..mammals.	0.0	-0.072	0.058	-0.186	0.042	0.218	0.486
COA.PWY..coenzyme.A.biosynthesis.I..prokaryotic.	0.0	-0.099	0.092	-0.279	0.081	0.280	0.550
COBALSYN.PWY..superpathway.of.adenosylcobalamin.salvage.from.cobinamide.I	0.0	-0.177	0.130	-0.432	0.077	0.172	0.457
DTDPRHAMSYN.PWY..dTDP..beta..L.rhamnose.biosynthesis	0.0	0.010	0.083	-0.152	0.172	0.904	0.949
FASYN.ELONG.PWY..fatty.acid.elongation....saturated	0.0	-0.115	0.189	-0.485	0.255	0.542	0.724
GLCMANNANAUT.PWY..superpathway.of.N.acetylglucosamine..N.acetylmannosamine.and.N.acetylneu	3.3	-0.286	0.206	-0.689	0.117	0.164	0.457
GLUCONEO.PWY..gluconeogenesis.I	0.0	-0.084	0.094	-0.268	0.100	0.371	0.620
GLUCOSE1PMETAB.PWY..glucose.and.glucose.1.phosphate.degradation	20.0	-0.347	0.272	-0.879	0.185	0.201	0.472
GLYCOLYSIS.E.D..superpathway.of.glycolysis.and.the.Entner.Doudoroff.pathway	0.0	-0.117	0.143	-0.396	0.163	0.413	0.643
GLYCOLYSIS..glycolysis.I..from.glucose.6.phosphate.	0.0	-0.119	0.105	-0.323	0.086	0.257	0.522
HISTSYN.PWY..L.histidine.biosynthesis	0.0	0.061	0.055	-0.046	0.169	0.263	0.522
NONMEVIPP.PWY..methylerythritol.phosphate.pathway.I	0.0	-0.051	0.068	-0.186	0.083	0.452	0.681
PANTO.PWY..phosphopantothenate.biosynthesis.I	0.0	-0.018	0.061	-0.137	0.101	0.766	0.871
PANTOSYN.PWY..superpathway.of.coenzyme.A.biosynthesis.I..bacteria.	0.0	-0.053	0.075	-0.200	0.094	0.478	0.690
PENTOSE.P.PWY..pentose.phosphate.pathway	0.0	-0.058	0.108	-0.269	0.153	0.591	0.759
PEPTIDOGLYCANSYN.PWY..peptidoglycan.biosynthesis.I..meso.diaminopimelate.containing.	0.0	-0.093	0.078	-0.246	0.060	0.233	0.503
PHOSLIPSYN.PWY..superpathway.of.phospholipid.biosynthesis.I..bacteria.	0.0	-0.167	0.159	-0.479	0.146	0.296	0.572
POLYISOPRENSYN.PWY..polyisoprenoid.biosynthesis..E..coli.	10.0	-0.167	0.171	-0.503	0.169	0.330	0.600
PWY.1042..glycolysis.IV	0.0	-0.066	0.090	-0.244	0.111	0.463	0.690
PWY.1269..CMP.3.deoxy.D.manno.octulosonate.biosynthesis	0.0	0.067	0.179	-0.284	0.419	0.707	0.842
PWY.2941..L.lysine.biosynthesis.II	3.3	0.033	0.171	-0.301	0.368	0.846	0.913
PWY.2942..L.lysine.biosynthesis.III	0.0	-0.085	0.074	-0.231	0.060	0.250	0.517
PWY.3841..folate.transformations.II..plants.	0.0	-0.043	0.075	-0.189	0.104	0.566	0.734
PWY.4984..urea.cycle	6.7	0.145	0.248	-0.340	0.631	0.557	0.734
PWY.5103..L.isoleucine.biosynthesis.III	0.0	-0.110	0.081	-0.270	0.050	0.177	0.457
PWY.5121..superpathway.of.geranylgeranyl.diphosphate.biosynthesis.II..via.MEP.	10.0	0.081	0.278	-0.465	0.626	0.772	0.871
PWY.5484..glycolysis.II..from.fructose.6.phosphate.	0.0	-0.101	0.101	-0.300	0.097	0.317	0.596
PWY.5659..GDP.mannose.biosynthesis	0.0	0.070	0.149	-0.221	0.362	0.636	0.804
PWY.5667..CDP.diacylglycerol.biosynthesis.I	0.0	-0.001	0.027	-0.055	0.052	0.956	0.969
PWY.5686..UMP.biosynthesis.I	0.0	-0.069	0.077	-0.220	0.083	0.374	0.620
PWY.5695..inosine.5..phosphate.degradation	0.0	0.036	0.042	-0.047	0.119	0.397	0.637
PWY.5973..cis.vaccenate.biosynthesis	0.0	0.111	0.088	-0.061	0.283	0.204	0.472
PWY.5989..stearate.biosynthesis.II..bacteria.and.plants.	0.0	-0.170	0.185	-0.533	0.193	0.358	0.620
PWY.6123..inosine.5..phosphate.biosynthesis.I	0.0	-0.015	0.074	-0.160	0.130	0.840	0.913
PWY.6124..inosine.5..phosphate.biosynthesis.II	0.0	-0.006	0.074	-0.151	0.140	0.939	0.965
PWY.6125..superpathway.of.guanosine.nucleotides.de.novo.biosynthesis.II	0.0	0.035	0.100	-0.160	0.230	0.725	0.854
PWY.6126..superpathway.of.adenosine.nucleotides.de.novo.biosynthesis.II	0.0	0.024	0.075	-0.123	0.171	0.745	0.857
PWY.6147..6.hydroxymethyl.dihydropterin.diphosphate.biosynthesis.I	0.0	0.200	0.153	-0.100	0.500	0.192	0.469
PWY.6151..S.adenosyl.L.methionine.salvage.I	0.0	-0.117	0.093	-0.299	0.064	0.206	0.472
PWY.6168..flavin.biosynthesis.III..fungi.	23.3	0.042	0.272	-0.491	0.575	0.877	0.933
PWY.621..sucrose.degradation.III..sucrose.invertase.	3.3	-0.163	0.200	-0.556	0.229	0.414	0.643
PWY.6270..isoprene.biosynthesis.I	0.0	-0.101	0.067	-0.232	0.030	0.130	0.413
PWY.6282..palmitoleate.biosynthesis.I..from..5Z..dodec.5.enoate.	0.0	-0.155	0.201	-0.549	0.240	0.443	0.674
PWY.6385..peptidoglycan.biosynthesis.III..mycobacteria.	0.0	-0.085	0.061	-0.206	0.035	0.166	0.457
PWY.6387..UDP.N.acetylmuramoyl.pentapeptide.biosynthesis.I..meso.diaminopimelate.containing.	0.0	-0.107	0.079	-0.261	0.047	0.174	0.457
PWY.6519..8.amino.7.oxononanoate.biosynthesis.I	0.0	-0.118	0.183	-0.478	0.241	0.518	0.711
PWY.6628..superpathway.of.L.phenylalanine.biosynthesis	0.0	-0.053	0.156	-0.358	0.252	0.734	0.854
PWY.6700..queuosine.biosynthesis.I..de.novo.	0.0	-0.063	0.064	-0.188	0.062	0.324	0.596
PWY.6703..preQ0.biosynthesis	0.0	0.116	0.086	-0.053	0.286	0.178	0.457
PWY.6895..superpathway.of.thiamine.diphosphate.biosynthesis.II	23.3	-0.202	0.314	-0.817	0.413	0.520	0.711
PWY.6897..thiamine.diphosphate.salvage.II	0.0	0.042	0.050	-0.057	0.140	0.404	0.640
PWY.6901..superpathway.of.glucose.and.xylose.degradation	0.0	-0.100	0.068	-0.232	0.033	0.139	0.424
PWY.6902..chitin.degradation.II..Vibrio.	0.0	0.307	0.346	-0.371	0.985	0.375	0.620
PWY.7111..pyruvate.fermentation.to.isobutanol..engineered.	0.0	-0.126	0.094	-0.311	0.058	0.180	0.457
PWY.7197..pyrimidine.deoxyribonucleotide.phosphorylation	0.0	-0.028	0.105	-0.235	0.178	0.789	0.874
PWY.7208..superpathway.of.pyrimidine.nucleobases.salvage	0.0	-0.014	0.085	-0.181	0.152	0.866	0.928
PWY.7220..adenosine.deoxyribonucleotides.de.novo.biosynthesis.II	0.0	0.030	0.112	-0.191	0.250	0.792	0.874
PWY.7221..guanosine.ribonucleotides.de.novo.biosynthesis	0.0	-0.059	0.064	-0.184	0.065	0.352	0.620
PWY.7222..guanosine.deoxyribonucleotides.de.novo.biosynthesis.II	0.0	0.030	0.112	-0.191	0.250	0.792	0.874
PWY.7228..superpathway.of.guanosine.nucleotides.de.novo.biosynthesis.I	0.0	0.029	0.085	-0.137	0.195	0.733	0.854
PWY.7229..superpathway.of.adenosine.nucleotides.de.novo.biosynthesis.I	0.0	0.010	0.072	-0.132	0.152	0.893	0.944
PWY.7234..inosine.5..phosphate.biosynthesis.III	6.7	-0.166	0.125	-0.411	0.079	0.183	0.457
PWY.7237..myo...chiro..and.scyllo.inositol.degradation	0.0	0.157	0.119	-0.075	0.390	0.184	0.457
PWY.7282..4.amino.2.methyl.5.diphosphomethylpyrimidine.biosynthesis.II	0.0	-0.037	0.096	-0.225	0.152	0.703	0.842
PWY.7328..superpathway.of.UDP.glucose.derived.O.antigen.building.blocks.biosynthesis	0.0	-0.175	0.141	-0.450	0.101	0.215	0.485
PWY.7345..superpathway.of.anaerobic.sucrose.degradation	0.0	-0.123	0.101	-0.322	0.075	0.223	0.489
PWY.7357..thiamine.phosphate.formation.from.pyrithiamine.and.oxythiamine..yeast.	0.0	-0.058	0.067	-0.189	0.073	0.386	0.629
PWY.7392..taxadiene.biosynthesis..engineered.	13.3	0.132	0.305	-0.466	0.730	0.666	0.820
PWY.7560..methylerythritol.phosphate.pathway.II	0.0	-0.069	0.089	-0.244	0.105	0.437	0.671
PWY.7663..gondoate.biosynthesis..anaerobic.	0.0	0.123	0.110	-0.091	0.338	0.261	0.522
PWY.7664..oleate.biosynthesis.IV..anaerobic.	0.0	-0.130	0.199	-0.521	0.260	0.512	0.711
PWY.7761..NAD.salvage.pathway.II..PNC.IV.cycle.	3.3	-0.095	0.156	-0.401	0.211	0.544	0.724

PWY.7790..UMP.biosynthesis.II	0.0	-0.069	0.077	-0.220	0.083	0.374	0.620
PWY.7791..UMP.biosynthesis.III	0.0	-0.069	0.077	-0.220	0.083	0.374	0.620
PWY.7851..coenzyme.A.biosynthesis.II..eukaryotic.	0.0	-0.060	0.059	-0.177	0.056	0.308	0.588
PWY.7953..UDP.N.acetylmuramoyl.pentapeptide.biosynthesis.III..meso.diaminopimelate.containing.	0.0	-0.093	0.073	-0.236	0.051	0.206	0.472
PWY.7977..L.methionine.biosynthesis.IV	0.0	-0.020	0.051	-0.121	0.080	0.690	0.836
PWY.8187..L.arginine.degradation.XIII..reductive.Stickland.reaction.	0.0	-0.169	0.175	-0.512	0.175	0.336	0.602
PWY.841..superpathway.of.purine.nucleotides.de.novo.biosynthesis.I	0.0	-0.017	0.068	-0.149	0.116	0.806	0.883
PWY0.1319..CDP.diacylglycerol.biosynthesis.II	0.0	-0.001	0.027	-0.055	0.052	0.956	0.969
PWY0.1586..peptidoglycan.maturation..meso.diaminopimelate.containing.	0.0	-0.146	0.126	-0.392	0.100	0.245	0.514
PWY0.162..superpathway.of.pyrimidine.ribonucleotides.de.novo.biosynthesis	0.0	-0.028	0.068	-0.162	0.105	0.680	0.830
PWY0.845..superpathway.of.pyridoxal.5..phosphate.biosynthesis.and.salvage	3.3	0.115	0.166	-0.211	0.440	0.490	0.690
PWY0.862...5Z..dodecenoate.biosynthesis.I	0.0	-0.147	0.211	-0.560	0.266	0.485	0.690
PWY66.409..superpathway.of.purine.nucleotide.salvage	23.3	-0.503	0.353	-1.194	0.189	0.154	0.457
PWY66.429..fatty.acid.biosynthesis.initiation..mitochondria.	0.0	-0.030	0.063	-0.153	0.094	0.636	0.804
PYRIDNUCSYN.PWY..NAD.de.novo.biosynthesis.I..from.aspartate.	0.0	0.024	0.055	-0.084	0.132	0.662	0.820
PYRIDOXSYN.PWY..pyridoxal.5..phosphate.biosynthesis.I	0.0	0.061	0.190	-0.311	0.434	0.747	0.857
RIBOSYN2.PWY..flavin.biosynthesis.I..bacteria.and.plants.	0.0	-0.034	0.077	-0.185	0.116	0.654	0.819
SER.GLYSYN.PWY..superpathway.of.L.serine.and.glycine.biosynthesis.I	0.0	-0.037	0.063	-0.161	0.087	0.562	0.734
THISYNARA.PWY..superpathway.of.thiamine.diphosphate.biosynthesis.III..eukaryotes.	0.0	-0.084	0.122	-0.324	0.156	0.491	0.690
THRESYN.PWY..superpathway.of.L.threonine.biosynthesis	0.0	0.004	0.043	-0.080	0.088	0.921	0.956
TRNA.CHARGING.PWY..tRNA.charging	0.0	-0.070	0.059	-0.185	0.046	0.237	0.504
PWY.6609..adenine.and.adenosine.salvage.III	0.0	0.000	0.050	-0.099	0.099	0.997	0.999
PWY.8004..Entner.Doudoroff.pathway.I	0.0	0.000	0.149	-0.291	0.292	0.999	0.999
