# Supplementary material for: Phage-Display Immunoprecipitation Sequencing Reveals Distinct Antibody Signatures Against Bacterial Flagellins Associated With Treatment Response in Crohn's Disease
Source: Clin Transl Gastroenterol. 2026 Apr 2;17(6):e01030. doi: 10.14309/ctg.0000000000001030 (PMC13641668; doi:10.14309/ctg.0000000000001030)
Supplement: Supplementary file 6 [file ct9-17-e01030-s006.pdf]

Supplementary Table 6

Remission week 22

Pathway	ZP (%)	Coefficient	Std.error	Lower 95% CI	Higher 95% CI	p-Value	q-Value
UDPNAGSYN.PWY..UDP.N.acetyl.D.glucosamine.biosynthesis.I	0.0	-0.535	0.160	-0.848	-0.221	8.33E-04	0.0925
PWY66.409..superpathway.of.purine.nucleotide.salvage	23.1	-0.911	0.308	-1.514	-0.308	0.0031	0.1329
PWY.7117..C4.photosynthetic.carbon.assimilation.cycle..PEPCK.type	11.5	-0.752	0.285	-1.311	-0.193	0.0084	0.1329
PWY.7237..myo...chiro..and.scyllo.inositol.degradation	0.0	0.384	0.143	0.103	0.665	0.0074	0.1329
PWY.7323..superpathway.of.GDP.mannose.derived.O.antigen.building.blocks.biosynthesis	3.8	0.452	0.159	0.140	0.764	0.0045	0.1329
PWY.I9..L.cysteine.biosynthesis.VI..from.L.methionine.	3.8	-0.902	0.330	-1.549	-0.256	0.0062	0.1329
PWY0.1477..ethanolamine.utilization	15.4	-0.788	0.293	-1.361	-0.215	0.0071	0.1329
DTDPRHAMSYN.PWY..dTDP..beta..L.rhamnose.biosynthesis	0.0	0.294	0.120	0.059	0.529	0.0143	0.1985
PWY.5659..GDP.mannose.biosynthesis	0.0	0.301	0.127	0.052	0.550	0.0177	0.2185
PWY.5695..inosine.5..phosphate.degradation	0.0	0.129	0.059	0.013	0.246	0.0291	0.3011
PWY.7328..superpathway.of.UDP.glucose.derived.O.antigen.building.blocks.biosynthesis	0.0	-0.387	0.178	-0.736	-0.038	0.0298	0.3011
PWY.6608..guanosine.nucleotides.degradation.III	0.0	-0.407	0.195	-0.788	-0.025	0.0367	0.3391
PWY.7220..adenosine.deoxyribonucleotides.de.novo.biosynthesis.II	0.0	0.378	0.192	0.003	0.753	0.0485	0.3810
PWY.7222..guanosine.deoxyribonucleotides.de.novo.biosynthesis.II	0.0	0.378	0.192	0.003	0.753	0.0485	0.3810
PWY.5667..CDP.diacylglycerol.biosynthesis.I	0.0	0.155	0.083	-0.009	0.318	0.0637	0.3810
PWY.5973..cis.vaccenate.biosynthesis	0.0	0.213	0.113	-0.007	0.434	0.0582	0.3810
PWY.6125..superpathway.of.guanosine.nucleotides.de.novo.biosynthesis.II	0.0	0.301	0.161	-0.014	0.616	0.0610	0.3810
PWY.7228..superpathway.of.guanosine.nucleotides.de.novo.biosynthesis.I	0.0	0.268	0.146	-0.017	0.554	0.0652	0.3810
PWY0.1319..CDP.diacylglycerol.biosynthesis.II	0.0	0.155	0.083	-0.009	0.318	0.0637	0.3810
DAPLYSINESYN.PWY..L.lysine.biosynthesis.I	11.5	-0.533	0.294	-1.109	0.042	0.0694	0.3834
PWY.7761..NAD.salvage.pathway.II..PNC.IV.cycle.	3.8	-0.532	0.299	-1.117	0.053	0.0749	0.3834
HISDEG.PWY..L.histidine.degradation.I	0.0	0.389	0.219	-0.041	0.818	0.0760	0.3834
ARGININE.SYN4.PWY..L.ornithine.biosynthesis.II	0.0	0.361	0.210	-0.050	0.773	0.0854	0.3867
FERMENTATION.PWY..mixed.acid.fermentation	7.7	-0.467	0.272	-1.000	0.067	0.0863	0.3867
PWY.5188..tetrapyrrole.biosynthesis.I..from.glutamate.	3.8	-0.418	0.245	-0.899	0.062	0.0881	0.3867
OANTIGEN.PWY..O.antigen.building.blocks.biosynthesis..E..coli.	0.0	-0.247	0.148	-0.538	0.044	0.0961	0.3867
PWY.1269..CMP.3.deoxy.D.manno.octulosonate.biosynthesis	0.0	0.308	0.188	-0.060	0.676	0.1010	0.3867
PWY.7663..gondoate.biosynthesis..anaerobic.	0.0	0.172	0.105	-0.033	0.377	0.1001	0.3867
PYRIDNUCSYN.PWY..NAD.de.novo.biosynthesis.I..from.aspartate.	0.0	0.092	0.057	-0.019	0.203	0.1036	0.3867
GLUCONEO.PWY..gluconeogenesis.I	3.8	0.235	0.145	-0.050	0.520	0.1060	0.3867
RIBOSYN2.PWY..flavin.biosynthesis.I..bacteria.and.plants.	3.8	0.250	0.156	-0.055	0.555	0.1080	0.3867
PWY.7229..superpathway.of.adenosine.nucleotides.de.novo.biosynthesis.I	0.0	0.188	0.120	-0.047	0.423	0.1171	0.4060
PWY.6731..starch.degradation.III	0.0	0.381	0.250	-0.109	0.870	0.1273	0.4282
COLANSYN.PWY..colanic.acid.building.blocks.biosynthesis	0.0	0.171	0.114	-0.052	0.393	0.1327	0.4331
P41.PWY..pyruvate.fermentation.to.acetate.and..S..lactate.I	0.0	-0.271	0.188	-0.639	0.097	0.1486	0.4711
PWY.6630..superpathway.of.L.tyrosine.biosynthesis	7.7	-0.434	0.306	-1.033	0.165	0.1555	0.4796
PWY.5030..L.histidine.degradation.III	19.2	0.465	0.339	-0.200	1.130	0.1704	0.5112
PWY.5100..pyruvate.fermentation.to.acetate.and.lactate.II	0.0	-0.248	0.184	-0.608	0.112	0.1775	0.5184
P441.PWY..superpathway.of.N.acetylneuraminate.degradation	11.5	-0.341	0.260	-0.850	0.168	0.1888	0.5241
PWY.7234..inosine.5..phosphate.biosynthesis.III	11.5	-0.387	0.298	-0.972	0.197	0.1939	0.5241
X1CMET2.PWY..folate.transformations.III..E..coli.	0.0	0.114	0.090	-0.062	0.289	0.2050	0.5241
PWY.5971..palmitate.biosynthesis..type.II.fatty.acid.synthase.	57.7	-0.911	0.719	-2.322	0.499	0.2053	0.5241
GLYCOLYSIS.E.D..superpathway.of.glycolysis.and.the.Entner.Doudoroff.pathway	3.8	-0.265	0.212	-0.681	0.151	0.2114	0.5241
GLYCOLYSIS..glycolysis.I..from.glucose.6.phosphate.	0.0	-0.172	0.138	-0.443	0.099	0.2125	0.5241
PWY.6902..chitin.degradation.II..Vibrio.	0.0	-0.330	0.263	-0.845	0.185	0.2095	0.5241
ILEUSYN.PWY..L.isoleucine.biosynthesis.I..from.threonine.	0.0	-0.098	0.080	-0.255	0.059	0.2212	0.5337
NONOXIPENT.PWY..pentose.phosphate.pathway..non.oxidative.branch..I	0.0	-0.133	0.115	-0.359	0.092	0.2465	0.5779
PWY.6628..superpathway.of.L.phenylalanine.biosynthesis	3.8	-0.285	0.248	-0.770	0.200	0.2499	0.5779
PWY.3001..superpathway.of.L.isoleucine.biosynthesis.I	0.0	-0.138	0.124	-0.381	0.105	0.2646	0.5993
PWY.4984..urea.cycle	11.5	0.285	0.260	-0.224	0.794	0.2729	0.6011
PWY.6147..6.hydroxymethyl.dihydropterin.diphosphate.biosynthesis.I	0.0	0.151	0.139	-0.121	0.423	0.2762	0.6011
PWY.7208..superpathway.of.pyrimidine.nucleobases.salvage	0.0	0.134	0.128	-0.116	0.384	0.2929	0.6253
ANAEROFRUCAT.PWY..homolactic.fermentation	0.0	-0.123	0.127	-0.372	0.126	0.3329	0.6387
GLCMANNANAUT.PWY..superpathway.of.N.acetylglucosamine..N.acetylmannosamine.and.N.acetylneu	0.0	-0.155	0.176	-0.500	0.191	0.3809	0.6505
GLUCUROCAT.PWY..superpathway.of..beta..D.glucuronosides.degradation	0.0	0.213	0.226	-0.230	0.656	0.3463	0.6387
GLYCOGENSYNTH.PWY..glycogen.biosynthesis.I..from.ADP.D.Glucose.	0.0	0.132	0.130	-0.123	0.388	0.3098	0.6253
NAGLIPASYN.PWY..lipid.IVA.biosynthesis..E..coli.	0.0	0.209	0.217	-0.216	0.634	0.3344	0.6387
PANTOSYN.PWY..superpathway.of.coenzyme.A.biosynthesis.I..bacteria.	0.0	0.099	0.119	-0.134	0.332	0.4039	0.6576
PWY.1042..glycolysis.IV	0.0	0.093	0.090	-0.084	0.270	0.3031	0.6253
PWY.2941..L.lysine.biosynthesis.II	0.0	0.165	0.183	-0.193	0.523	0.3663	0.6425
PWY.2942..L.lysine.biosynthesis.III	0.0	-0.077	0.083	-0.240	0.085	0.3510	0.6387
PWY.3841..folate.transformations.II..plants.	0.0	0.063	0.070	-0.075	0.200	0.3705	0.6425
PWY.5484..glycolysis.II..from.fructose.6.phosphate.	0.0	-0.101	0.122	-0.341	0.138	0.4068	0.6576
PWY.621..sucrose.degradation.III..sucrose.invertase.	0.0	0.245	0.240	-0.224	0.715	0.3061	0.6253
PWY.6703..preQ0.biosynthesis	0.0	0.158	0.173	-0.182	0.498	0.3625	0.6425
PWY.8073..lipid.IVA.biosynthesis..P..putida.	0.0	0.209	0.217	-0.216	0.634	0.3344	0.6387
PWY.8187..L.arginine.degradation.XIII..reductive.Stickland.reaction.	3.8	-0.191	0.232	-0.645	0.263	0.4088	0.6576
PWY0.162..superpathway.of.pyrimidine.ribonucleotides.de.novo.biosynthesis	3.8	0.136	0.162	-0.182	0.453	0.4016	0.6576
THISYNARA.PWY..superpathway.of.thiamine.diphosphate.biosynthesis.III..eukaryotes.	0.0	-0.112	0.120	-0.347	0.123	0.3494	0.6387
PYRIDOXSYN.PWY..pyridoxal.5..phosphate.biosynthesis.I	0.0	0.191	0.241	-0.281	0.663	0.4280	0.6678
PWY4FS.7..phosphatidylglycerol.biosynthesis.I..plastidic.	0.0	-0.148	0.189	-0.518	0.223	0.4343	0.6678
PWY4FS.8..phosphatidylglycerol.biosynthesis.II..non.plastidic.	0.0	-0.148	0.189	-0.518	0.223	0.4343	0.6678
PWY0.1586..peptidoglycan.maturation..meso.diaminopimelate.containing.	0.0	0.083	0.107	-0.127	0.293	0.4392	0.6678
PWY.6353..purine.nucleotides.degradation.II..aerobic.	7.7	-0.175	0.235	-0.636	0.285	0.4548	0.6822
PWY.5121..superpathway.of.geranylgeranyl.diphosphate.biosynthesis.II..via.MEP.	3.8	0.212	0.288	-0.352	0.775	0.4613	0.6827
PANTO.PWY..phosphopantothenate.biosynthesis.I	0.0	0.079	0.109	-0.136	0.293	0.4720	0.6838
PWY.7345..superpathway.of.anaerobic.sucrose.degradation	3.8	-0.158	0.221	-0.591	0.275	0.4743	0.6838
PWY.8178..pentose.phosphate.pathway..non.oxidative.branch..II	0.0	-0.081	0.116	-0.309	0.147	0.4861	0.6918
CITRULBIO.PWY..L.citrulline.biosynthesis	3.8	0.121	0.188	-0.247	0.489	0.5195	0.7299
PWY.6123..inosine.5..phosphate.biosynthesis.I	0.0	0.056	0.089	-0.119	0.232	0.5281	0.7328

GLUCOSE1PMETAB.PWY..glucose.and.glucose.1.phosphate.degradation	34.6	-0.298	0.510	-1.299	0.702	0.5588	0.7658
BIOTIN.BIOSYNTHESIS.PWY..biotin.biosynthesis.I	3.8	0.094	0.194	-0.287	0.476	0.6274	0.8390
PWY.6527..stachyose.degradation	3.8	-0.120	0.248	-0.606	0.365	0.6271	0.8390
PWY.7242..D.fructuronate.degradation	0.0	0.103	0.225	-0.337	0.543	0.6465	0.8473
PWY.6519..8.amino.7.oxononanoate.biosynthesis.I	3.8	0.091	0.200	-0.301	0.484	0.6488	0.8473
PWY0.862...5Z..dodecenoate.biosynthesis.I	3.8	0.105	0.242	-0.369	0.579	0.6637	0.8566
COA.PWY..coenzyme.A.biosynthesis.I..prokaryotic.	0.0	-0.033	0.078	-0.186	0.120	0.6741	0.8601
PWY.6270..isoprene.biosynthesis.I	3.8	0.061	0.155	-0.243	0.366	0.6923	0.8732
PEPTIDOGLYCANSYN.PWY..peptidoglycan.biosynthesis.I..meso.diaminopimelate.containing.	0.0	-0.023	0.070	-0.160	0.114	0.7413	0.9241
PWY.6387..UDP.N.acetylmuramoyl.pentapeptide.biosynthesis.I..meso.diaminopimelate.containing.	0.0	-0.023	0.071	-0.161	0.116	0.7493	0.9241
PENTOSE.P.PWY..pentose.phosphate.pathway	0.0	0.029	0.117	-0.200	0.259	0.8011	0.9665
PWY.6168..flavin.biosynthesis.III..fungi.	15.4	0.069	0.271	-0.463	0.600	0.8000	0.9665
PWY.6901..superpathway.of.glucose.and.xylose.degradation	3.8	0.038	0.166	-0.287	0.364	0.8181	0.9728
PWY.6305..superpathway.of.putrescine.biosynthesis	3.8	-0.052	0.233	-0.508	0.404	0.8238	0.9728
PWY.5384..sucrose.degradation.IV..sucrose.phosphorylase.	0.0	-0.040	0.224	-0.480	0.399	0.8572	0.9857
PWY.6895..superpathway.of.thiamine.diphosphate.biosynthesis.II	19.2	-0.053	0.282	-0.604	0.499	0.8518	0.9857
PWY.7221..guanosine.ribonucleotides.de.novo.biosynthesis	0.0	0.012	0.070	-0.125	0.149	0.8631	0.9857
PWY.8004..Entner.Doudoroff.pathway.I	3.8	0.029	0.200	-0.363	0.421	0.8844	0.9857
COBALSYN.PWY..superpathway.of.adenosylcobalamin.salvage.from.cobinamide.I	0.0	0.027	0.247	-0.456	0.511	0.9113	0.9857
PWY.6282..palmitoleate.biosynthesis.I..from..5Z..dodec.5.enoate.	3.8	-0.024	0.239	-0.493	0.445	0.9205	0.9857
PWY.7197..pyrimidine.deoxyribonucleotide.phosphorylation	0.0	-0.017	0.175	-0.361	0.326	0.9217	0.9857
PWY0.1296..purine.ribonucleosides.degradation	3.8	-0.017	0.190	-0.390	0.355	0.9270	0.9857
GLYCOCAT.PWY..glycogen.degradation.I	3.8	-0.022	0.336	-0.680	0.636	0.9475	0.9857
POLYISOPRENSYN.PWY..polyisoprenoid.biosynthesis..E..coli.	3.8	0.016	0.237	-0.449	0.481	0.9453	0.9857
PWY.7664..oleate.biosynthesis.IV..anaerobic.	3.8	0.014	0.228	-0.433	0.460	0.9526	0.9857
PWY.5989..stearate.biosynthesis.II..bacteria.and.plants.	3.8	-0.008	0.250	-0.499	0.482	0.9731	0.9857
FASYN.ELONG.PWY..fatty.acid.elongation....saturated	3.8	0.005	0.220	-0.426	0.436	0.9828	0.9857
PWY.5941..glycogen.degradation.II	0.0	0.003	0.152	-0.296	0.301	0.9857	0.9857
PWY.7392..taxadiene.biosynthesis..engineered.	19.2	-0.011	0.381	-0.758	0.737	0.9780	0.9857
PWY.7953..UDP.N.acetylmuramoyl.pentapeptide.biosynthesis.III..meso.diaminopimelate.containing.	0.0	-0.002	0.075	-0.148	0.144	0.9819	0.9857

Non-remission week 22

Pathway	ZP (%)	Coefficient	Std.error	Lower 95% CI	Higher 95% CI	p-Value	q-Value
PWY.6936..seleno.amino.acid.biosynthesis..plants.	0.0	-0.556	0.173	-0.895	-0.217	0.0013	0.1121
PWY.7237..myo...chiro..and.scyllo.inositol.degradation	0.0	0.305	0.102	0.105	0.505	0.0028	0.1121
UDPNAGSYN.PWY..UDP.N.acetyl.D.glucosamine.biosynthesis.I	0.0	-0.428	0.143	-0.709	-0.147	0.0028	0.1121
PWY0.1296..purine.ribonucleosides.degradation	0.0	-0.317	0.108	-0.529	-0.106	0.0033	0.1121
PWY.6630..superpathway.of.L.tyrosine.biosynthesis	0.0	-0.367	0.129	-0.620	-0.113	0.0045	0.1243
PWY66.409..superpathway.of.purine.nucleotide.salvage	20.8	-1.017	0.369	-1.741	-0.294	0.0059	0.1339
PWY.6731..starch.degradation.III	0.0	-0.251	0.096	-0.438	-0.063	0.0089	0.1687
PWY.6823..molybdopterin.biosynthesis	0.0	-0.425	0.165	-0.748	-0.102	0.0099	0.1687
ARO.PWY..chorismate.biosynthesis.I	0.0	-0.128	0.052	-0.229	-0.026	0.0138	0.1724
PWY.2942..L.lysine.biosynthesis.III	0.0	-0.129	0.052	-0.232	-0.027	0.0137	0.1724
PWY.5100..pyruvate.fermentation.to.acetate.and.lactate.II	0.0	-0.473	0.189	-0.844	-0.103	0.0123	0.1724
PWY0.1297..superpathway.of.purine.deoxyribonucleosides.degradation	16.7	-0.983	0.406	-1.779	-0.188	0.0154	0.1761
P41.PWY..pyruvate.fermentation.to.acetate.and..S..lactate.I	0.0	-0.461	0.195	-0.844	-0.078	0.0183	0.1927
COMPLETE.ARO.PWY..superpathway.of.aromatic.amino.acid.biosynthesis	0.0	-0.122	0.052	-0.224	-0.019	0.0199	0.1946
PWY.6897..thiamine.diphosphate.salvage.II	0.0	0.162	0.071	0.023	0.301	0.0221	0.2016
PWY.1269..CMP.3.deoxy.D.manno.octulosonate.biosynthesis	4.2	0.464	0.211	0.050	0.879	0.0279	0.2362
THISYNARA.PWY..superpathway.of.thiamine.diphosphate.biosynthesis.III..eukaryotes.	0.0	0.185	0.085	0.019	0.352	0.0293	0.2362
PWY.7234..inosine.5..phosphate.biosynthesis.III	4.2	-0.330	0.156	-0.636	-0.025	0.0340	0.2362
ARGSYNBSUB.PWY..L.arginine.biosynthesis.II..acetyl.cycle.	0.0	-0.316	0.151	-0.612	-0.020	0.0362	0.2362
PWY.6147..6.hydroxymethyl.dihydropterin.diphosphate.biosynthesis.I	0.0	0.328	0.156	0.023	0.633	0.0352	0.2362
PWY.7238..sucrose.biosynthesis.II	0.0	-0.193	0.092	-0.373	-0.012	0.0362	0.2362
GLUCONEO.PWY..gluconeogenesis.I	0.0	-0.143	0.069	-0.279	-0.007	0.0396	0.2381
RHAMCAT.PWY..L.rhamnose.degradation.I	0.0	0.324	0.158	0.014	0.634	0.0405	0.2381
PWY.7111..pyruvate.fermentation.to.isobutanol..engineered.	0.0	-0.264	0.132	-0.523	-0.005	0.0457	0.2381
PWY.7761..NAD.salvage.pathway.II..PNC.IV.cycle.	0.0	-0.287	0.143	-0.567	-0.006	0.0452	0.2381
CALVIN.PWY..Calvin.Benson.Bassham.cycle	0.0	-0.211	0.107	-0.420	-0.002	0.0478	0.2381
PWY.5695..inosine.5..phosphate.degradation	0.0	0.075	0.038	0.001	0.150	0.0484	0.2381
PWY.7953..UDP.N.acetylmuramoyl.pentapeptide.biosynthesis.III..meso.diaminopimelate.containing.	0.0	-0.104	0.053	-0.207	0.000	0.0490	0.2381
PWY.6386..UDP.N.acetylmuramoyl.pentapeptide.biosynthesis.II..lysine.containing.	0.0	-0.111	0.058	-0.225	0.003	0.0573	0.2514
HSERMETANA.PWY..L.methionine.biosynthesis.III	0.0	-0.266	0.141	-0.542	0.010	0.0588	0.2514
PEPTIDOGLYCANSYN.PWY..peptidoglycan.biosynthesis.I..meso.diaminopimelate.containing.	0.0	-0.101	0.054	-0.206	0.005	0.0610	0.2514
PWY.5941..glycogen.degradation.II	0.0	-0.237	0.128	-0.488	0.014	0.0643	0.2514
PWY.6387..UDP.N.acetylmuramoyl.pentapeptide.biosynthesis.I..meso.diaminopimelate.containing.	0.0	-0.101	0.055	-0.208	0.006	0.0635	0.2514
PWY.8187..L.arginine.degradation.XIII..reductive.Stickland.reaction.	0.0	-0.399	0.217	-0.824	0.025	0.0654	0.2514
PWY0.845..superpathway.of.pyridoxal.5..phosphate.biosynthesis.and.salvage	4.2	0.354	0.192	-0.023	0.731	0.0660	0.2514
ANAEROFRUCAT.PWY..homolactic.fermentation	0.0	-0.198	0.109	-0.411	0.016	0.0699	0.2589
FERMENTATION.PWY..mixed.acid.fermentation	0.0	-0.335	0.186	-0.701	0.030	0.0720	0.2596
PWY.6385..peptidoglycan.biosynthesis.III..mycobacteria.	0.0	-0.089	0.050	-0.187	0.009	0.0739	0.2596
ARGININE.SYN4.PWY..L.ornithine.biosynthesis.II	0.0	0.425	0.244	-0.053	0.902	0.0811	0.2694
COA.PWY..coenzyme.A.biosynthesis.I..prokaryotic.	0.0	-0.097	0.057	-0.208	0.015	0.0887	0.2694
GLYCOGENSYNTH.PWY..glycogen.biosynthesis.I..from.ADP.D.Glucose.	0.0	-0.232	0.138	-0.503	0.038	0.0923	0.2694
PWY.5097..L.lysine.biosynthesis.VI	0.0	-0.083	0.049	-0.180	0.013	0.0915	0.2694
PWY.6121..5.aminoimidazole.ribonucleotide.biosynthesis.I	0.0	-0.077	0.046	-0.166	0.012	0.0915	0.2694
PWY.6628..superpathway.of.L.phenylalanine.biosynthesis	0.0	-0.168	0.100	-0.364	0.028	0.0932	0.2694
PWY.7400..L.arginine.biosynthesis.IV..archaebacteria.	8.3	-0.376	0.220	-0.808	0.055	0.0874	0.2694
DTDPRHAMSYN.PWY..dTDP..beta..L.rhamnose.biosynthesis	0.0	0.139	0.086	-0.030	0.307	0.1065	0.2694
NAGLIPASYN.PWY..lipid.IVA.biosynthesis..E..coli.	4.2	0.537	0.336	-0.122	1.196	0.1100	0.2694
PWY.3001..superpathway.of.L.isoleucine.biosynthesis.I	0.0	-0.108	0.066	-0.238	0.022	0.1049	0.2694
PWY.6151..S.adenosyl.L.methionine.salvage.I	0.0	-0.145	0.090	-0.322	0.032	0.1092	0.2694
PWY.6163..chorismate.biosynthesis.from.3.dehydroquinate	0.0	-0.084	0.052	-0.186	0.019	0.1101	0.2694
PWY.6353..purine.nucleotides.degradation.II..aerobic.	12.5	-0.432	0.261	-0.944	0.079	0.0975	0.2694

PWY.6609..adenine.and.adenosine.salvage.III	0.0	-0.095	0.058	-0.209	0.018	0.1004	0.2694
PWY.724..superpathway.of.L.lysine..L.threonine.and.L.methionine.biosynthesis.II	0.0	-0.068	0.042	-0.150	0.014	0.1036	0.2694
PWY.7851..coenzyme.A.biosynthesis.II..eukaryotic.	0.0	-0.092	0.056	-0.201	0.018	0.1017	0.2694
PWY.8073..lipid.IVA.biosynthesis..P..putida.	4.2	0.537	0.336	-0.122	1.196	0.1100	0.2694
PWY.6122..5.aminoimidazole.ribonucleotide.biosynthesis.II	0.0	-0.083	0.054	-0.188	0.023	0.1235	0.2918
PWY.6277..superpathway.of.5.aminoimidazole.ribonucleotide.biosynthesis	0.0	-0.083	0.054	-0.188	0.023	0.1235	0.2918
COA.PWY.1..superpathway.of.coenzyme.A.biosynthesis.III..mammals.	0.0	-0.089	0.059	-0.205	0.026	0.1308	0.3036
PWY.8178..pentose.phosphate.pathway..non.oxidative.branch..II	0.0	-0.208	0.139	-0.480	0.064	0.1344	0.3069
NONOXIPENT.PWY..pentose.phosphate.pathway..non.oxidative.branch..I	0.0	-0.225	0.154	-0.527	0.076	0.1432	0.3216
PYRIDOXSYN.PWY..pyridoxal.5..phosphate.biosynthesis.I	4.2	0.362	0.253	-0.135	0.859	0.1530	0.3380
GLYCOLYSIS..glycolysis.I..from.glucose.6.phosphate.	0.0	-0.159	0.114	-0.383	0.066	0.1653	0.3493
PWY.7790..UMP.biosynthesis.II	0.0	-0.070	0.050	-0.169	0.029	0.1657	0.3493
PWY.7791..UMP.biosynthesis.III	0.0	-0.070	0.050	-0.169	0.029	0.1657	0.3493
PWY.5686..UMP.biosynthesis.I	0.0	-0.068	0.051	-0.168	0.031	0.1798	0.3677
PWY.6317..D.galactose.degradation.I..Leloir.pathway.	0.0	-0.191	0.142	-0.470	0.088	0.1798	0.3677
HISDEG.PWY..L.histidine.degradation.I	0.0	0.372	0.284	-0.183	0.928	0.1890	0.3809
PWY.7117..C4.photosynthetic.carbon.assimilation.cycle..PEPCK.type	8.3	-0.286	0.220	-0.717	0.145	0.1930	0.3832
X1CMET2.PWY..folate.transformations.III..E..coli.	0.0	-0.057	0.044	-0.144	0.030	0.2019	0.3952
PWY.5973..cis.vaccenate.biosynthesis	0.0	0.106	0.085	-0.060	0.272	0.2124	0.4099
PYRIDNUCSYN.PWY..NAD.de.novo.biosynthesis.I..from.aspartate.	0.0	0.072	0.060	-0.044	0.189	0.2240	0.4261
PHOSLIPSYN.PWY..superpathway.of.phospholipid.biosynthesis.I..bacteria.	0.0	0.155	0.130	-0.100	0.410	0.2343	0.4352
PWY.7663..gondoate.biosynthesis..anaerobic.	0.0	0.153	0.129	-0.100	0.406	0.2359	0.4352
PANTOSYN.PWY..superpathway.of.coenzyme.A.biosynthesis.I..bacteria.	0.0	-0.070	0.060	-0.188	0.047	0.2414	0.4352
PWY0.1586..peptidoglycan.maturation..meso.diaminopimelate.containing.	0.0	0.140	0.119	-0.094	0.374	0.2405	0.4352
PWY.I9..L.cysteine.biosynthesis.VI..from.L.methionine.	4.2	-0.232	0.200	-0.625	0.160	0.2455	0.4367
PWY.5030..L.histidine.degradation.III	0.0	0.346	0.304	-0.251	0.943	0.2557	0.4434
PWY.6703..preQ0.biosynthesis	0.0	0.144	0.127	-0.104	0.392	0.2556	0.4434
TRNA.CHARGING.PWY..tRNA.charging	0.0	-0.055	0.049	-0.152	0.042	0.2639	0.4520
PWY.5484..glycolysis.II..from.fructose.6.phosphate.	0.0	-0.129	0.122	-0.368	0.110	0.2895	0.4897
GLCMANNANAUT.PWY..superpathway.of.N.acetylglucosamine..N.acetylmannosamine.and.N.acetylneu	4.2	-0.239	0.229	-0.688	0.209	0.2961	0.4947
DAPLYSINESYN.PWY..L.lysine.biosynthesis.I	0.0	-0.260	0.262	-0.774	0.254	0.3217	0.5248
PWY.7977..L.methionine.biosynthesis.IV	0.0	0.070	0.069	-0.065	0.206	0.3084	0.5090
ANAGLYCOLYSIS.PWY..glycolysis.III..from.glucose.	0.0	-0.062	0.064	-0.188	0.064	0.3354	0.5388
PWY.6168..flavin.biosynthesis.III..fungi.	33.3	0.421	0.440	-0.441	1.283	0.3382	0.5388
PWY.6895..superpathway.of.thiamine.diphosphate.biosynthesis.II	29.2	-0.387	0.413	-1.197	0.423	0.3494	0.5493
BRANCHED.CHAIN.AA.SYN.PWY..superpathway.of.branched.chain.amino.acid.biosynthesis	0.0	-0.064	0.068	-0.198	0.071	0.3528	0.5493
PWY.6700..queuosine.biosynthesis.I..de.novo.	0.0	-0.037	0.040	-0.115	0.042	0.3611	0.5551
PWY.3841..folate.transformations.II..plants.	0.0	-0.043	0.049	-0.139	0.053	0.3768	0.5551
PWY.5667..CDP.diacylglycerol.biosynthesis.I	0.0	-0.039	0.044	-0.124	0.047	0.3759	0.5551
PWY.7197..pyrimidine.deoxyribonucleotide.phosphorylation	0.0	-0.110	0.124	-0.352	0.133	0.3763	0.5551
PWY0.1319..CDP.diacylglycerol.biosynthesis.II	0.0	-0.039	0.044	-0.124	0.047	0.3759	0.5551
BIOTIN.BIOSYNTHESIS.PWY..biotin.biosynthesis.I	8.3	0.222	0.261	-0.290	0.734	0.3947	0.5753
PWY.6608..guanosine.nucleotides.degradation.III	0.0	-0.177	0.213	-0.595	0.241	0.4063	0.5860
PWY0.162..superpathway.of.pyrimidine.ribonucleotides.de.novo.biosynthesis	0.0	0.077	0.095	-0.110	0.263	0.4213	0.6013
ASPASN.PWY..superpathway.of.L.aspartate.and.L.asparagine.biosynthesis	0.0	0.067	0.086	-0.101	0.235	0.4323	0.6039
PANTO.PWY..phosphopantothenate.biosynthesis.I	0.0	-0.051	0.065	-0.178	0.077	0.4364	0.6039
PWY.6519..8.amino.7.oxononanoate.biosynthesis.I	8.3	0.231	0.295	-0.347	0.809	0.4332	0.6039
POLYISOPRENSYN.PWY..polyisoprenoid.biosynthesis..E..coli.	4.2	-0.117	0.156	-0.423	0.189	0.4553	0.6185
PWY.7357..thiamine.phosphate.formation.from.pyrithiamine.and.oxythiamine..yeast.	0.0	0.065	0.087	-0.106	0.236	0.4559	0.6185
VALSYN.PWY..L.valine.biosynthesis	0.0	-0.041	0.056	-0.150	0.069	0.4687	0.6296
PWY.6901..superpathway.of.glucose.and.xylose.degradation	0.0	-0.112	0.163	-0.432	0.208	0.4925	0.6550
GLUCOSE1PMETAB.PWY..glucose.and.glucose.1.phosphate.degradation	29.2	-0.271	0.408	-1.070	0.528	0.5066	0.6674
FASYN.ELONG.PWY..fatty.acid.elongation....saturated	8.3	0.141	0.217	-0.284	0.566	0.5149	0.6718
PWY.7199..pyrimidine.deoxyribonucleosides.salvage	0.0	0.028	0.046	-0.061	0.118	0.5342	0.6904
SER.GLYSYN.PWY..superpathway.of.L.serine.and.glycine.biosynthesis.I	0.0	-0.060	0.101	-0.258	0.138	0.5523	0.7072
CITRULBIO.PWY..L.citrulline.biosynthesis	4.2	0.138	0.242	-0.338	0.613	0.5702	0.7233
PWY.7664..oleate.biosynthesis.IV..anaerobic.	8.3	0.160	0.302	-0.431	0.751	0.5963	0.7380
PWY0.862...5Z..dodecenoate.biosynthesis.I	8.3	0.166	0.314	-0.449	0.781	0.5973	0.7380
PWY.5989..stearate.biosynthesis.II..bacteria.and.plants.	4.2	0.111	0.218	-0.315	0.538	0.6087	0.7380
PWY.6282..palmitoleate.biosynthesis.I..from..5Z..dodec.5.enoate.	8.3	0.159	0.307	-0.442	0.760	0.6046	0.7380
PWY.7221..guanosine.ribonucleotides.de.novo.biosynthesis	0.0	-0.027	0.052	-0.128	0.075	0.6071	0.7380
PWY.2941..L.lysine.biosynthesis.II	0.0	-0.077	0.188	-0.445	0.291	0.6807	0.8180
PWY.6123..inosine.5..phosphate.biosynthesis.I	0.0	-0.020	0.054	-0.126	0.085	0.7043	0.8390
PWY.7345..superpathway.of.anaerobic.sucrose.degradation	0.0	0.042	0.128	-0.209	0.294	0.7406	0.8746
PWY0.1261..anhydromuropeptides.recycling.I	4.2	0.070	0.236	-0.393	0.533	0.7675	0.8987
PWY4FS.7..phosphatidylglycerol.biosynthesis.I..plastidic.	0.0	0.042	0.162	-0.275	0.360	0.7938	0.9117
PWY4FS.8..phosphatidylglycerol.biosynthesis.II..non.plastidic.	0.0	0.042	0.162	-0.275	0.360	0.7938	0.9117
NONMEVIPP.PWY..methylerythritol.phosphate.pathway.I	0.0	-0.014	0.061	-0.134	0.105	0.8155	0.9117
PWY66.429..fatty.acid.biosynthesis.initiation..mitochondria.	0.0	-0.009	0.038	-0.084	0.066	0.8147	0.9117
PWY.5154..L.arginine.biosynthesis.III..via.N.acetyl.L.citrulline.	0.0	0.023	0.100	-0.173	0.219	0.8186	0.9117
PWY.5384..sucrose.degradation.IV..sucrose.phosphorylase.	0.0	-0.044	0.196	-0.429	0.341	0.8219	0.9117
PWY.5659..GDP.mannose.biosynthesis	0.0	0.030	0.155	-0.274	0.334	0.8451	0.9117
PWY.6124..inosine.5..phosphate.biosynthesis.II	0.0	-0.012	0.061	-0.133	0.108	0.8392	0.9117
PWY.841..superpathway.of.purine.nucleotides.de.novo.biosynthesis.I	0.0	-0.014	0.072	-0.155	0.127	0.8425	0.9117
RIBOSYN2.PWY..flavin.biosynthesis.I..bacteria.and.plants.	0.0	-0.019	0.088	-0.191	0.153	0.8270	0.9117
COBALSYN.PWY..superpathway.of.adenosylcobalamin.salvage.from.cobinamide.I	0.0	0.025	0.136	-0.243	0.292	0.8568	0.9131
GLUCUROCAT.PWY..superpathway.of..beta..D.glucuronosides.degradation	4.2	0.032	0.199	-0.358	0.422	0.8722	0.9131
PWY.7220..adenosine.deoxyribonucleotides.de.novo.biosynthesis.II	0.0	0.024	0.151	-0.272	0.320	0.8731	0.9131
PWY.7222..guanosine.deoxyribonucleotides.de.novo.biosynthesis.II	0.0	0.024	0.151	-0.272	0.320	0.8731	0.9131
COLANSYN.PWY..colanic.acid.building.blocks.biosynthesis	0.0	0.015	0.125	-0.230	0.261	0.9016	0.9238
HISTSYN.PWY..L.histidine.biosynthesis	0.0	0.007	0.057	-0.106	0.120	0.9033	0.9238
PWY.7392..taxadiene.biosynthesis..engineered.	4.2	0.025	0.205	-0.376	0.426	0.9036	0.9238
THRESYN.PWY..superpathway.of.L.threonine.biosynthesis	0.0	-0.005	0.057	-0.117	0.107	0.9295	0.9432
PENTOSE.P.PWY..pentose.phosphate.pathway	0.0	0.003	0.147	-0.285	0.291	0.9848	0.9920

PWY.7323..superpathway.of.GDP.mannose.derived.O.antigen.building.blocks.biosynthesis	4.2	0.000	0.171	-0.335	0.334	0.9981	0.9981
