# Supplementary material for: Phage-Display Immunoprecipitation Sequencing Reveals Distinct Antibody Signatures Against Bacterial Flagellins Associated With Treatment Response in Crohn's Disease
Source: Clin Transl Gastroenterol. 2026 Apr 2;17(6):e01030. doi: 10.14309/ctg.0000000000001030 (PMC13641668; doi:10.14309/ctg.0000000000001030)
Supplement: Supplementary file 7 [file ct9-17-e01030-s007.pdf]

Supplementary Table 7

Remission week 52

Pathway	ZP (%)	Coefficient	Std.error	Lower 95% CI	Higher 95% CI	p-Value	q-Value
PWY.6387..UDP.N.acetylmuramoyl.pentapeptide.biosynthesis.I..meso.diaminopimelate.containing.	0.0	-0.126	0.033	-0.191	-0.061	1.40E-04	0.0156
PWY.6353..purine.nucleotides.degradation.II..aerobic.	0.0	-0.559	0.166	-0.884	-0.235	7.38E-04	0.0413
PWY.6936..seleno.amino.acid.biosynthesis..plants.	0.0	-0.381	0.119	-0.614	-0.148	0.0013	0.0502
P441.PWY..superpathway.of.N.acetylneuraminate.degradation	12.5	-0.618	0.224	-1.057	-0.179	0.0058	0.1384
PWY.I9..L.cysteine.biosynthesis.VI..from.L.methionine.	0.0	-0.777	0.284	-1.333	-0.221	0.0062	0.1384
PWY.5345..superpathway.of.L.methionine.biosynthesis..by.sulfhydrylation.	18.8	-1.022	0.397	-1.800	-0.244	0.0100	0.1834
PWY.6163..chorismate.biosynthesis.from.3.dehydroquinate	0.0	-0.082	0.032	-0.145	-0.018	0.0117	0.1834
CITRULBIO.PWY..L.citrulline.biosynthesis	6.3	0.528	0.219	0.099	0.957	0.0159	0.1901
PWY.4984..urea.cycle	12.5	0.941	0.379	0.198	1.684	0.0131	0.1834
PWY.7237..myo...chiro..and.scyllo.inositol.degradation	0.0	0.368	0.154	0.066	0.670	0.0170	0.1901
COMPLETE.ARO.PWY..superpathway.of.aromatic.amino.acid.biosynthesis	0.0	-0.091	0.040	-0.169	-0.014	0.0208	0.2119
PWY.7117..C4.photosynthetic.carbon.assimilation.cycle..PEPCK.type	6.3	-0.605	0.272	-1.138	-0.072	0.0260	0.2231
VALSYN.PWY..L.valine.biosynthesis	0.0	-0.129	0.058	-0.244	-0.015	0.0267	0.2231
ARG.POLYAMINE.SYN..superpathway.of.arginine.and.polyamine.biosynthesis	12.5	-0.772	0.355	-1.468	-0.076	0.0297	0.2231
PWY.724..superpathway.of.L.lysine..L.threonine.and.L.methionine.biosynthesis.II	0.0	-0.113	0.052	-0.215	-0.011	0.0299	0.2231
PWY.7323..superpathway.of.GDP.mannose.derived.O.antigen.building.blocks.biosynthesis	6.3	0.463	0.220	0.032	0.893	0.0352	0.2461
PWY66.409..superpathway.of.purine.nucleotide.salvage	6.3	-0.530	0.255	-1.030	-0.031	0.0375	0.2473
ARO.PWY..chorismate.biosynthesis.I	0.0	-0.076	0.039	-0.154	0.001	0.0529	0.3289
PWY.6608..guanosine.nucleotides.degradation.III	0.0	-0.493	0.260	-1.003	0.016	0.0578	0.3296
PWY.7761..NAD.salvage.pathway.II..PNC.IV.cycle.	0.0	-0.680	0.368	-1.402	0.041	0.0646	0.3296
NAGLIPASYN.PWY..lipid.IVA.biosynthesis..E..coli.	6.3	-0.504	0.276	-1.044	0.037	0.0677	0.3296
PWY.8073..lipid.IVA.biosynthesis..P..putida.	6.3	-0.504	0.276	-1.044	0.037	0.0677	0.3296
PWY0.1296..purine.ribonucleosides.degradation	0.0	-0.170	0.094	-0.355	0.015	0.0721	0.3366
PWY.6823..molybdopterin.biosynthesis	0.0	-0.283	0.164	-0.605	0.039	0.0851	0.3802
PWY.6630..superpathway.of.L.tyrosine.biosynthesis	6.3	-0.512	0.300	-1.100	0.077	0.0883	0.3802
ILEUSYN.PWY..L.isoleucine.biosynthesis.I..from.threonine.	0.0	-0.103	0.062	-0.226	0.019	0.0992	0.3913
HSERMETANA.PWY..L.methionine.biosynthesis.III	0.0	-0.174	0.106	-0.383	0.035	0.1019	0.3913
GLUTORN.PWY..L.ornithine.biosynthesis.I	0.0	-0.159	0.098	-0.352	0.033	0.1052	0.3913
PWY.6125..superpathway.of.guanosine.nucleotides.de.novo.biosynthesis.II	0.0	0.295	0.189	-0.076	0.665	0.1188	0.3913
PWY.7111..pyruvate.fermentation.to.isobutanol..engineered.	0.0	-0.256	0.163	-0.577	0.064	0.1170	0.3913
PWY.7220..adenosine.deoxyribonucleotides.de.novo.biosynthesis.II	0.0	0.347	0.220	-0.085	0.779	0.1153	0.3913
PWY.7222..guanosine.deoxyribonucleotides.de.novo.biosynthesis.II	0.0	0.347	0.220	-0.085	0.779	0.1153	0.3913
PWY.7228..superpathway.of.guanosine.nucleotides.de.novo.biosynthesis.I	0.0	0.253	0.161	-0.062	0.568	0.1151	0.3913
PWY.7208..superpathway.of.pyrimidine.nucleobases.salvage	0.0	0.267	0.174	-0.074	0.607	0.1248	0.3995
DAPLYSINESYN.PWY..L.lysine.biosynthesis.I	6.3	-0.565	0.373	-1.295	0.166	0.1298	0.4037
PWY.7357..thiamine.phosphate.formation.from.pyrithiamine.and.oxythiamine..yeast.	0.0	-0.108	0.072	-0.250	0.034	0.1345	0.4072
PWY.6126..superpathway.of.adenosine.nucleotides.de.novo.biosynthesis.II	0.0	0.230	0.158	-0.079	0.540	0.1444	0.4148
PWY0.162..superpathway.of.pyrimidine.ribonucleotides.de.novo.biosynthesis	6.3	0.343	0.235	-0.117	0.803	0.1439	0.4148
PWY.7456...beta...1.4..mannan.degradation	25.0	0.667	0.462	-0.238	1.573	0.1486	0.4161
PWY.5103..L.isoleucine.biosynthesis.III	0.0	-0.093	0.066	-0.222	0.036	0.1588	0.4339
PWY.7328..superpathway.of.UDP.glucose.derived.O.antigen.building.blocks.biosynthesis	0.0	-0.404	0.296	-0.985	0.177	0.1732	0.4505
RIBOSYN2.PWY..flavin.biosynthesis.I..bacteria.and.plants.	6.3	0.298	0.219	-0.130	0.727	0.1723	0.4505
BRANCHED.CHAIN.AA.SYN.PWY..superpathway.of.branched.chain.amino.acid.biosynthesis	0.0	-0.081	0.060	-0.198	0.037	0.1804	0.4505
PWY.7197..pyrimidine.deoxyribonucleotide.phosphorylation	0.0	0.257	0.192	-0.119	0.633	0.1810	0.4505
GLUCOSE1PMETAB.PWY..glucose.and.glucose.1.phosphate.degradation	18.8	-0.484	0.377	-1.222	0.254	0.1984	0.4832
PWY.841..superpathway.of.purine.nucleotides.de.novo.biosynthesis.I	0.0	0.186	0.154	-0.115	0.488	0.2260	0.5385
OANTIGEN.PWY..O.antigen.building.blocks.biosynthesis..E..coli.	0.0	-0.206	0.173	-0.545	0.133	0.2330	0.5437
PWY.5659..GDP.mannose.biosynthesis	0.0	0.186	0.159	-0.124	0.497	0.2395	0.5475
PYRIDNUCSYN.PWY..NAD.de.novo.biosynthesis.I..from.aspartate.	0.0	0.071	0.062	-0.050	0.193	0.2490	0.5529
COLANSYN.PWY..colanic.acid.building.blocks.biosynthesis	0.0	0.164	0.145	-0.119	0.447	0.2567	0.5529
PWY.621..sucrose.degradation.III..sucrose.invertase.	0.0	0.315	0.276	-0.226	0.857	0.2540	0.5529
PWY.8178..pentose.phosphate.pathway..non.oxidative.branch..II	0.0	-0.106	0.096	-0.295	0.083	0.2709	0.5725
PWY.4041...gamma..glutamyl.cycle	6.3	-0.322	0.299	-0.909	0.265	0.2820	0.5733
PWY.6292..superpathway.of.L.cysteine.biosynthesis..mammalian.	12.5	0.226	0.212	-0.190	0.642	0.2867	0.5733
PWY.7229..superpathway.of.adenosine.nucleotides.de.novo.biosynthesis.I	0.0	0.162	0.150	-0.133	0.456	0.2821	0.5733
NONOXIPENT.PWY..pentose.phosphate.pathway..non.oxidative.branch..I	0.0	-0.128	0.124	-0.371	0.115	0.3003	0.5901
PWY0.845..superpathway.of.pyridoxal.5..phosphate.biosynthesis.and.salvage	0.0	0.177	0.173	-0.162	0.515	0.3063	0.5915
PWY.7234..inosine.5..phosphate.biosynthesis.III	6.3	-0.329	0.342	-1.001	0.342	0.3359	0.6377
PWY.3001..superpathway.of.L.isoleucine.biosynthesis.I	0.0	-0.125	0.135	-0.390	0.141	0.3567	0.6468
GLCMANNANAUT.PWY..superpathway.of.N.acetylglucosamine..N.acetylmannosamine.and.N.acetylneuraminate	0.0	0.103	0.117	-0.125	0.332	0.3759	0.6468
GLUCONEO.PWY..gluconeogenesis.I	6.3	0.178	0.204	-0.221	0.577	0.3821	0.6468
P41.PWY..pyruvate.fermentation.to.acetate.and..S..lactate.I	0.0	-0.165	0.191	-0.538	0.209	0.3869	0.6468
PWY.2941..L.lysine.biosynthesis.II	0.0	0.250	0.280	-0.298	0.799	0.3708	0.6468
PWY.5100..pyruvate.fermentation.to.acetate.and.lactate.II	0.0	-0.165	0.191	-0.538	0.209	0.3869	0.6468
PWY.5188..tetrapyrrole.biosynthesis.I..from.glutamate.	0.0	-0.209	0.233	-0.665	0.247	0.3691	0.6468
PWY.8004..Entner.Doudoroff.pathway.I	6.3	0.163	0.187	-0.204	0.530	0.3834	0.6468
ARGSYN.PWY..L.arginine.biosynthesis.I..via.L.ornithine.	0.0	-0.090	0.109	-0.303	0.122	0.4056	0.6680
ARGSYNBSUB.PWY..L.arginine.biosynthesis.II..acetyl.cycle.	0.0	-0.078	0.100	-0.273	0.117	0.4325	0.6920
PWY.6901..superpathway.of.glucose.and.xylose.degradation	6.3	0.156	0.197	-0.230	0.542	0.4290	0.6920
PWY.7392..taxadiene.biosynthesis..engineered.	25.0	0.304	0.396	-0.473	1.081	0.4426	0.6961
CALVIN.PWY..Calvin.Benson.Bassham.cycle	0.0	-0.054	0.074	-0.198	0.090	0.4617	0.6961
GLYCOGENSYNTH.PWY..glycogen.biosynthesis.I..from.ADP.D.Glucose.	0.0	0.072	0.097	-0.118	0.263	0.4564	0.6961
PWY.5989..stearate.biosynthesis.II..bacteria.and.plants.	6.3	-0.173	0.237	-0.636	0.291	0.4661	0.6961
PWY.7199..pyrimidine.deoxyribonucleosides.salvage	0.0	-0.068	0.090	-0.245	0.109	0.4536	0.6961
ANAGLYCOLYSIS.PWY..glycolysis.III..from.glucose.	0.0	0.031	0.045	-0.057	0.119	0.4952	0.7169
PWY.6609..adenine.and.adenosine.salvage.III	0.0	-0.057	0.082	-0.219	0.104	0.4861	0.7164
PWY.6282..palmitoleate.biosynthesis.I..from..5Z..dodec.5.enoate.	12.5	-0.193	0.286	-0.755	0.368	0.4992	0.7169
PWY.7663..gondoate.biosynthesis..anaerobic.	0.0	-0.070	0.106	-0.278	0.138	0.5076	0.7196
PWY.5384..sucrose.degradation.IV..sucrose.phosphorylase.	0.0	0.200	0.315	-0.417	0.817	0.5253	0.7355
FASYN.ELONG.PWY..fatty.acid.elongation....saturated	12.5	-0.144	0.240	-0.615	0.327	0.5492	0.7477
PWY.5121..superpathway.of.geranylgeranyl.diphosphate.biosynthesis.II..via.MEP.	6.3	0.221	0.368	-0.501	0.943	0.5484	0.7477
PWY.5971..palmitate.biosynthesis..type.II.fatty.acid.synthase.	37.5	-0.356	0.642	-1.614	0.902	0.5789	0.7628
PWY.6168..flavin.biosynthesis.III..fungi.	12.5	0.196	0.379	-0.548	0.939	0.6061	0.7724
PWY.6731..starch.degradation.III	0.0	0.198	0.340	-0.468	0.864	0.5608	0.7477

PWY.7664..oleate.biosynthesis.IV..anaerobic.	12.5	-0.138	0.236	-0.600	0.324	0.5586	0.7477
PWY0.1261..anhydromuropeptides.recycling.I	6.3	-0.133	0.259	-0.641	0.374	0.6069	0.7724
PWY66.429..fatty.acid.biosynthesis.initiation..mitochondria.	0.0	-0.038	0.072	-0.179	0.104	0.6023	0.7724
PWY.1042..glycolysis.IV	0.0	0.037	0.084	-0.127	0.201	0.6578	0.8278
PWY.6628..superpathway.of.L.phenylalanine.biosynthesis	6.3	-0.146	0.339	-0.810	0.518	0.6669	0.8300
HISDEG.PWY..L.histidine.degradation.I	0.0	0.067	0.223	-0.369	0.503	0.7632	0.8722
PWY.5941..glycogen.degradation.II	0.0	-0.059	0.182	-0.415	0.298	0.7466	0.8722
PWY.6270..isoprene.biosynthesis.I	6.3	0.080	0.211	-0.333	0.494	0.7026	0.8593
PWY.6317..D.galactose.degradation.I..Leloir.pathway.	0.0	0.036	0.127	-0.213	0.286	0.7750	0.8767
PWY.6700..queuosine.biosynthesis.I..de.novo.	0.0	-0.034	0.093	-0.217	0.148	0.7117	0.8593
PWY.7345..superpathway.of.anaerobic.sucrose.degradation	6.3	0.107	0.303	-0.488	0.702	0.7243	0.8630
PWY.7560..methylerythritol.phosphate.pathway.II	6.3	0.072	0.195	-0.311	0.454	0.7135	0.8593
PWY.8187..L.arginine.degradation.XIII..reductive.Stickland.reaction.	6.3	0.109	0.341	-0.559	0.776	0.7499	0.8722
TRNA.CHARGING.PWY..tRNA.charging	0.0	-0.020	0.063	-0.144	0.105	0.7581	0.8722
PWY.5030..L.histidine.degradation.III	12.5	0.085	0.351	-0.603	0.772	0.8086	0.9056
PWY.6305..superpathway.of.putrescine.biosynthesis	6.3	-0.079	0.363	-0.791	0.633	0.8278	0.9179
PWY.1269..CMP.3.deoxy.D.manno.octulosonate.biosynthesis	0.0	0.035	0.216	-0.388	0.457	0.8720	0.9482
PWY0.862...5Z..dodecenoate.biosynthesis.I	12.5	0.047	0.270	-0.482	0.577	0.8609	0.9453
PYRIDOXSYN.PWY..pyridoxal.5..phosphate.biosynthesis.I	0.0	0.034	0.238	-0.433	0.500	0.8869	0.9551
PWY.6124..inosine.5..phosphate.biosynthesis.II	0.0	0.010	0.095	-0.175	0.195	0.9163	0.9682
RHAMCAT.PWY..L.rhamnose.degradation.I	0.0	0.027	0.204	-0.372	0.426	0.8954	0.9551
BIOTIN.BIOSYNTHESIS.PWY..biotin.biosynthesis.I	12.5	0.019	0.299	-0.568	0.606	0.9495	0.9751
PWY.6519..8.amino.7.oxononanoate.biosynthesis.I	12.5	-0.014	0.240	-0.484	0.456	0.9539	0.9751
NONMEVIPP.PWY..methylerythritol.phosphate.pathway.I	0.0	-0.003	0.058	-0.116	0.110	0.9620	0.9751
POLYISOPRENSYN.PWY..polyisoprenoid.biosynthesis..E..coli.	12.5	0.013	0.280	-0.536	0.562	0.9629	0.9751
ARGININE.SYN4.PWY..L.ornithine.biosynthesis.II	0.0	0.008	0.256	-0.493	0.509	0.9751	0.9751
GLYCOLYSIS.E.D..superpathway.of.glycolysis.and.the.Entner.Doudoroff.pathway	6.3	-0.007	0.223	-0.445	0.430	0.9737	0.9751

Non-remission week 52

Pathway	ZP (%)	Coefficient	Std.error	Lower 95% CI	Higher 95% CI	p-Value	q-Value
PWY.6936..seleno.amino.acid.biosynthesis..plants.	0.0	-0.509	0.067	-0.641	-0.377	3.74E-14	4.27E-12
PWY.7117..C4.photosynthetic.carbon.assimilation.cycle..PEPCK.type	0.0	-0.591	0.092	-0.771	-0.410	1.32E-10	7.51E-09
HISDEG.PWY..L.histidine.degradation.I	0.0	1.150	0.204	0.751	1.550	1.61E-08	6.13E-07
PWY0.1297..superpathway.of.purine.deoxyribonucleosides.degradation	8.3	-0.949	0.231	-1.402	-0.495	4.10E-05	0.0012
PWY.5188..tetrapyrrole.biosynthesis.I..from.glutamate.	0.0	-0.779	0.210	-1.190	-0.367	2.07E-04	0.0047
ANAEROFRUCAT.PWY..homolactic.fermentation	0.0	-0.281	0.077	-0.433	-0.129	2.90E-04	0.0055
PWY.621..sucrose.degradation.III..sucrose.invertase.	0.0	-0.329	0.095	-0.514	-0.143	5.11E-04	0.0083
PWY.7977..L.methionine.biosynthesis.IV	0.0	0.107	0.031	0.045	0.168	6.77E-04	0.0096
PWY.5384..sucrose.degradation.IV..sucrose.phosphorylase.	0.0	-0.275	0.092	-0.455	-0.095	0.0028	0.0341
PWY.6122..5.aminoimidazole.ribonucleotide.biosynthesis.II	0.0	-0.189	0.064	-0.315	-0.063	0.0033	0.0341
PWY.6277..superpathway.of.5.aminoimidazole.ribonucleotide.biosynthesis	0.0	-0.189	0.064	-0.315	-0.063	0.0033	0.0341
SER.GLYSYN.PWY..superpathway.of.L.serine.and.glycine.biosynthesis.I	0.0	0.264	0.092	0.085	0.444	0.0039	0.0375
PWY.5030..L.histidine.degradation.III	0.0	1.075	0.380	0.330	1.820	0.0047	0.0391
PWY.5973..cis.vaccenate.biosynthesis	0.0	0.135	0.048	0.041	0.229	0.0048	0.0391
PWY.5121..superpathway.of.geranylgeranyl.diphosphate.biosynthesis.II..via.MEP.	0.0	0.545	0.195	0.163	0.927	0.0051	0.0391
PWY.702..L.methionine.biosynthesis.II	0.0	-0.526	0.196	-0.909	-0.143	0.0071	0.0506
PWY.5981..CDP.diacylglycerol.biosynthesis.III	16.7	-1.639	0.629	-2.871	-0.407	0.0091	0.0570
PWY.6121..5.aminoimidazole.ribonucleotide.biosynthesis.I	0.0	-0.120	0.046	-0.210	-0.029	0.0097	0.0570
PWY.6527..stachyose.degradation	0.0	-0.410	0.159	-0.721	-0.099	0.0098	0.0570
PWY.7184..pyrimidine.deoxyribonucleotides.de.novo.biosynthesis.I	16.7	-0.981	0.381	-1.728	-0.235	0.0100	0.0570
PWY.7663..gondoate.biosynthesis..anaerobic.	0.0	0.136	0.053	0.031	0.240	0.0112	0.0609
GLYCOLYSIS..glycolysis.I..from.glucose.6.phosphate.	0.0	-0.318	0.126	-0.566	-0.070	0.0119	0.0612
PWY.5484..glycolysis.II..from.fructose.6.phosphate.	0.0	-0.301	0.121	-0.539	-0.064	0.0129	0.0615
PWY.6353..purine.nucleotides.degradation.II..aerobic.	8.3	-0.951	0.380	-1.695	-0.206	0.0123	0.0612
ARO.PWY..chorismate.biosynthesis.I	0.0	-0.139	0.058	-0.252	-0.027	0.0154	0.0690
COMPLETE.ARO.PWY..superpathway.of.aromatic.amino.acid.biosynthesis	0.0	-0.155	0.064	-0.280	-0.029	0.0157	0.0690
ARGININE.SYN4.PWY..L.ornithine.biosynthesis.II	0.0	0.895	0.390	0.130	1.660	0.0218	0.0887
HSERMETANA.PWY..L.methionine.biosynthesis.III	0.0	-0.445	0.209	-0.855	-0.036	0.0332	0.1191
MET.SAM.PWY..superpathway.of.S.adenosyl.L.methionine.biosynthesis	41.7	-0.725	0.337	-1.387	-0.064	0.0316	0.1191
NONOXIPENT.PWY..pentose.phosphate.pathway..non.oxidative.branch..I	0.0	-0.356	0.166	-0.681	-0.030	0.0321	0.1191
PWY.4041...gamma..glutamyl.cycle	8.3	-0.507	0.238	-0.974	-0.040	0.0334	0.1191
PWY.2942..L.lysine.biosynthesis.III	0.0	-0.096	0.045	-0.185	-0.007	0.0350	0.1208
PWY.5100..pyruvate.fermentation.to.acetate.and.lactate.II	0.0	-0.289	0.138	-0.560	-0.018	0.0368	0.1232
ASPASN.PWY..superpathway.of.L.aspartate.and.L.asparagine.biosynthesis	0.0	0.310	0.160	-0.004	0.624	0.0533	0.1714
NAGLIPASYN.PWY..lipid.IVA.biosynthesis..E..coli.	8.3	0.767	0.401	-0.018	1.553	0.0556	0.1714
PWY.8073..lipid.IVA.biosynthesis..P..putida.	8.3	0.767	0.401	-0.018	1.553	0.0556	0.1714
PWY.4984..urea.cycle	0.0	0.705	0.371	-0.022	1.432	0.0572	0.1717
RIBOSYN2.PWY..flavin.biosynthesis.I..bacteria.and.plants.	0.0	0.182	0.097	-0.009	0.373	0.0612	0.1789
ILEUSYN.PWY..L.isoleucine.biosynthesis.I..from.threonine.	0.0	-0.209	0.116	-0.436	0.018	0.0715	0.2021
PWY66.409..superpathway.of.purine.nucleotide.salvage	16.7	-0.920	0.513	-1.925	0.085	0.0727	0.2021
X1CMET2.PWY..folate.transformations.III..E..coli.	0.0	0.043	0.024	-0.005	0.090	0.0789	0.2143
BRANCHED.CHAIN.AA.SYN.PWY..superpathway.of.branched.chain.amino.acid.biosynthesis	0.0	-0.203	0.119	-0.437	0.030	0.0873	0.2314
FERMENTATION.PWY..mixed.acid.fermentation	8.3	-0.481	0.288	-1.045	0.083	0.0947	0.2453
PWY.5097..L.lysine.biosynthesis.VI	0.0	-0.202	0.122	-0.441	0.037	0.0982	0.2488
P41.PWY..pyruvate.fermentation.to.acetate.and..S..lactate.I	0.0	-0.213	0.135	-0.477	0.051	0.1137	0.2787
PYRIDNUCSYN.PWY..NAD.de.novo.biosynthesis.I..from.aspartate.	0.0	0.137	0.087	-0.033	0.308	0.1149	0.2787
PWY.6163..chorismate.biosynthesis.from.3.dehydroquinate	0.0	-0.123	0.080	-0.280	0.034	0.1257	0.2986
PWY.6317..D.galactose.degradation.I..Leloir.pathway.	0.0	-0.192	0.134	-0.455	0.071	0.1519	0.3535
PWY.6630..superpathway.of.L.tyrosine.biosynthesis	0.0	-0.205	0.145	-0.488	0.079	0.1571	0.3583
PWY.6387..UDP.N.acetylmuramoyl.pentapeptide.biosynthesis.I..meso.diaminopimelate.containing.	0.0	-0.125	0.091	-0.304	0.053	0.1698	0.3761
PWY0.845..superpathway.of.pyridoxal.5..phosphate.biosynthesis.and.salvage	8.3	0.422	0.309	-0.184	1.028	0.1725	0.3761
PWY.5695..inosine.5..phosphate.degradation	0.0	0.099	0.074	-0.047	0.245	0.1822	0.3761
PWY.6608..guanosine.nucleotides.degradation.III	0.0	-0.543	0.403	-1.332	0.247	0.1780	0.3761
PYRIDOXSYN.PWY..pyridoxal.5..phosphate.biosynthesis.I	8.3	0.331	0.249	-0.158	0.820	0.1848	0.3761
VALSYN.PWY..L.valine.biosynthesis	0.0	-0.130	0.097	-0.321	0.061	0.1811	0.3761
HISTSYN.PWY..L.histidine.biosynthesis	0.0	0.147	0.116	-0.080	0.374	0.2037	0.3902
PWY.7221..guanosine.ribonucleotides.de.novo.biosynthesis	0.0	-0.103	0.081	-0.262	0.056	0.2035	0.3902
PWY.7282..4.amino.2.methyl.5.diphosphomethylpyrimidine.biosynthesis.II	0.0	-0.180	0.141	-0.456	0.096	0.2015	0.3902

TRNA.CHARGING.PWY..tRNA.charging	0.0	-0.102	0.081	-0.259	0.056	0.2067	0.3902
PWY.7953..UDP.N.acetylmuramoyl.pentapeptide.biosynthesis.III..meso.diaminopimelate.containing.	0.0	-0.118	0.094	-0.303	0.066	0.2088	0.3902
COA.PWY..coenzyme.A.biosynthesis.I..prokaryotic.	0.0	-0.108	0.094	-0.292	0.075	0.2470	0.4202
COLANSYN.PWY..colanic.acid.building.blocks.biosynthesis	0.0	-0.118	0.103	-0.321	0.085	0.2545	0.4212
GLYCOCAT.PWY..glycogen.degradation.I	0.0	-0.214	0.177	-0.561	0.134	0.2281	0.4119
GLYCOGENSYNTH.PWY..glycogen.biosynthesis.I..from.ADP.D.Glucose.	0.0	-0.099	0.084	-0.264	0.066	0.2376	0.4119
PEPTIDOGLYCANSYN.PWY..peptidoglycan.biosynthesis.I..meso.diaminopimelate.containing.	0.0	-0.107	0.088	-0.279	0.065	0.2242	0.4119
PHOSLIPSYN.PWY..superpathway.of.phospholipid.biosynthesis.I..bacteria.	0.0	0.220	0.187	-0.146	0.586	0.2384	0.4119
PWY.6386..UDP.N.acetylmuramoyl.pentapeptide.biosynthesis.II..lysine.containing.	0.0	-0.103	0.087	-0.273	0.068	0.2373	0.4119
PWY.I9..L.cysteine.biosynthesis.VI..from.L.methionine.	8.3	-0.473	0.416	-1.288	0.342	0.2549	0.4212
PWY.6385..peptidoglycan.biosynthesis.III..mycobacteria.	0.0	-0.087	0.077	-0.238	0.064	0.2603	0.4239
PWY.5659..GDP.mannose.biosynthesis	0.0	-0.199	0.192	-0.574	0.177	0.2999	0.4771
PWY.7345..superpathway.of.anaerobic.sucrose.degradation	0.0	-0.140	0.135	-0.404	0.125	0.3013	0.4771
PWY.7761..NAD.salvage.pathway.II..PNC.IV.cycle.	0.0	-0.361	0.373	-1.093	0.370	0.3329	0.5198
PWY.6124..inosine.5..phosphate.biosynthesis.II	0.0	0.062	0.070	-0.075	0.200	0.3735	0.5753
CITRULBIO.PWY..L.citrulline.biosynthesis	0.0	0.263	0.312	-0.349	0.876	0.3992	0.6068
DAPLYSINESYN.PWY..L.lysine.biosynthesis.I	8.3	-0.270	0.337	-0.930	0.390	0.4224	0.6335
PWY.6123..inosine.5..phosphate.biosynthesis.I	0.0	0.044	0.057	-0.066	0.155	0.4330	0.6410
NONMEVIPP.PWY..methylerythritol.phosphate.pathway.I	0.0	0.076	0.109	-0.138	0.289	0.4868	0.6851
PWY.5686..UMP.biosynthesis.I	0.0	-0.062	0.095	-0.249	0.125	0.5179	0.6900
PWY.6700..queuosine.biosynthesis.I..de.novo.	0.0	-0.029	0.041	-0.109	0.051	0.4797	0.6836
PWY.6703..preQ0.biosynthesis	0.0	0.154	0.244	-0.324	0.631	0.5285	0.6926
PWY.724..superpathway.of.L.lysine..L.threonine.and.L.methionine.biosynthesis.II	0.0	-0.039	0.052	-0.142	0.063	0.4514	0.6576
PWY.7790..UMP.biosynthesis.II	0.0	-0.062	0.095	-0.249	0.125	0.5179	0.6900
PWY.7791..UMP.biosynthesis.III	0.0	-0.062	0.095	-0.249	0.125	0.5179	0.6900
PWY.8004..Entner.Doudoroff.pathway.I	0.0	-0.283	0.379	-1.025	0.460	0.4557	0.6576
PWY0.862...5Z..dodecenoate.biosynthesis.I	8.3	0.174	0.270	-0.356	0.703	0.5205	0.6900
RHAMCAT.PWY..L.rhamnose.degradation.I	0.0	0.127	0.190	-0.245	0.499	0.5038	0.6900
PWY.6628..superpathway.of.L.phenylalanine.biosynthesis	0.0	-0.168	0.334	-0.823	0.487	0.6148	0.7964
PWY.6282..palmitoleate.biosynthesis.I..from..5Z..dodec.5.enoate.	8.3	-0.143	0.304	-0.738	0.452	0.6376	0.8063
PWY4FS.7..phosphatidylglycerol.biosynthesis.I..plastidic.	0.0	-0.136	0.297	-0.719	0.447	0.6472	0.8063
PWY4FS.8..phosphatidylglycerol.biosynthesis.II..non.plastidic.	0.0	-0.136	0.297	-0.719	0.447	0.6472	0.8063
DTDPRHAMSYN.PWY..dTDP..beta..L.rhamnose.biosynthesis	0.0	-0.079	0.173	-0.419	0.261	0.6507	0.8063
FASYN.ELONG.PWY..fatty.acid.elongation....saturated	8.3	-0.141	0.320	-0.769	0.486	0.6586	0.8073
PWY.5989..stearate.biosynthesis.II..bacteria.and.plants.	8.3	-0.127	0.308	-0.730	0.476	0.6792	0.8138
THISYNARA.PWY..superpathway.of.thiamine.diphosphate.biosynthesis.III..eukaryotes.	0.0	0.022	0.053	-0.083	0.126	0.6821	0.8138
PWY.7664..oleate.biosynthesis.IV..anaerobic.	8.3	-0.119	0.292	-0.692	0.455	0.6853	0.8138
COA.PWY.1..superpathway.of.coenzyme.A.biosynthesis.III..mammals.	0.0	0.032	0.082	-0.130	0.193	0.7005	0.8217
PWY.3841..folate.transformations.II..plants.	0.0	-0.022	0.058	-0.136	0.092	0.7064	0.8217
PENTOSE.P.PWY..pentose.phosphate.pathway	0.0	-0.064	0.197	-0.450	0.321	0.7438	0.8395
PWY.5154..L.arginine.biosynthesis.III..via.N.acetyl.L.citrulline.	0.0	0.051	0.155	-0.253	0.356	0.7411	0.8395
PWY0.1261..anhydromuropeptides.recycling.I	8.3	0.117	0.356	-0.581	0.816	0.7422	0.8395
PANTO.PWY..phosphopantothenate.biosynthesis.I	0.0	0.017	0.067	-0.114	0.148	0.8026	0.8859
PWY.6519..8.amino.7.oxononanoate.biosynthesis.I	8.3	-0.062	0.253	-0.558	0.434	0.8054	0.8859
PWY.7851..coenzyme.A.biosynthesis.II..eukaryotic.	0.0	0.025	0.101	-0.173	0.222	0.8082	0.8859
POLYISOPRENSYN.PWY..polyisoprenoid.biosynthesis..E..coli.	0.0	0.081	0.356	-0.617	0.778	0.8202	0.8906
BIOTIN.BIOSYNTHESIS.PWY..biotin.biosynthesis.I	8.3	-0.054	0.251	-0.546	0.438	0.8294	0.8920
GLUCOSE1PMETAB.PWY..glucose.and.glucose.1.phosphate.degradation	25.0	0.090	0.441	-0.775	0.955	0.8387	0.8935
COBALSYN.PWY..superpathway.of.adenosylcobalamin.salvage.from.cobinamide.I	0.0	-0.023	0.161	-0.339	0.294	0.8884	0.9254
GLCMANNANAUT.PWY..superpathway.of.N.acetylglucosamine..N.acetylmannosamine.and.N.acetylneuraminate	0.0	0.034	0.272	-0.500	0.568	0.9010	0.9254
PANTOSYN.PWY..superpathway.of.coenzyme.A.biosynthesis.I..bacteria.	0.0	0.013	0.084	-0.151	0.177	0.8804	0.9254
PWY.6895..superpathway.of.thiamine.diphosphate.biosynthesis.II	16.7	0.051	0.386	-0.707	0.808	0.8958	0.9254
PWY.1269..CMP.3.deoxy.D.manno.octulosonate.biosynthesis	8.3	0.025	0.244	-0.452	0.503	0.9175	0.9339
PWY.6901..superpathway.of.glucose.and.xylose.degradation	0.0	-0.012	0.177	-0.359	0.335	0.9453	0.9481
PWY66.429..fatty.acid.biosynthesis.initiation..mitochondria.	0.0	0.003	0.047	-0.089	0.095	0.9481	0.9481
