# Supplementary material for: Phage-Display Immunoprecipitation Sequencing Reveals Distinct Antibody Signatures Against Bacterial Flagellins Associated With Treatment Response in Crohn's Disease
Source: Clin Transl Gastroenterol. 2026 Apr 2;17(6):e01030. doi: 10.14309/ctg.0000000000001030 (PMC13641668; doi:10.14309/ctg.0000000000001030)
Supplement: Supplementary file 8 [file ct9-17-e01030-s008.pdf]

Supplementary Information:

Supplementary Table 1

Information on the complete library of the 55 antibody-bound peptides from bacterial flagellins. The table shows the library peptide ID, original protein, peptide length, starting position of the peptide, peptide frequencies in patients with CD achieving or not-achieving remission, and their ratios

Supplementary Table 2

Associations of bacterial species with remission and non-remission to biologic treatment at week 14, using multivariate analysis by linear models [MaAsLin2] adjusted for age, sex and body mass index. q-value<0.05 considered significant. ZP = zero proportion, Std.error = Standard error, CI = Confidence interval

Supplementary Table 3

Associations of bacterial species with remission and non-remission to biologic treatment at week 22, using multivariate analysis by linear models [MaAsLin2] adjusted for age, sex and body mass index. q-value<0.05 considered significant. ZP = zero proportion, Std.error = Standard error, CI = Confidence interval

Supplementary Table 4

Associations of bacterial species with remission and non-remission to biologic treatment at week 52, using multivariate analysis by linear models [MaAsLin2] adjusted for age, sex and body

mass index. q-value<0.05 considered significant. ZP = zero proportion, Std.error = Standard error, CI = Confidence interval

Supplementary Table 5

Associations of microbial MetaCyc pathways with remission and non-remission to biologic treatment at week 14, using multivariate analysis by linear models [MaAsLin2] adjusted for age, sex and body mass index. q-value<0.05 considered significant. ZP = zero proportion, Std.error = Standard error, CI = Confidence interval

Supplementary Table 6

Associations of microbial MetaCyc pathways with remission and non-remission to biologic treatment at week 22, using multivariate analysis by linear models [MaAsLin2] adjusted for age, sex and body mass index. q-value<0.05 considered significant. ZP = zero proportion, Std.error = Standard error, CI = Confidence interval

Supplementary Table 7

Associations of microbial MetaCyc pathways with remission and non-remission to biologic treatment at week 52, using multivariate analysis by linear models [MaAsLin2] adjusted for age, sex and body mass index. q-value<0.05 considered significant. ZP = zero proportion, Std.error = Standard error, CI = Confidence interval
