# Supplementary material for: Phage-Display Immunoprecipitation Sequencing Reveals Distinct Antibody Signatures Against Bacterial Flagellins Associated With Treatment Response in Crohn's Disease
Source: Clin Transl Gastroenterol. 2026 Apr 2;17(6):e01030. doi: 10.14309/ctg.0000000000001030 (PMC13641668; doi:10.14309/ctg.0000000000001030)
Supplement: Supplementary file 9 [file ct9-17-e01030-s009.pdf]

Supplementary Figure 1 – Antibody responses in responders vs. non-responders at all time-points

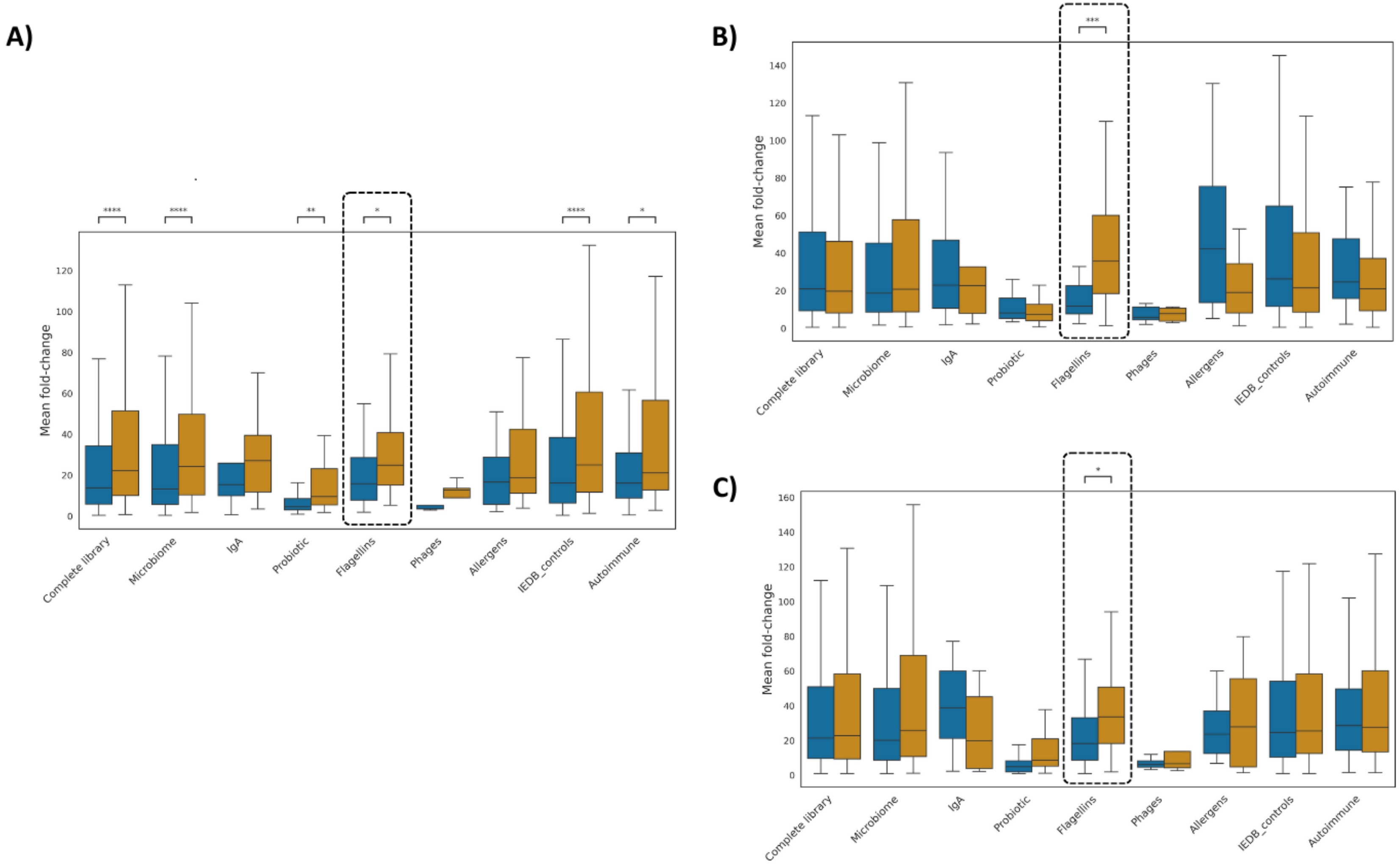

Box plots showing the mean fold change of antibody responses for all libraries at week 14 (**Panel A**), week 22 (**Panel B**) and week 52 (**Panel C**), comparing patients achieving remission (blue colour) versus patients not achieving remission (yellow colour) after commencing biologic treatment. Adjusted p-values are calculated using two-sided Mann-Whitney U test with Benjamini-Hochberg correction. *: 0.01<p<0.05, **: 0.001<p<0.01, ***: 0.0001<p<0.001

Supplementary Figure 2 - Microbial diversity and individual species abundance alterations in patients achieving remission and not achieving remission on biologic treatment at week 14

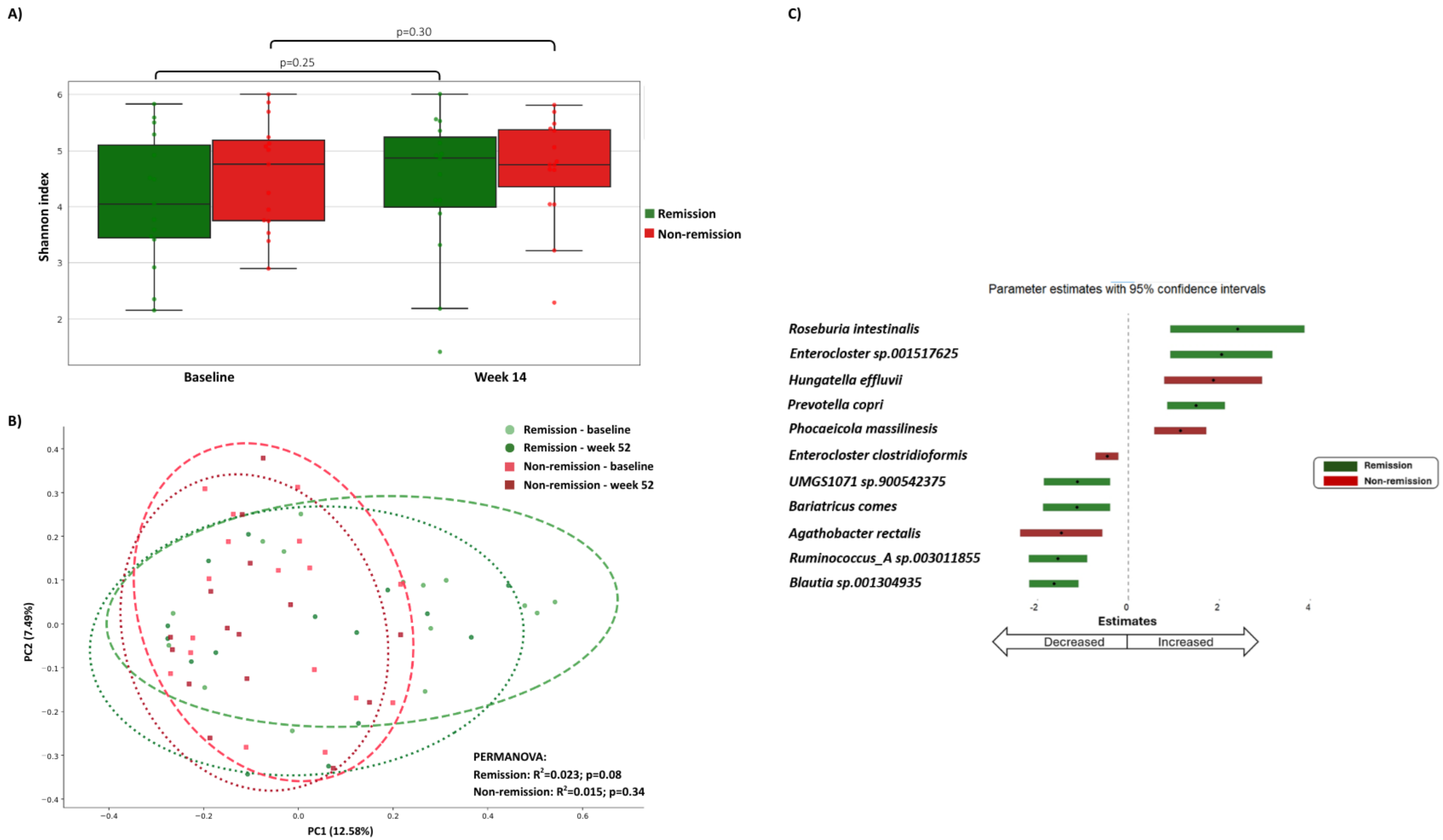

A) Alpha diversity expressed by the Shannon index based on remission at week 14 (analysis was performed on the paired samples; remission [green colour], n=15 versus non-remission [red colour], n=15). p-value is calculated using the Wilcoxon signed-rank sum test. **B)** Beta diversity assessed with principal coordinates analysis plots at the species level based on Bray-Curtis dissimilarity index. Comparisons were analyzed within each group on the paired samples – remission (n=15) at week 14 (green squares) compared baseline (green dots); non-remission (n=15) at week 14 (red squares) compared to baseline (red dots). Ellipsoids represent a 95% confidence interval. Permutation analysis of variance (PERMANOVA) was applied on distance matrices with 1000 permutations with patient ID defined as paired strata. **C)** Coefficient plot showing the effect size and 95% confidence interval (x-axis) for the species found to be significantly differentially abundant (q-values<0.05) following treatment initiation at week 14 in patients achieving remission (n=15) (blue colour) and non-remission (n=15) (yellow colour) (y-axis), based on MaAsLin2 adjusted for age, gender and body mass index

Supplementary Figure 3 - Microbial diversity and individual species abundance alterations in patients achieving remission and not achieving remission on biologic treatment at week 22

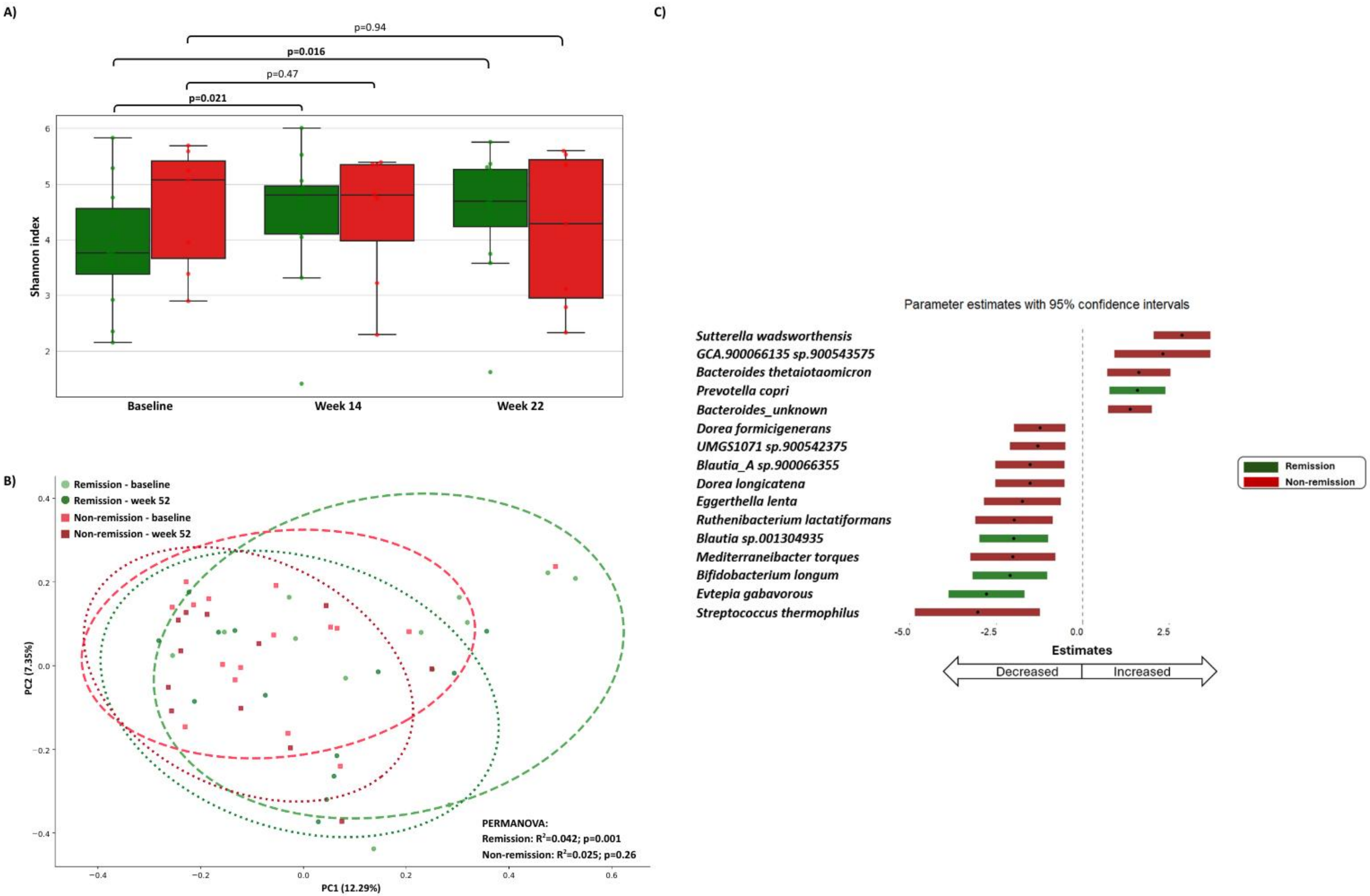

A) Alpha diversity expressed by the Shannon index based on remission at week 22 (analysis was performed on the paired samples available at all time points; remission [green colour], n=12 versus non-remission [red colour], n=7). p-value is calculated using the Wilcoxon signed-rank sum test. **B)** Beta diversity assessed with principal coordinates analysis plots at the species level based on Bray-Curtis dissimilarity index. Comparisons were analyzed within each group on the paired samples – remission (n=13) at week 22 (green squares) compared baseline (green dots); non-remission (n=12) at week 22 (red squares) compared to baseline (red dots). Ellipsoids represent a 95% confidence interval. Permutation analysis of variance (PERMANOVA) was applied on distance matrices with 1000 permutations with patient ID defined as paired strata. Significant shifts from baseline were noted only in patients achieving remission at week 22. **C)** Coefficient plot showing the effect size and 95% confidence interval (x-axis) for the species found to be significantly differentially abundant (q-values<0.05) following treatment initiation at week 22 in patients achieving remission (n=13) (blue colour) and non-remission (n=12) (yellow colour) (y-axis), based on MaAsLin2 adjusted for age, gender and body mass index

Supplementary Figure 4 – Species showing consistent direction of effect in remission and non-remission

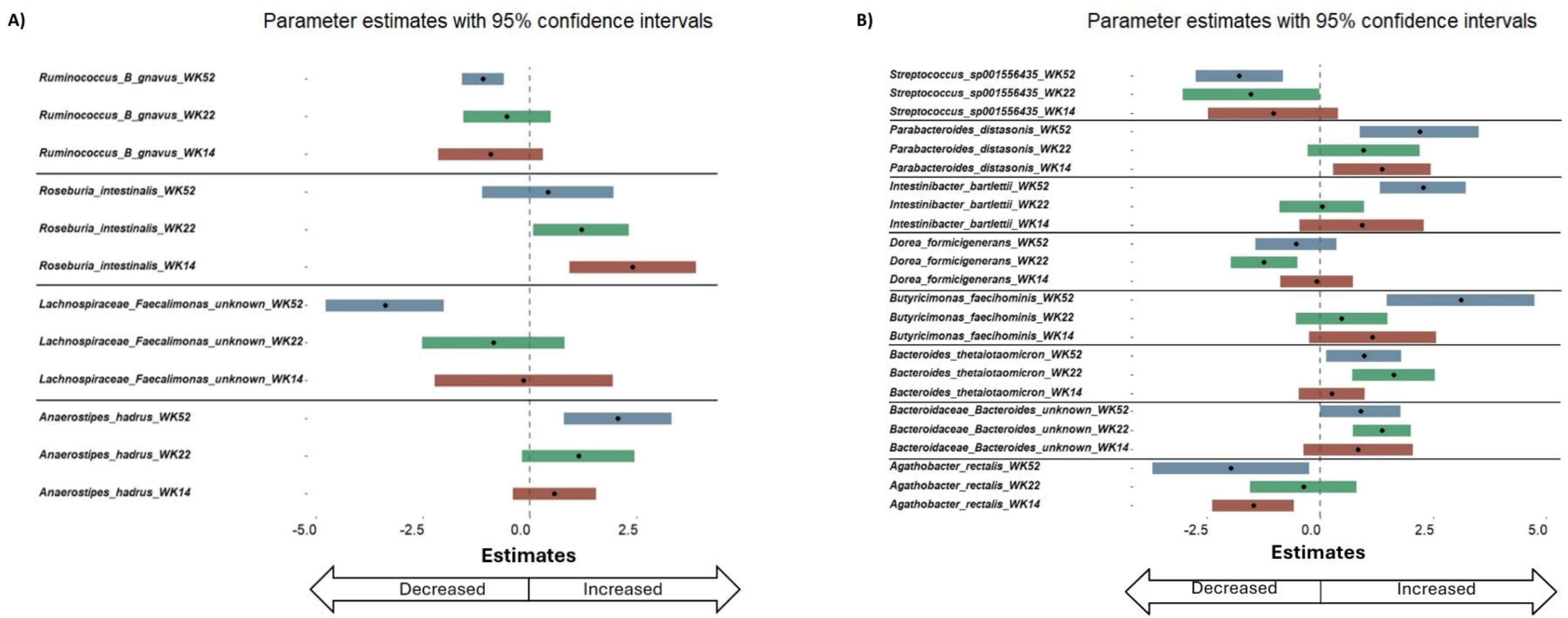

Coefficient plots showing the effect size and 95% confidence interval (x-axis) for the species found to be consistently differentially abundant (consistent direction of effect with at least one time point with q-values<0.05) following treatment initiation at weeks 14 (red colour), 22 (green colour) and 52 (blue colour) in patients achieving remission (**Panel A**) and non-remission (**Panel B**) (y-axis), based on MaAsLin2 adjusted for age, gender and body mass index. **UMGS1071_sp900542375* showed consistent direction of effect of decreased abundance in both remission and non-remission and was not included in the plot (Supplementary Tables 2-4)

Supplementary Figure 5 – No difference in the abundance of taxa containing flagella at each time point between patients in remission compared to non-remission

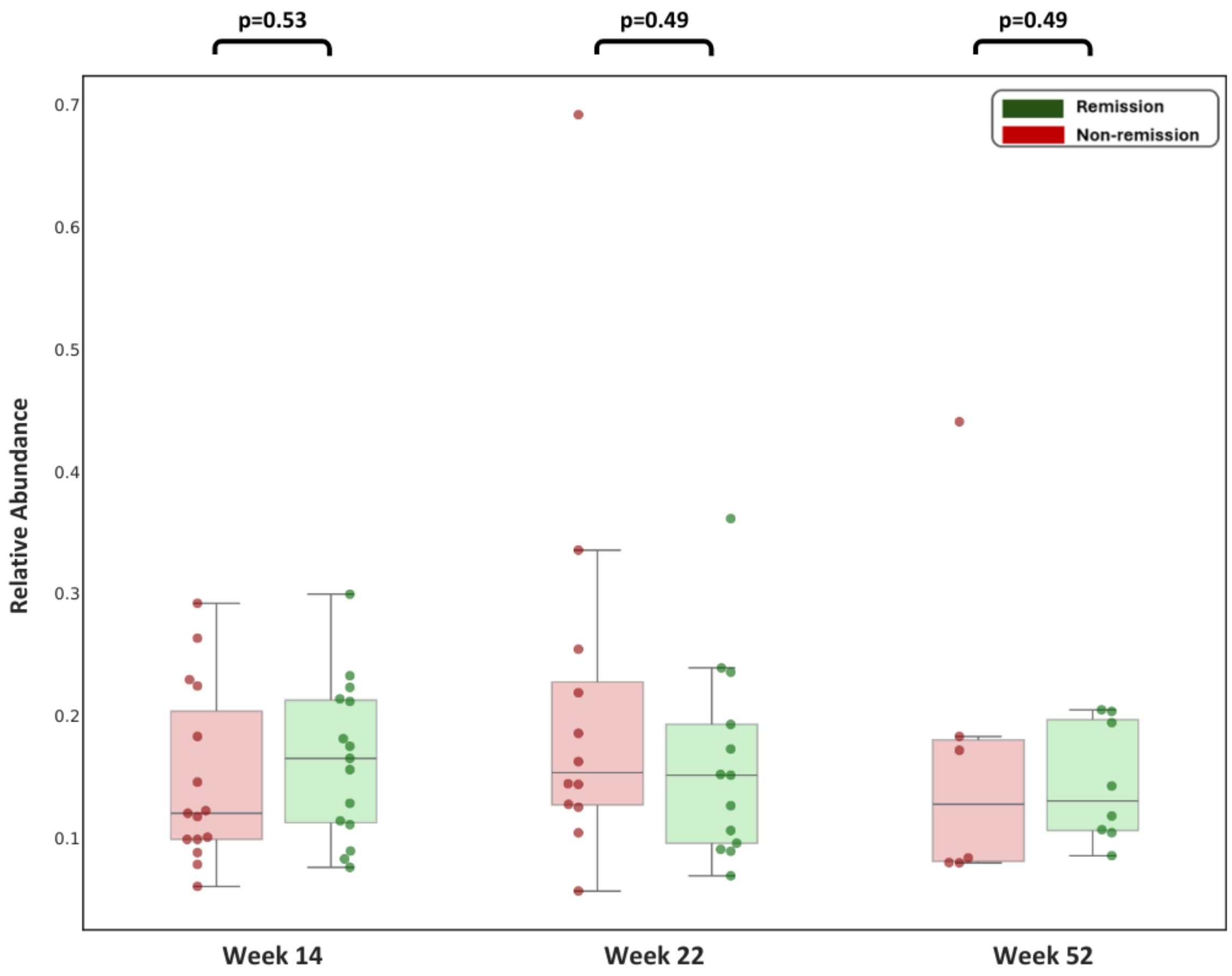

Box plots showing the relative abundance of only taxa containing flagella at each time point comparing patients achieving remission (green colour) versus patients not achieving remission (red colour). p-value is calculated using the Wilcoxon rank sum test

Supplementary Figure 6 – Microbial metabolic pathways showing consistent direction of effect in remission and non-remission

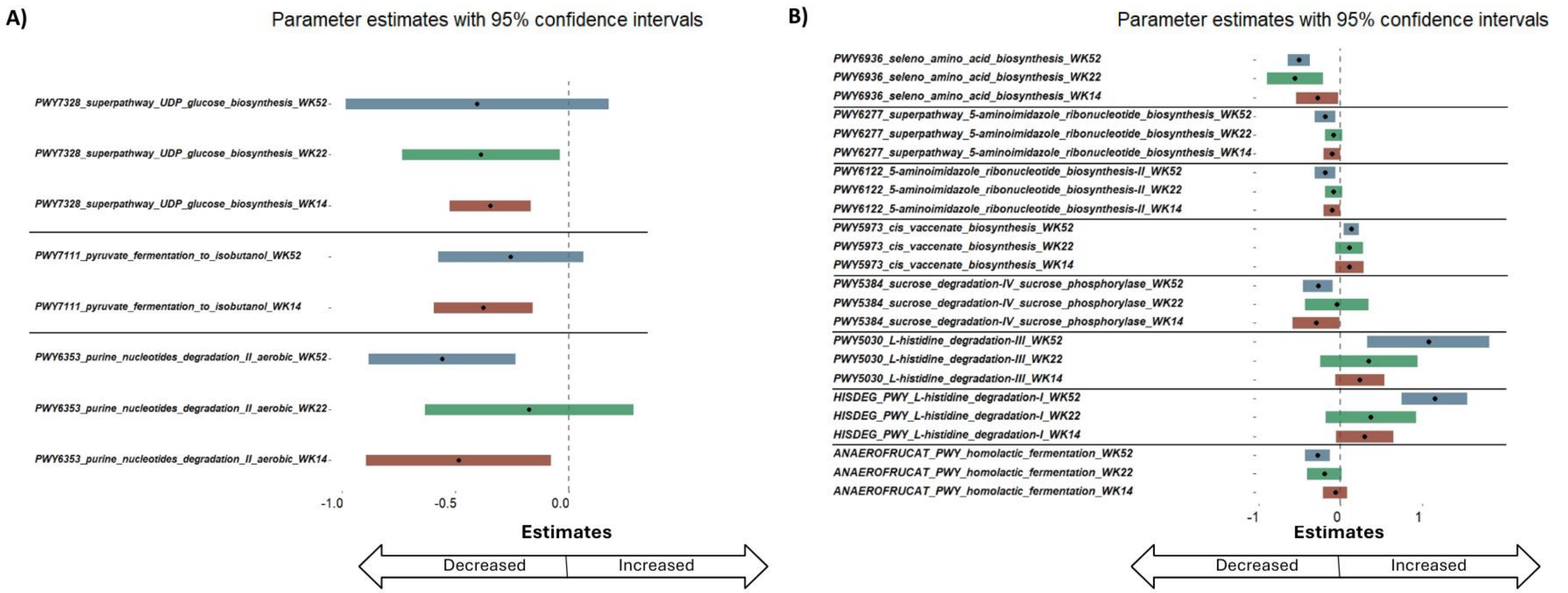

Coefficient plots showing the effect size and 95% confidence interval (x-axis) for the microbial MetaCyc pathways found to be consistently differentially abundant (consistent direction of effect with at least one time point with q-values<0.05) following treatment initiation at weeks 14 (red colour), 22 (green colour) and 52 (blue colour) in patients achieving remission (**Panel A**) and non-remission (**Panel B**) (y-axis), based on MaAsLin2 adjusted for age, gender and body mass index
